# ZnFe_2_O_4_@SiO_2_@L-lysine@SO_3_H: preparation, characterization, and its catalytic applications in the oxidation of sulfides and synthesis of Bis(pyrazolyl)methanes

**DOI:** 10.1038/s41598-024-57317-2

**Published:** 2024-03-28

**Authors:** Amir Ghanbarpour, Arash Ghorbani-Choghamarani, Hamid Aghavandi, Ahmad Jafari

**Affiliations:** Department of Organic Chemistry, Faculty of Chemistry and Petroleum Sciences, Bu-Ali Sina University, Hamedan, 6517838683 Iran

**Keywords:** Catalyst, ZnFe_2_O_4_, L-lysine, SO_3_H, Sulfoxide, Pyrazolyl, Heterogeneous catalysis, Synthetic chemistry methodology

## Abstract

Herein, we report the synthesis of ZnFe_2_O_4_@SiO_2_@L-lysine@SO_3_H as a green, novel magnetic nanocatalyst, containing the sulfuric acid catalytic sites on the surface of zinc ferrite as the catalytic support. The physical and chemical properties of raw and modified samples (ZnFe_2_O_4_@SiO_2_@L-lysine@SO_3_H) were characterized by TGA, EDX, PXRD, Map, and FTIR analyses. The prepared nanocatalyst has excellent catalytic activity in synthesizing the oxidation of sulfides to the sulfoxides and Synthesis of pyrazolyl (Bis(pyrazolyl)methane) derivatives under green conditions. This designed nanocatalyst offers several advantages including the use of inexpensive materials and high yield, simple procedure, and commercially available. The synthesized mesoporous nanocatalyst was recovered and reused in five continuous cycles without considerable change in its catalytic activity.

## Introduction

In recent years, the development of green and environmentally friendly catalytic methods, along with a unique design to improve the reaction process, has attracted the attention of scientists^1–4^. In modern research, the recovery and reusability of catalysts is an important challenge because the used catalysts are often very expensive or economically and medicinally valuable^5,6^. Despite the widespread use of these catalysts, the leaching of toxic or expensive metals is one of the negative aspects of using heterogeneous metal-based catalysts in sustainable catalysis phenomena^7–9^. To overcome this problem, the coupling of green catalysts with heterogeneous magnetic materials as catalytic supports for organic reactions seems to be a suitable solution^10,11^. In the past decade, the use of magnetic nanoparticles as catalytic support in the preparation of catalysts in green methods has been considered by scientific researchers^12,13^. As a main member of the ferrite family, ZnFe_2_O_4_ has promising potential for use as novel catalytic support^14–16^. The ZnFe_2_O_4_ MNPs have attracted much attention due to their magnetic properties, abundant resources, environmental intimacy, nontoxicity, and phase stability^17–19^.

Most solid-state acids are heterogenized organic acids and transition metal complexes or acidic ion-exchange polymer resins. In recent years, various types of solid-acid catalysts, i.e. silica sulfuric acid (SSA), magnetic silica sulfuric acid, and boehmite silica sulfuric acid, have been developed using Zolfigol's method^20^.

Recently, pyrazoles and their derivatives have received great attention due to a broad spectrum of pharmacological and biological activities^21,22^. One of the most important ring systems pyrazoles is created in the composition of five-membered rings containing two groups of nitrogen^23^.

Diphenyl sulfides and their derivatives are important in medicinal chemistry, biologically active molecules, and intermediates in organic synthesis^24^. It should be noted that sulfide compounds have wide applications in the treatment of diseases such as Alzheimer's, Parkinson's, cancer, and HIV^25–27^.

In this paper, regarding the advantages of magnetic nanocomposite and their high efficiency in the synthesis of organic compounds, we report the synthesis of ZnFe_2_O_4_@SiO_2_@L-lysine@SO_3_H NPs. Also, we introduced a novel, reusable, eco-friendly, and green magnetically ZnFe_2_O_4_@SiO_2_@L-lysine@SO_3_H composite as a recoverable magnetic catalyst for the efficient oxidation of sulfides and synthesis of pyrazolyl derivatives in short reaction times.

## Experimental

### Materials

All required materials for the synthesis of catalysts, reagents, and solvents have been purchased from Merck or Fluka.

### Preparation of ZnFe_2_O_4_@SiO_2_@L-lysine@SO_3_H

**Figure 1:**
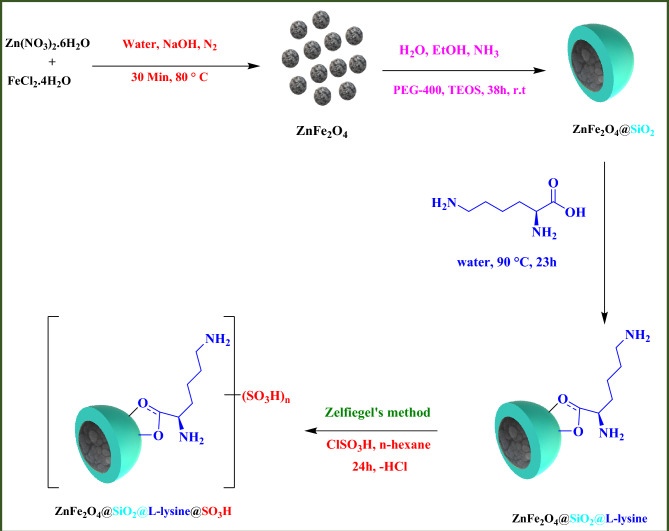
Synthesis of ZnFe_2_O_4_@SiO_2_@L-lysine@SO_3_H.

The ZnFe_2_O_4_ and ZnFe_2_O_4_@SiO_2_ MNPs were prepared according to our previous methods respectively^28,29^. In the next step, the ZnFe_2_O_4_@SiO_2_ (0.5 g) was dispersed in 60 mL **DI** (H_2_O) by sonication for 45 min. After vigorous stirring for 45 min, 1.5 mmol of L-lysine was added to the reaction mixture which was stirred at 60 °C degrees for 22 h. The product was separated by an external Neodymium magnet and washed with Ethanol and H_2_O and dried in an oven at 65 °C degrees to give ZnFe_2_O_4_@SiO_2_@L-lysine composite. Finally, to prepare ZnFe_2_O_4_@SiO_2_@L-lysine@SO_3_H, the obtained ZnFe_2_O_4_@SiO_2_@L-lysine (1 gr) were added to the flask and dispersed ultrasonically for 30 min in dry hexane (35 mL). Chlorosulfonic acid (0.4 mL) was added dropwise to a cooled ice-bath dispersion of ZnFe_2_O_4_@SiO_2_@L-lysine for 35 min. Chlorosulfonic acid was slowly added to the reaction mixture at cool temperature. Then, the reaction mixture was subjected to continuous stirring for 24 h, while the residual HCl was eliminated by suction. The product was then separated from the reaction mixture by an external Neodymium magnet and washed several times with dried hexane. Finally, ZnFe_2_O_4_@SiO_2_@L-lysine@SO_3_H was dried under vacuum at 60 °C (Fig. 1).

### Synthesis procedure for pyrazolyl derivatives

**Figure 2:**
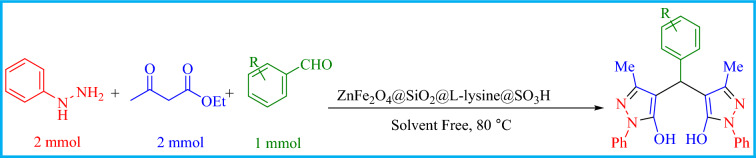
The preparation of the pyrazolyl model reaction.

A mixture of ethyl acetoacetate (2 mmol), phenylhydrazine (2 mmol), and substituted aromatic aldehydes (1 mmol) and ZnFe_2_O_4_@SiO_2_@L-lysine@SO_3_H (0.015 g) at 80 °C under solvent-free conditions for 15 min. After completion of the reaction (checked by TLC), the reaction mixture was diluted with hot EtOH to dissolve the organic products, the catalyst was separated using a magnet and the resultant unrefined pyrazolyl products were further purified through recrystallization in the EtOH Fig. 2.

### A general procedure for the oxidation of sulfides

**Figure 3:**
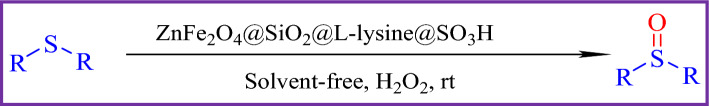
Oxidation of sulfides to sulfoxides catalyzed by ZnFe_2_O_4_@SiO_2_@L-lysine@SO_3_H.

A combination of sulfide (0.5 mmol) and H_2_O_2_ (0.15 mL) containing ZnFe_2_O_4_@SiO_2_@L-lysine@SO_3_H composite as catalyst (0.03 g) was stirred under solvent-free conditions at 25 °C. The progress of the reaction was monitored by TLC. Upon the completion of the reaction, the ZnFe_2_O_4_@SiO_2_@L-lysine@SO_3_H were separated by a magnet, and the products were extracted by **DI** (H_2_O) and EtOAc. The organic phase was dried with Na_2_SO_4_ (Fig. 3).

### Selected NMR data

#### 4,4'-(Pyridin-3-ylmethylene)bis(3-methyl-1-phenyl-1H-pyrazol-5-ol)

^1^H NMR (250 MHz, DMSO): 2.32 (m, 6H), 4.85 (s, 1H), 6.24–7. 69 (m, 14H), 14.06 (s, br, 2H) ppm. ^13^C NMR (62.5 MHz, DMSO): 11.9, 33.6, 104.5, 118.6, 120,3, 125.5, 129.5, 135.2, 137.5, 139.1, 142.5, 147.5, 155.8, 156.1, 158.2 ppm. FT-IR (KBr) cm^−1^: 756, 1032, 1427, 1497, 1609, 3486.

#### 4,4'-(Thiophen-2-ylmethylene)bis(3-methyl-1-phenyl-1H-pyrazol-5-ol)

^1^H NMR (250 MHz, DMSO): 2.30 (m, 6H), 4.89 (s, 1H), 6.80–7. 75 (m, 13H), 14.06 (s, br, 2H) ppm. ^13^C NMR (62.5 MHz, DMSO): 10.8, 34.6, 121.0, 124.1, 124.9, 126.6, 127.1, 128.1, 128.3, 129.2, 138.0, 142.5, 146.1, 149.0 ppm. FT-IR (KBr) cm^−1^: 753, 1380, 1504, 1575, 1600, 2835, 3065.

#### 4,4'-((4-Nitrophenyl)methylene)bis(3-methyl-1-phenyl-1H-pyrazol-5-ol)

^1^H NMR (250 MHz, DMSO): 2.32 (m, 6H), 5.10 (s, 1H), 6.80–8.15 (m, 14H), 13.95 (s, br, 2H) ppm. ^13^C NMR (62.5 MHz, DMSO): 11.0, 35.4, 120.4, 123.3, 126.2, 128.9, 130.0, 138.2, 146.2, 147.0, 150.7 154.8, 155.4, 158.3, 159.9 ppm. FT-IR (KBr) cm^−1^: 756, 1345, 1520, 1605, 2930,3075, 3475.

#### 4,4'-((3-Nitrophenyl)methylene)bis(3-methyl-1-phenyl-1H-pyrazol-5-ol

^1^H NMR (250 MHz, DMSO): 2.29 (m, 6H), 5.24 (s, 1H), 7.29 (t, J = 7.5 Hz, 2H), 7.44 (m, 5H), 7.56 (m, 2H), 7.61 (t, J = 7.5 Hz, 3H), 8.03 (s, 2H), 13.99 (s, br, 2H) ppm. ^13^C NMR (62.5 MHz, DMSO): 10.5, 34.1, 120.5, 120.9, 122.4, 126.7, 129.7,137.3, 137.6 137.9, 139, 145.6, 147.8, 148.3, 157.4 ppm. FT-IR (KBr) cm^−1^: 698, 762, 1351, 1528, 1604, 3086, 3464. Mass analysis: Calculated: M/Z = 481.18, Obtained: M/Z = 481.0

#### 4,4'-((3-Fluorophenyl)methylene)bis(3-methyl-1-phenyl-1H-pyrazol-5-ol)

^1^H NMR (250 MHz, DMSO): 2.34 (m, 6H), 5.26 (s, 1H), 7.05–7.59 (m, 14H), 14.20 (s, br, 2H) ppm^13^C NMR (62.5 MHz, DMSO): 11.4, 33.7, 120.0, 123.2, 1235.1, 125.3, 129.4,129.8, 135.1, 139.1, 145.2, 147.6, 148.8, 150.4, 155.0 ppm.. FT-IR (KBr) cm^−1^:750, 1267, 1423,1495, 1607, 2930, 3078, 3375.

#### 4,4'-((3-Methoxyphenyl)methylene)bis(3-methyl-1-phenyl-1H-pyrazol-5-ol)

^1^H NMR (250 MHz, DMSO): 2.19 (m, 6H), 3.84 (s, 3H), 5.22 (s, 1H), 7.15–7. 27 (m, 8H), 7. 43 (t, J = 7.5, 4H), 7.72 (t, J = 7.5, 2H), 14.44 (s, br, 2H) ppm. ^13^C NMR (62.5 MHz, DMSO): 13.7, 35.1, 58.2, 111.4, 112.5, 120.2, 120.7, 126.0, 126.9, 128.9, 130.4, 137.9, 142.0, 142.7, 149.1, 156.2 ppm.

#### 4,4'-((3-Bromophenyl)methylene)bis(3-methyl-1-phenyl-1H-pyrazol-5-ol)

^1^H NMR (250 MHz, DMSO): 2.30 (m, 6H), 4.66 (s, 1H), 7.29–7. 56 (m, 14H), 14.04 (s, br, 2H) ppm. ^13^C NMR (62.5 MHz, DMSO): 11.7, 35.1, 120.3, 120.4, 125.3, 126.5, 128.1, 130.2, 130.4, 137.9, 146.1, 149.3, 156.2, 156.5, 157.0 ppm.. FT-IR (KBr) cm^−1^:761, 1053, 1427, 1497, 1590, 3087, 3453.

#### 4,4'-(Phenylmethylene)bis(3-methyl-1-phenyl-1H-pyrazol-5-ol)

^1^H NMR (250 MHz, DMSO): 2.32 (m, 6H), 4.64 (s, 1H), 7.36–7. 58 (m, 15H), 14.00 (s, br, 2H) ppm. ^13^C NMR (62.5 MHz, DMSO): 10.2, 35.9, 105.5, 120.0, 125.9, 127.4, 128.5, 129.7, 134.8, 137.4, 142.6, 147.0, 155.1, 157.0, 158.3 ppm.

#### 4,4'-((4-Hydroxyphenyl)methylene)bis(3-methyl-1-phenyl-1H-pyrazol-5-ol)

^1^H NMR (250 MHz, DMSO): 2.30 (m, 6H), 4.58 (s, 1H), 6.82–7. 57 (m, 13H), 8.80 (s, 1H) 14.00 (s, br, 2H) ppm. ^13^C NMR (62.5 MHz, DMSO): 9.0, 34.6, 110.5, 115.4, 119.9, 120.3, 125.2, 127.1, 129.1, 132.5, 137.9, 146.2, 156.4, 157.1 ppm. FT-IR (KBr) cm^−1^: 761, 1503, 1582, 1601, 3177, 3409.

#### 4,4'-((2,4-Dichlorophenyl)methylene)bis(3-methyl-1-phenyl-1H-pyrazol-5-ol)

^1^H NMR (250 MHz, DMSO): 2.25 (m, 6H), 5.07 (s, 1H), 7.24–7.67 (m, 13H), 13.80 (s, br, 2H) ppm. ^13^C NMR (62.5 MHz, DMSO): 10.6, 33.9, 120.6, 125.1, 127.0, 128.2, 129.5, 131.9, 132.2, 137.1, 139.3, 147.1, 155.5, 157.2, 158.5, 159.8 ppm.

#### 4,4'-((2-Methoxyphenyl)methylene)bis(3-methyl-1-phenyl-1H-pyrazol-5-ol)

^1^H NMR (250 MHz, DMSO): 2.29 (m, 6H), 4.49 (s, 1H), 6.79–7.68 (m, 14H), 14.08 (s, br, 2H) ppm. ^13^C NMR (62.5 MHz, DMSO): 11.8, 35.3, 110.8, 114.2, 118.7, 1201.4, 126.4, 129.2, 134.4, 137.5, 144,6, 146.7, 151.1, 156.3, 16.3 ppm. FT-IR (KBr) cm^-1^: 754, 1031, 1502, 1605, 3464.

#### 4,4'-(*p*-Tolylmethylene)bis(3-methyl-1-phenyl-1H-pyrazol-5-ol)

^1^H NMR (250 MHz, DMSO): 2.25 (m, 6H), 4.87 (s, 1H), 7.11–7.66 (m, 14H), 13.91 (s, br, 2H) ppm. FT-IR (KBr) cm^−1^:751, 805, 1027, 1289, 1498, 1606, 3460.

#### 4,4'-((4-Methoxyphenyl)methylene)bis(3-methyl-1-phenyl-1H-pyrazol-5-ol)

^1^H NMR (250 MHz, DMSO): 2.29 (m, 6H), 3.69 (s, 3H), 4.68 (s, 1H), 6.82–7.68 (m, 14H), 13.91 (s, br, 2H) ppm. ^13^C NMR (62.5 MHz, DMSO): 13.0, 34.6, 57.0, 112.8, 122.2, 122.7, 125.5, 127.3, 129.9, 134.5, 137.3, 141.9, 156.2, 158.2, 160.1 ppm.. FT-IR (KBr) cm^−1^:758, 1045, 1278, 1502, 1606, 3074, 3522.

#### 4,4'-((4-Chlorophenyl)methylene)bis(3-methyl-1-phenyl-1H-pyrazol-5-ol)

^1^H NMR (250 MHz, DMSO): 2.30 (m, 6H), 4.56 (s, 1H), 7.28–7.69 (m, 14H), 13.92 (s, br, 2H) ppm. ^13^C NMR (62.5 MHz, DMSO): 11.8, 34.1, 118.5, 121.0, 126.1, 128.3, 129.1, 137.5, 138.5, 147.0, 1455.9, 157.3, 158.1, 159.0 ppm. FT-IR (KBr) cm^−1^: 750, 1293, 1415, 1498, 1607, 3499.

#### 4,4'-((3-Chlorophenyl)methylene)bis(3-methyl-1-phenyl-1H-pyrazol-5-ol)

^1^H NMR (250 MHz, DMSO): 2.26 (m, 6H), 5.12 (s, 1H), 7.25–7.54 (m, 14H), 13.93 (s, br, 2H) ppm. ^13^C NMR (62.5 MHz, DMSO): 10.8, 32.5, 120.3, 121.1, 126.8, 127.2, 128.8, 129.2, 130.3, 131.5, 132.2, 137.6, 139.5, 146.4, 157.4 ppm.FT-IR (KBr) cm^−1^:694, 751, 1363, 1405, 1501, 1557, 1610, 2810, 3528.

#### 4,4'-((3,4-Dimethoxyphenyl)methylene)bis(3-methyl-1-phenyl-1H-pyrazol-5-ol)

^1^H NMR (250 MHz, DMSO): 2.26 (m, 6H), 4.84 (s, 3H), 4.84 (s, 1H), 7.01–7.99 (m, 12H), 8.83 (s, 1H), 14.12 (s, br, 2H) ppm. ^13^C NMR (62.5 MHz, DMSO): 11.1, 35.6, 57.8, 112.0, 116.3, 121.3, 124.6, 125.6, 127.4, 130.6, 131.4, 135.8, 148.6, 139.8, 147.3, 149.6, 152.4 ppm.

#### 4,4'-((1H-indol-3-yl)methylene)bis(3-methyl-1-phenyl-1H-pyrazol-5-ol)

^1^H NMR (250 MHz, DMSO): 2.40 (m, 6H), 4.18 (s, 1H), 6.87–8.34 (m, 15H), 10.05 (s, 1H), 13.56 (s, 2H) ppm. ^13^C NMR (62.5 MHz, DMSO): 13.7, 31.1, 112.9, 114.4, 119.0, 122.4, 123.9, 124.3, 127.4, 128.8, 129.1, 130.0, 133.8, 137.2, 138.6, 140.6, 152.0, 153.4 ppm.

#### Benzyl(phenyl)sulfane

^1^H NMR (250 MHz, DMSO) δ = 4.02 (m, 1H), 4.21 (m, 1H), 7.05–7.66 (m, 10H), ppm. Mass analysis: Calculated: M/Z = 216.06, Obtained: M/Z = 216.1

#### (Ethylsulfinyl)benzene

^1^H NMR (250 MHz, DMSO) δ = 2.64 (m, 3H), 3.18 (m, 2H), 7.53–7.93 (m, 5H), ppm. ^13^C NMR (62.5 MHz, DMSO): 10.5, 34.5, 122.0, 125.3, 126.8, 129.0 ppm.

#### (Methylsulfinyl)benzene

^1^H NMR (250 MHz, DMSO) δ = 4.22 (s, 3H), 7.38–7.83 (m, 5H) ppm. ^13^C NMR (62.5 MHz, DMSO): 43.8, 124.6, 129.2, 133.7, 146.7 ppm.

#### (Sulfinylbis(methylene))dibenzene

^1^H NMR (250 MHz, DMSO) δ = 3.76 (s, 2H), 4.17 (s, 2H), 6.90–7.17 (m, 10H) ppm. ^13^C NMR (62.5 MHz, DMSO): 57.4, 128.1, 129.3, 130.6, 133.6, ppm.

#### 1-(Butylsulfinyl)butane

^1^H NMR (250 MHz, DMSO) δ = 0.97 (m, 6H), 1.40 (s, 4H), 1.53 (s, 4H), 3.04 (m, 10H) ppm. ^13^C NMR (62.5 MHz, DMSO): 13.9, 22.0, 25.6, 51.3 ppm.

#### (Methylsulfinyl)methane

^1^H NMR (250 MHz, DMSO) δ = 3.56 (m, 6H) ppm. ^13^C NMR (62.5 MHz, DMSO): 44.8 ppm.

## Result and discussion

### Catalyst characterization

**Figure 4:**
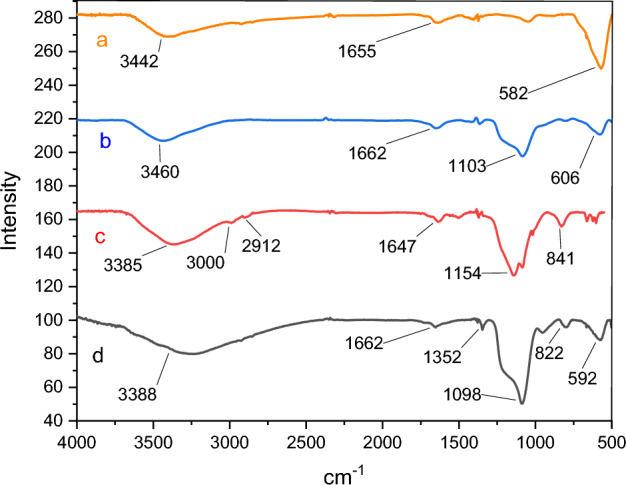
FTIR spectra of (**a**) ZnFe_2_O_4_ (**b**) ZnFe_2_O_4_@SiO_2_ (**c**) ZnFe_2_O_4_@SiO_2_@L-lysine (**d**) ZnFe_2_O_4_@SiO_2_@L-lysine@SO_3_H.

Using FT-IR spectroscopy, the synthesis of zinc ferrite nanoparticles (ZF-NPS) was confirmed. The absorption band at 582 cm^−1^ is assigned to the stretching vibrations of the zinc-oxygen bond^30,31^. In Fig. 4a, the bending and stretching vibration of hydroxyl groups on the surface of the nanoparticles at 1655 and 3442 cm^−1^ are respectively assigned^29^. Figure 4b confirms the condensation reaction between hydroxyl groups of ZnFe_2_O_4_ (MNPs) and the alkoxysilane molecules of tetraethyl orthosilicate (TEOS) as the first layer. Absorbed peaks at 3460 cm^−1^ were specified as hydroxide stretching vibration mode^30^. The two absorption peaks around 1103, and 606 cm^−1^ were indicated the presence of silicon-oxygen )Si–O-Si( asymmetric and symmetric stretching vibrations and bending vibration mode of silicon-oxygen (Si–O-Si), as well as a small peak around 1647 cm^−1^, was assigned to hydroxide stretching vibration of Silicon-hydroxyl group and twisting vibration of adsorbed H–O-H in a silica shell^32^. In Fig. 4c, ZnFe_2_O_4_@SiO_2_@L-lysine, the bands in the range of 2912 to 3000mc^−1^ correspond to the bending vibration of CH_2_ confirming the attachment of L-lysine molecules to the surface. Then, the presence of broad band at 2500–3700 cm^−1^ in FTIR spectra of ZnFe_2_O_4_@SiO_2_@L-lysine@SO_3_H (Fig. 4d) confirms the successful functionalization of ZnFe_2_O_4_@SiO_2_ with the SO_3_H groups^28^.

**Figure 5:**
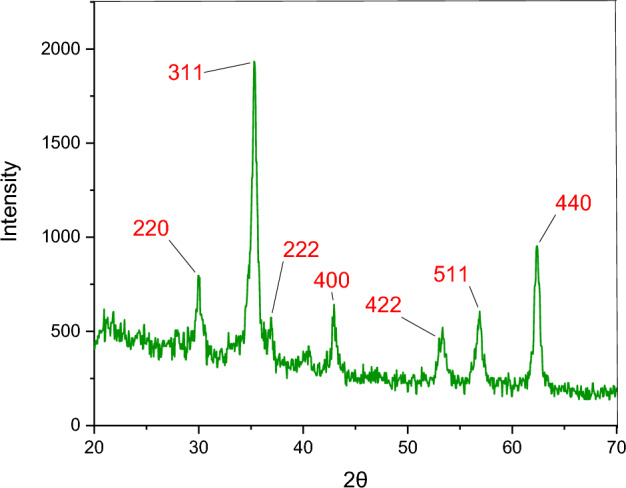
PXRD spectrum of ZnFe_2_O_4_@SiO_2_@L-lysine@SO_3_H.

The PXRD spectra of the ZnFe_2_O_4_@SiO_2_@L-lysine@SO_3_H nanostructures are recorded in a range of Bragg's angle (2θ = 20**°**–70**°**) at room temperature (Fig. 5). The PXRD pattern of the prepared ZnFe_2_O_4_@SiO_2_@L-lysine@SO_3_H shows seven characteristic peaks at 30**°**, 35**°**, 36**°**, 43**°**, 54**°**, 57**°**, and 63**°**, corresponding to the (2 2 0), (3 1 1), (2 2 2), (4 0 0), (4 2 2), (5 1 1), and (4 4 0) which confirms the crystal structure of ZnFe_2_O_4_@SiO_2_@L-lysine@SO_3_H^33^.

**Figure 6:**
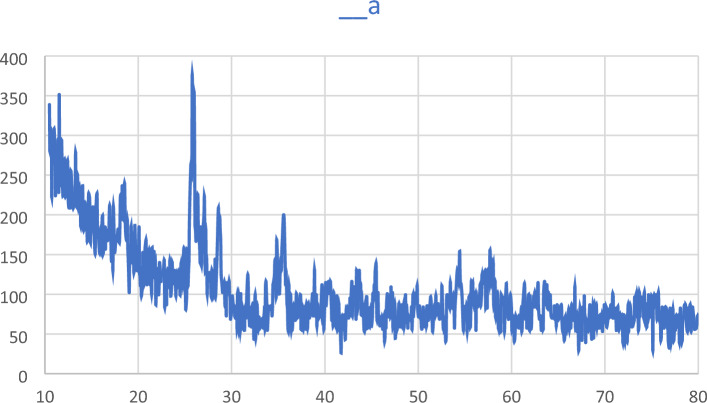
PXRD spectrum of recovered ZnFe_2_O_4_@SiO_2_@L-lysine@SO_3_H nanoparticles.

The PXRD analysis for the used catalyst was provided and the results were compared to the fresh catalyst, which shows high stability of the prepared catalyst under optimized reaction conditions (Fig. 6).

**Figure 7:**
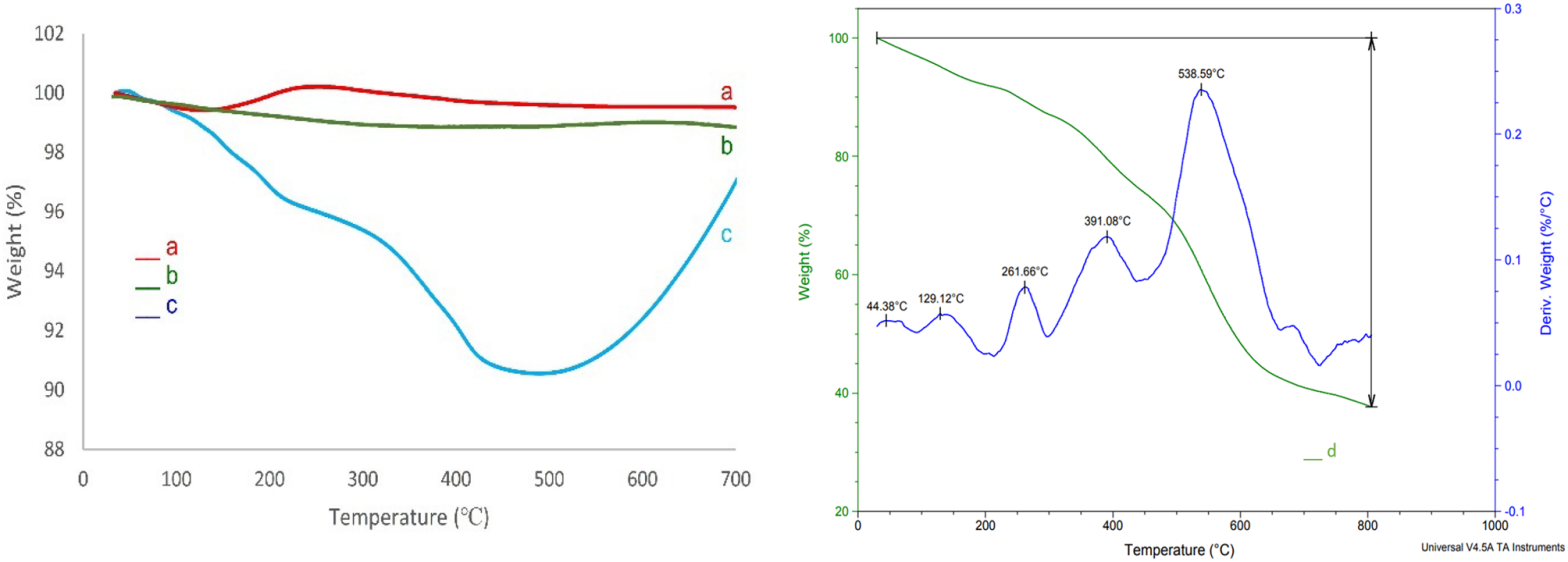
TGA of (**a**) ZnFe_2_O_4_, (**b**) ZnFe_2_O_4_@SiO_2_ (**c**) ZnFe_2_O_4_@SiO_2_@L-lysine, (**d**) ZnFe_2_O_4_@SiO_2_@L-lysine@SO_3_H.

Figure 7 shows the TGA curves for ZnFe_2_O_4_ MNPs, ZnFe_2_O_4_@SiO_2_, ZnFe_2_O_4_@SiO_2_@L-lysine, and ZnFe_2_O_4_@SiO_2_@L-lysine@SO_3_H. In all samples, the first step of weight loss (below 200 °C) is owing to the removal of physically absorbed water and organic solvents (Fig. 7a–d). The decomposition of the organic layer on ZnFe_2_O_4_ has occurred in the TGA curve of the catalyst from 200 to 500 °C. Meanwhile, weight loss of about 2% and 8% from 200 to 500 °C occurred for SiO_2_ and L-lysine, respectively (Fig. 7b and c). Figure 7d illustrates two weight loss steps in the TGA curve of ZnFe_2_O_4_@SiO_2_@L-lysine@SO_3_H. The first weight loss (10%) between 25 and 250 °C is occurred due to the removal of adsorbed moisture and organic solvents. The next weight loss (50%) from 250 to 600 **°**C is due to the degradation of organic moieties and the chemisorbed sulfuric acid groups on the surface of the ZnFe_2_O_4_ core. Based on the results of the TGA–DSC curve, the well grafting of organic groups on the ZnFe_2_O_4_ magnetic nanoparticles is verified^34^.

**Figure 8:**
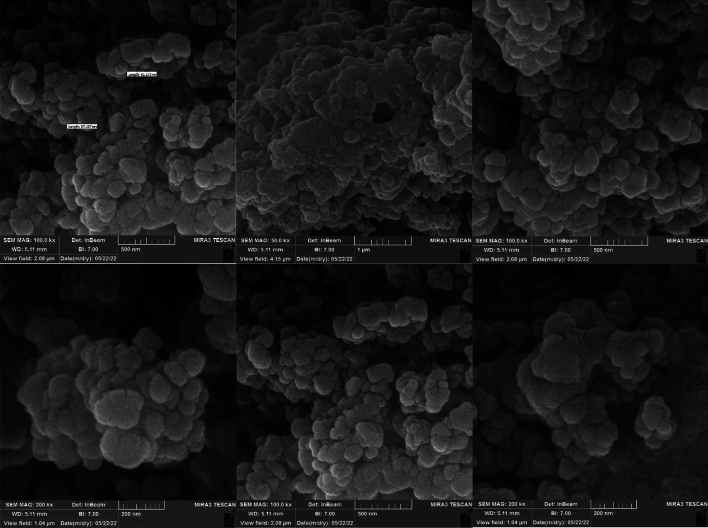
SEM spectrum of ZnFe_2_O_4_@SiO_2_@L-lysine@SO_3_H.

The distribution, size, surface morphology, particle shape, and fundamental physical properties of ZnFe_2_O_4_@SiO_2_@L-lysine@SO_3_H nanoparticles were investigated using the SEM technique (Fig. 8). The ZnFe_2_O_4_@SiO_2_@L-lysine@SO_3_H composite is spherical with an almost homogenous size distribution. In addition, the SEM image shows that the size of the nanoparticles is about ≈ 81 nm (Fig. 8).

**Figure 9:**
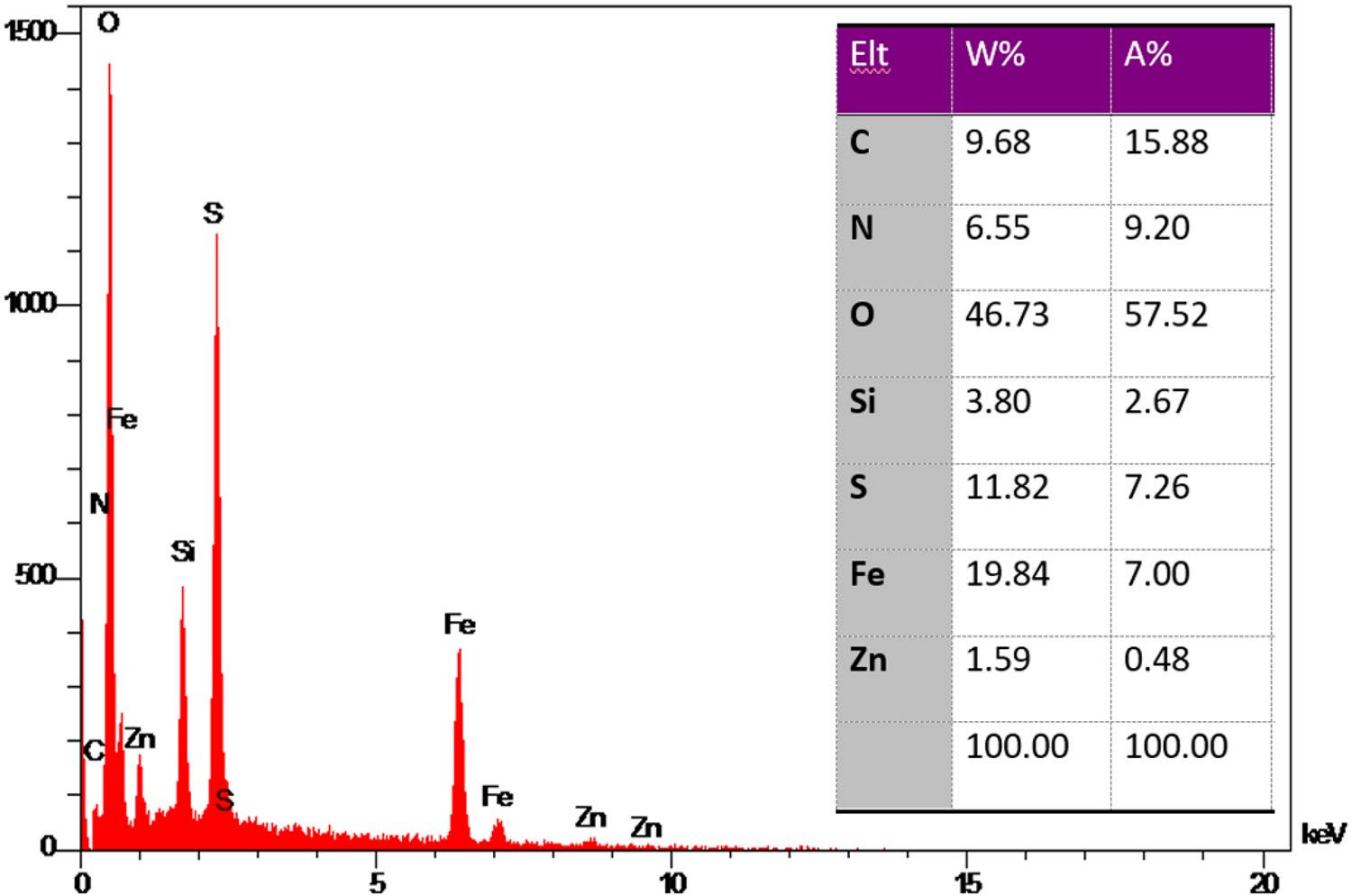
EDS spectrum of ZnFe_2_O_4_@SiO_2_@L-lysine@SO_3_H.

In another investigation, EDX analysis confirmed the presence of Zn, C, O, Si, N, Fe, and S elements in the synthesized ZnFe_2_O_4_@SiO_2_@L-lysine@SO_3_H. As shown in Fig. 9, the presence of Si species confirmed the successful bonding of the SiO_2_ shell on the ZnFe_2_O_4_ catalytic support. The high purity of the synthesized nanocatalyst was confirmed by these observations. It can be concluded that the target catalyst has been successfully synthesized according to this EDX spectrum (Fig. 9).

**Figure 10:**
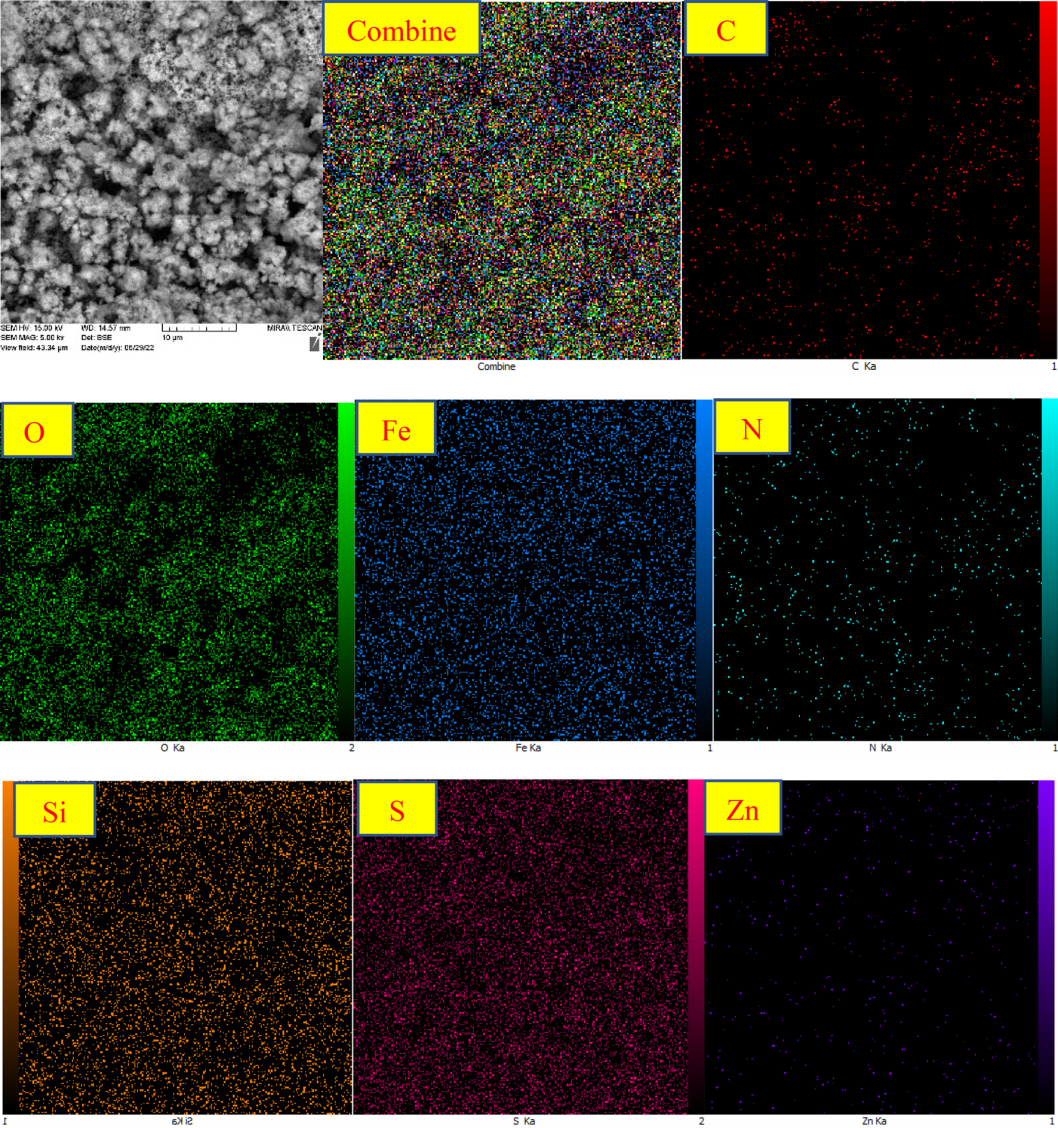
X-ray map spectrum of ZnFe_2_O_4_@SiO_2_@L-lysine@SO_3_H.

The X-ray mapping of ZnFe_2_O_4_@SiO_2_@L-lysine@SO_3_H shows the scattering of elements in the ZnFe_2_O_4_@SiO_2_@L-lysine@SO_3_H (Fig. 10). This analysis confirms the presence of Si, Fe, S, N, C, Zn, and O elements in the synthesized nanoparticle with a suitable and homogeneous dispersity throughout the ZnFe_2_O_4_ surface.

**Figure 11:**
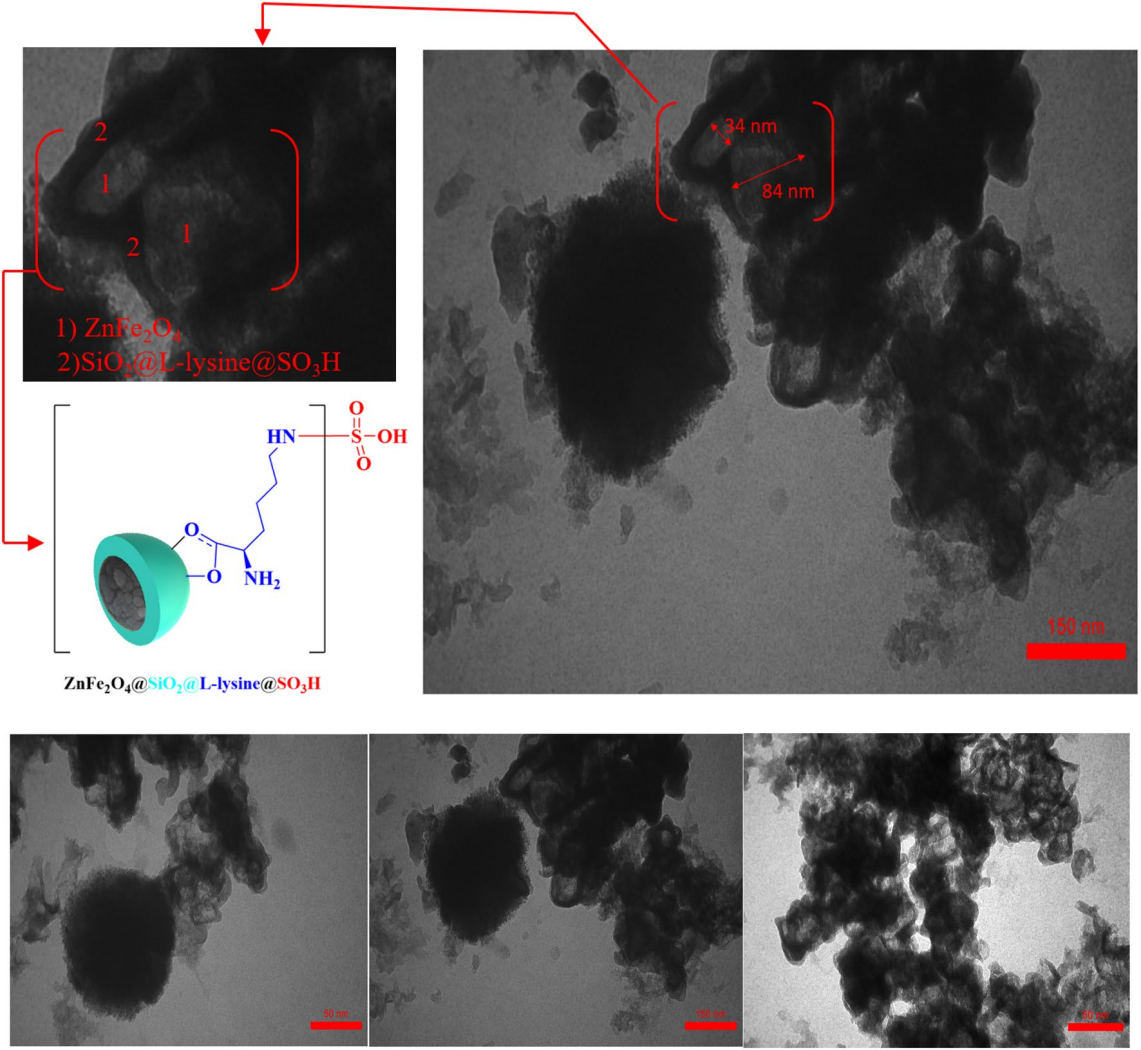
TEM micrograph of ZnFe_2_O_4_@SiO_2_@L-lysine@SO_3_H.

Using TEM images, the core–shell structure of ZnFe_2_O_4_@SiO_2_@L-lysine@SO_3_H cubic nanoparticles was investigated. From Fig. 11, we can see the cubic nanoparticles of the ZnFe_2_O_4_@SiO_2_@L-lysine@SO_3_H composites. The TEM micrograph showed agglomeration of many ultrafine cubic particles which display gray magnetite (ZnFe_2_O_4_) cores surrounded by a SiO_2_@L-lysine@SO_3_H shell. It is very interesting that the TEM image again verifies the yolk-shell microstructure in ZnFe_2_O_4_@SiO_2_@L-lysine@SO_3_H, and it is clear that dense silica layers and L-lysine@SO_3_H were formed around ZnFe_2_O_4_ nanocores (Fig. 11).

**Figure 12:**
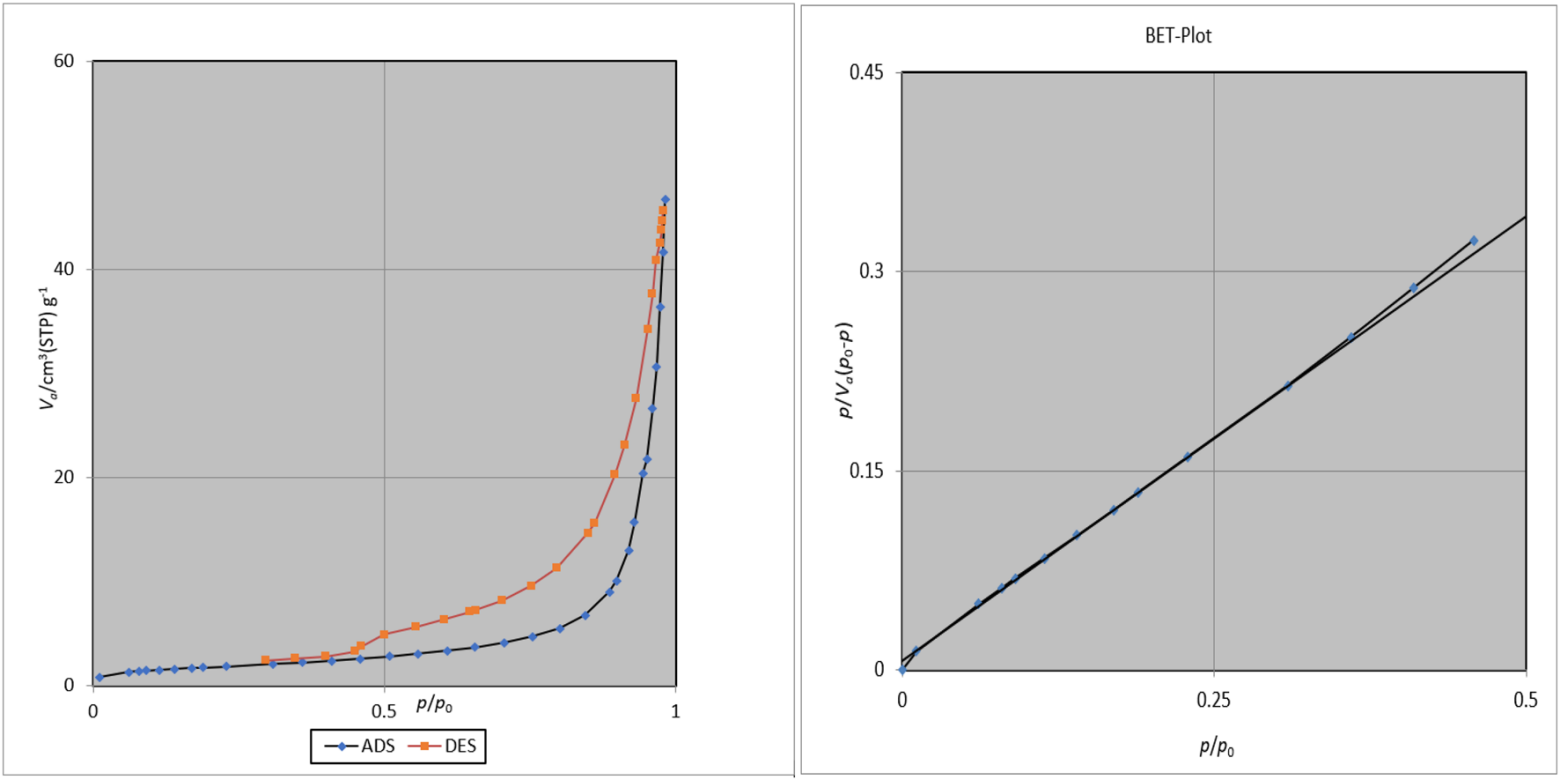
N_2_ adsorption/desorption isotherms, of the ZnFe_2_O_4_@SiO_2_@L-lysine@SO_3_H.

The surface area and size distribution of ZnFe_2_O_4_@SiO_2_@L-lysine@SO_3_H acid is studied by N_2_ adsorption–desorption isotherms analysis. Regarding the N_2_ adsorption–desorption isotherms technique, the obtained surface area of ZnFe_2_O_4_@SiO_2_@L-lysine@SO_3_H is 6.42 (m^2^/g) based on the BET method. Also, the total pore volume and average pore diametere of ZnFe_2_O_4_@SiO_2_@L-lysine@SO_3_H are obtained by the BET technique and the values are 0.07 cm^3^ g^−1^, and 44 nm, respectively (Fig. 12).

Using the back titration method, the acid strength of the synthesized catalyst, that is, the surface density of SO_3_H groups, was investigated and determined. First, 0.1 g of synthesized catalyst was added to the 50 mL water and stirred for 1 h, then 10 mL NaOH (0.1 N) was added to the mixture and was stirred as long as the pH did not change the as-synthesized catalyst was separated using an external magnet. Then, two drops of phenolphthalein were added to the mixture and were tittered with 1.9 mL HCl (0.1 N). Thus 1 g of catalyst has 8.1 mmol of the acidic groups.

**Figure 13:**
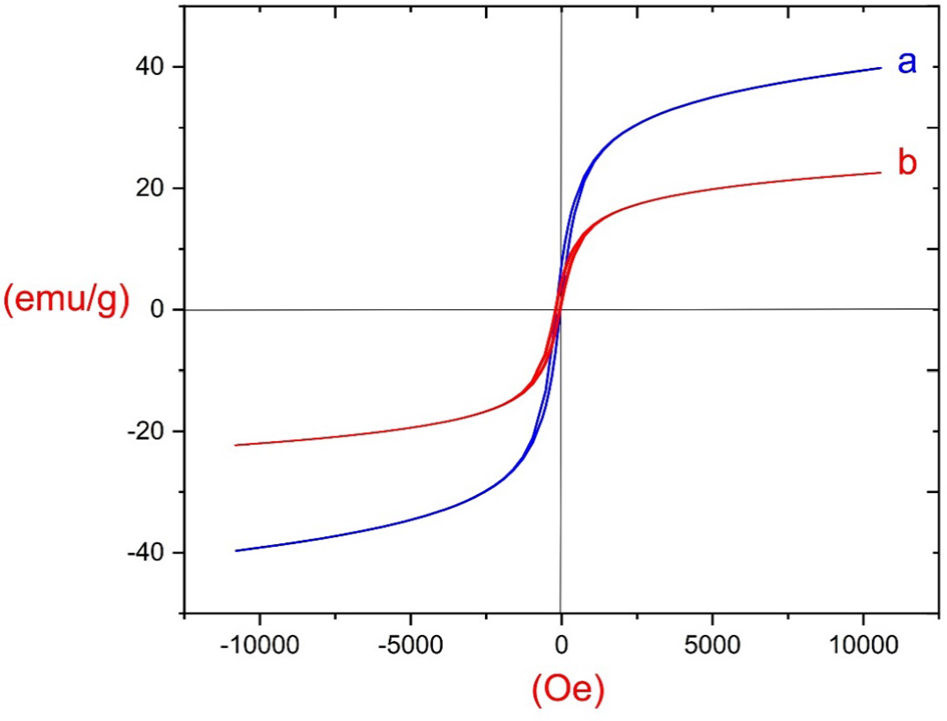
The magnetic properties of (**a**) ZnFe_2_O_4_ and (**b**) ZnFe_2_O_4_@SiO_2_@L-lysine@SO_3_H.

The magnetic behavior of ZnFe_2_O_4_ (a) and ZnFe_2_O_4_@SiO_2_@L-lysine@SO_3_H (b) composite was investigated with the vibrating sample magnetometer (VSM) (Fig. 13). The ZnFe_2_O_4_ nanoparticles exhibited almost zero coercivity and remanence with no hysteresis loop, approving the high permeability in magnetization and good magnetic responsiveness. Magnetic measurements showed saturation magnetization values of 41 and 22 emu/g for ZnFe_2_O_4_ and ZnFe_2_O_4_@SiO_2_@L-lysine@SO_3_H complex nanocomposite, respectively. The results showed that the magnetization of ZnFe_2_O_4_ decreases after the coating of L-lysine@SO_3_H on its surface, indicating the successful immobilization of the L-lysine@SO_3_H on ZnFe_2_O_4_.

### Catalytic study

#### Checking catalytic activity of ZnFe_2_O_4_@SiO_2_@L-lysine@SO_3_H for the synthesis of pyrazolyl

In the next step, after the successful synthesis and characterization of ZnFe_2_O_4_@SiO_2_@L-lysine@SO_3_H, its catalytic activity was considered for the synthesis of pyrazolyl derivatives and oxidation of sulfides.

In early research to obtain optimal reaction conditions, after structural characterization of the prepared nanocatalyst (ZnFe_2_O_4_@SiO_2_@L-lysine@SO_3_H), its catalytic activity was investigated in the synthesis of pyrazolyl (Table 1). The reaction between benzaldehyde (1 mmol), phenylhydrazine (2 mmol), and ethyl acetoacetate (2 mmol), was selected as the model reaction, and the influence of various parameters including amounts of catalyst, reaction temperature, and solvent were examined. The model reaction did not take place in the absence of the ZnFe_2_O_4_@SiO_2_@L-lysine@SO_3_H. After optimizing the catalyst’s amount, the effect of temperatures and several solvents was checked. The best results were obtained in solvent-free conditions using 0.03 g ZnFe_2_O_4_@SiO_2_@L-lysine@SO_3_H at 80 °C.

**Table 1:** Optimization of reaction conditions for the synthesis of 4,4'-(phenylmethylene)bis(3-methyl-1-phenyl-1H-pyrazol-5-ol) in the presence of ZnFe_2_O_4_@SiO_2_@L-lysine@SO_3_H as a catalyst.


Entry	The amount of catalyst(g)	Solvent	Temperature (degrees Celsius)	Time(min)	Yield
1	–	Solvent free	80	10 h	Trace
2	0.005	Solvent free	80	15	35
3	0.01	Solvent free	80	15	81
4	0.02	Solvent free	80	15	90
5	0.03	Solvent free	80	15	95
6	0.05	Solvent free	80	15	95
7	0.03	Ethanol	Reflux	15	83
8	0.03	H_2_O	80	15	71
9	0.03	EtOAc	Reflux	15	62
10	0.03	n-hexane	Reflux	15	62
11	0.03	Acetonitrile	Reflux	15	57
12	0.03	Solvent free	R. T	15	24
13	0.03	Solvent free	50	15	57
14	0.03	Solvent free	80	15	89
15	0.03^a^	Solvent free	80	15	Trace
16	0.03^b^	Solvent free	80	15	Trace
17	0.03^d^	Solvent free	80	15	Trace

^a^Reaction was performed in the presence of ZnFe_2_O_4_@SiO_2_.

^b^Reaction was performed in the presence of ZnFe_2_O_4_@SiO_2_@L-lysine.

^d^Reaction was performed in the presence of L-lysine.

After determining the optimal conditions, to identify the performance and generality of ZnFe_2_O_4_@SiO_2_@L-lysine@SO_3_H, the synthesis of diverse derivatives such as pyrazolyl was tested by various arylaldehydes (Table 2). As can be observed in this table, all arylaldehydes worked well in the reaction and it was observed that the synthesis of pyrazolyl in the presence of this catalyst afforded excellent yields with short reaction times.

**Table 2:** The one-pot of pyrazolyl catalyzed by ZnFe_2_O_4_@SiO_2_@L-lysine@SO_3_H.

Entry	Ar	Product	Tim(min)	Yield (%) ^a^	TON	TOF(h^-1^)	M.P. (°C)	
							Measured	Literature
1	C_6_H_5_		15	95	3.8	15	166–169	171–177^35^
2	3-ClC_6_H_4_		14	80	3.22	14	239–241	238–240^31^
3	4-ClC_6_H_4_		13	92	3.7	18	217–219	213–218^36^
4	3-BrC_6_H_4_		21	74	2.9	8.2	177–179	176–178^31^
5	4-MeC_6_H_4_		12	81	3.2	16	192–195	203–209^36^
6	4-MeOC_6_H_4_		20	80	3.2	10.6	177–179	173–179^36^
7	3-MeOC_6_H_4_		12	70	2.8	14	171–173	171–173^31^
8	2-MeOC_6_H_4_		14	71	2.9	13	177–179	175–178^37^
9	2,4-diClC_6_H_4_		22	77	3.1	8.6	223–225	222–225^37^
10	4-NO_2_C_6_H_4_		15	88	3.5	14	228–230	225–228^36^
11	3-NO_2_C_6_H_4_		15	91	3.6	14	140–143	151–154^36^
12	3-FC_6_H_4_		30	81	3.3	6.4	160–163	161–164^37^
13	4-OHC_6_H_4_		12	78	3.2	16	161–164	153–158^31^
14	3,4-di- MeOC_6_H_4_		45	77	3.1	4.1	180–183	191–193^36^
15	1H-indole-3-carbaldehyde		40	83	3.4	5.6	239–242	–
16	C_6_H_5_NO		25	97	3.9	9.5	201–203	201–204^38^
17	C_6_H_5_NO		20	73	2.9	10	233–235	232–235^35^

^a^Isolated yield.

**Figure 14:**
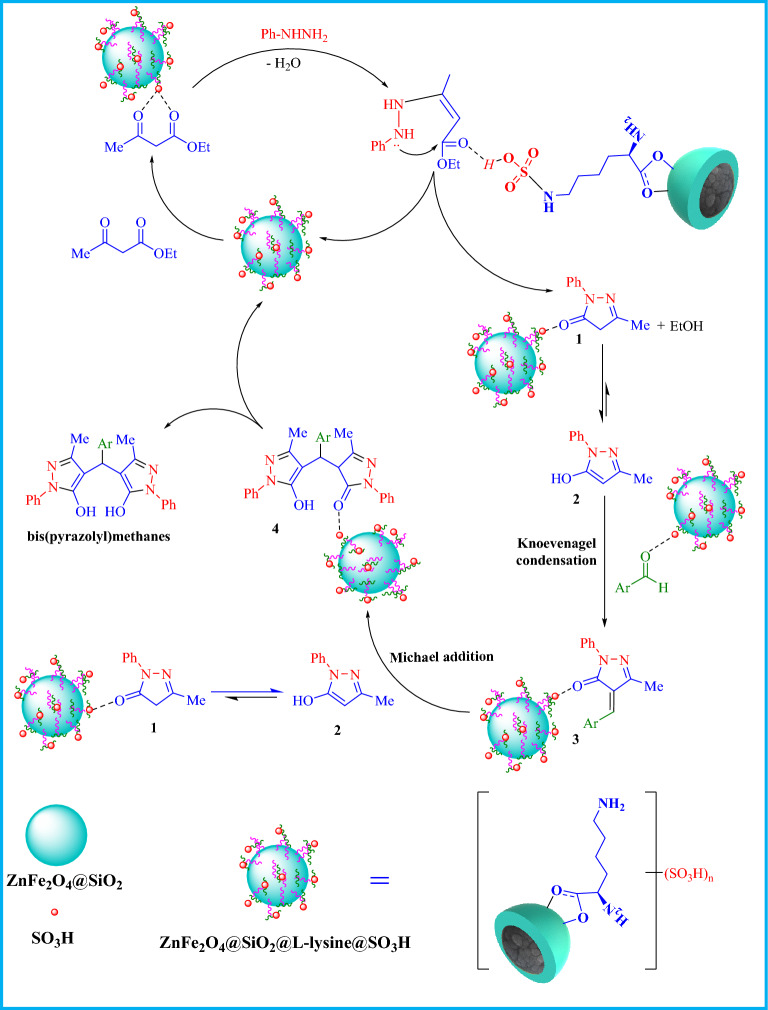
Proposed reaction mechanism.

The mechanism for the synthesis of pyrazolyl in the presence of ZnFe_2_O_4_@SiO_2_@L-lysine@SO_3_H has been depicted in Fig. 14. At the beginning of the reaction, ZnFe_2_O_4_@SiO_2_@L-lysine@SO_3_H composite activates C=O groups in the ethyl acetoacetate, and then phenylhydrazine attacks the C=O groups to afford pyrazolone 1 and was further rearranged into tautomer **2**. Next, a Knoevenagel-type reaction takes place between activated aldehydes and tautomer **2** followed by the liberation of an H_2_O molecule to form intermediate **3**. Then, a Michael addition reaction between intermediate **3** and tautomer **2** is facilitated to form intermediate **4**. In the final step, the corresponding products are formed by tautomerization and aromatization of intermediate **4**^35^.

After characterization of the synthesized heterogeneous ZnFe_2_O_4_@SiO_2_@L-lysine@SO_3_H nanocatalyst was examined in the oxidation of sulfide to understand the catalytic activity of the prepared material. First, the reaction of the Ph–S-Me with H_2_O_2_ was selected as a model reaction and carried out in the presence of ZnFe_2_O_4_@SiO_2_@L-lysine@SO_3_H Hat different conditions, including different temperatures and amounts of nanocatalyst, and the results showed that the catalyst showed high activity in solvent-free conditions at 25 °C at 120 min. In the next step, the effect of different solvents (EtOAc, n-Hexane, Ethanol, H_2_O) and also the conditions without solvent were investigated. It should be noted that in solvent-free conditions, the best yield was obtained in 120 min. The study of the amount of catalyst showed that the 0.03 g of nanocatalyst gave a high yield of product (Table 3, entries 1–5). The oxidation didn’t occur in the absence of ZnFe_2_O_4_@SiO_2_@L-lysine@SO_3_H even after 4 h (Table 3).

**Table 3:** Effects of various parameters on oxidation of sulfide in the presence of ZnFe_2_O_4_@SiO_2_@L-lysine@SO_3_H.


Entry	Catalyst (g)	Solvent	H_2_O_2_ (mg)	Time (min)	Yield (%)^a,b^
1	–	Solvent-free	0.3	4 h	N. R
2	0.01	Solvent-free	0.3	120	85
3	0.02	Solvent-free	0.3	120	87
4	0.03	Solvent-free	0.3	120	95
5	0.04	Solvent-free	0.3	120	95
6	0.03	n-Hexane	0.3	120	Trace
7	0.03	EtOH	0.3	120	54
8	0.03	H_2_O	0.3	120	Trace
9	0.03	EtOAc	0.3	120	20
10	0.03	Solvent-free	0.1	120	75
11	0.03	Solvent-free	0.2	120	90
12	0.03	Solvent-free	0.3	120	95
13	0.03	Solvent-free	0.4	120	95

^a^Reaction conditions: sulfide (1mmol) H_2_O_2_ (0.3 mL) and ZnFe_2_O_4_@SiO_2_@L-lysine@SO_3_H at 25 centigrade degrees under solvent-free conditions.

^b^Isolated yield.

In the next step, after the completion of optimization, the catalytic activity of ZnFe_2_O_4_@SiO_2_@L-lysine@SO_3_H in the oxidation of a wide range of sulfide derivatives was examined under the optimized conditions. It is necessary to mention that all sulfoxides were produced with high yields, which showed the excellent catalytic activity of the synthesized nanoparticles (Table 4). This catalytic system is a suitable method in terms of the efficiency of conditions.

**Table 4:** Oxidation of sulfides in the presence of ZnFe_2_O_4_@SiO_2_@L-lysine@SO_3_H.

Entry^a^	Substrate	Time (h)	Yield (%) ^b^	TON	TOF (h^−1^)	M.P. (°C)	
						Measured	Literature
1		2	95	3.8	1.9	31–35	30–34^39^
2		1.5	90	3.6	2.4	129–133	129–132^39^
3		2	81	3.2	1.6	Oil	–
4		3	92	3.7	1.2	113–115	112–115^30^
5		1.5	89	3.5	2.3	Oil	–
6		2.5	85	3.4	1.3	Oil	–

^a^Isolated yield.

^b^Reaction conditions: sulfide (1 mmol) H_2_O_2_ (0.3 mL) and ZnFe_2_O_4_@SiO_2_@L-lysine@SO_3_H under solvent-free conditions at room temperature.

**Figure 15:**
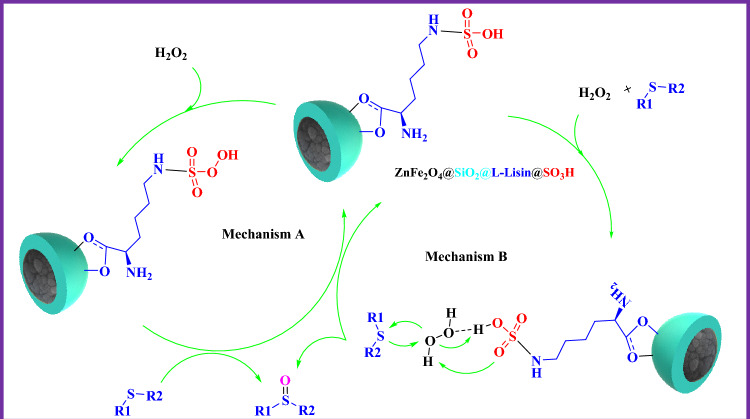
Possible mechanism for the oxidation of sulfide.

The proposed mechanism for the oxidation of sulfide to the corresponding sulfoxide is shown in Fig. 15. The efficiency of the oxidation can be explained by the interaction between the ZnFe_2_O_4_@SiO_2_@L-lysine@SO_3_H and H_2_O_2_. The OH moiety of the ZnFe_2_O_4_@SiO_2_@L-lysine@SO_3_H forms a strong hydrogen bond with H_2_O_2_ and increases the electrophilic ability of a peroxy oxygen atom of H_2_O_2_. In these reaction conditions hydrogen bonding may be assisting in controlling the chemoselectivity, because the hydrogen bond between the catalyst and the oxygen of the sulfoxides could decrease the nucleophilicity of the sulfur atom of the sulfoxides and prevent further oxidation of the sulfoxides. One explanation for this transformation is the in-situ formation of peroxy acid using the reaction of ZnFe_2_O_4_@SiO_2_@L-lysine@SO_3_H with Hydrogen peroxide, followed by the oxygen transfer to the organic substrate (Fig. 15a). Another explanation is that ZnFe_2_O_4_@SiO_2_@L-lysine@SO_3_H acts as protic acid, which polarizes the oxygen–oxygen bond in hydrogen peroxide to produce the reactive oxygen transfer agent (Fig. 15b)^40^.

## Hot filtration

In this part, with optimal reaction conditions in hand, to confirm the heterogeneous nature of the ZnFe_2_O_4_@SiO_2_@L-lysine@SO_3_H in the synthesis of pyrazolyl compounds hot filtration experiment was performed using benzaldehyde as a model reaction. At the half time of reaction, the corresponding product was obtained in 55% of the yield. Next, when the reaction mixture was run in another half-time in the absence of nanocatalyst, the reaction afforded no augmentation in its yield. It can be concluded from this point that the catalyst can be considered a true heterogeneous nanocatalyst. Moreover, the stability of the L-lysine@SO_3_H complex on the surface of ZnFe_2_O_4_ confirms the heterogeneous nature of the as-prepared nanocatalyst.

## Reusability of ZnFe_2_O_4_@SiO_2_@L-lysine@SO_3_H

**Figure 16:**
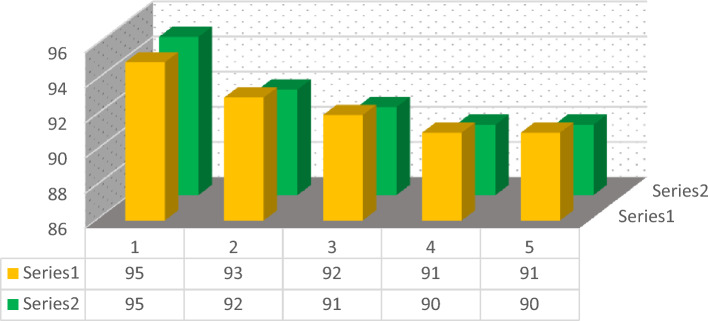
Recyclability of ZnFe_2_O_4_@SiO_2_@L-lysine@SO_3_H for the preparation of oxidation of methyl phenyl sulfide (series 1) and 4,4'-(phenylmethylene)bis(pyrazole) (series 2).

The recoverability of ZnFe_2_O_4_@SiO_2_@L-lysine@SO_3_H catalyst was investigated for oxidation of sulfides (series 1) and Synthesis of pyrazolyl (series 2) derivatives. In this study, the recovery of the nanocatalyst from the reaction mixture was successfully carried out, which could be easily separated with a neodymium magnet and washed several times with EtOAc and DI (H_2_O). Then the recovered nanocatalyst was used in the next run. The results showed that recycled catalysts can be employed at both of the reactions up to five times, with insignificant loss of catalyst activity (Fig. 16).

### Comparison of the catalyst

The comparative study of different catalytic for the synthesis of pyrazolyl derivatives (Table 5), with several previously reported methods, is presented. In the present research, the products were obtained in higher yields over faster times in the presence of ZnFe_2_O_4_@SiO_2_@L-lysine@SO_3_H. In addition, this catalyst is environmentally friendly and has several advantages in terms of sustainability, price, separation, and non-toxicity.

**Table 5:** Comparison results of ZnFe_2_O_4_@SiO_2_@L-lysine@SO_3_H with other catalysts in the synthesis of pyrazolyl.

Entry	Reaction product	Catalyst	Time (min)	Yield (%)	Ref
1	4,4'-((4-chlorophenyl)methylene)bis(3-methyl-1-phenyl-1H-pyrazol-5-ol)	SBA-15@Tromethamine-Pr	25	95	^35^
2	4,4'-((4-chlorophenyl)methylene)bis(3-methyl-1-phenyl-1H-pyrazol-5-ol)	SASPSPE	132	85	^41^
3	4,4'-((4-chlorophenyl)methylene)bis(3-methyl-1-phenyl-1H-pyrazol-5-ol)	DCDBTSD	40	80	^42^
4	4,4'-((4-chlorophenyl)methylene)bis(3-methyl-1-phenyl-1H-pyrazol-5-ol)	ZnFe_2_O_4_@SiO_2_@L-lysine@SO_3_H	15	92	This work

## Conclusions

In this research project, we have successfully synthesized ZnFe_2_O_4_@SiO_2_@L-lysine@SO_3_H nanoparticles as an effective and recoverable nanocatalyst. Wide active surface area, reusability, suitable stability, excellent heterogeneity, and substantial magnetic behavior have distinguished this catalytic system as an instrumental tool for the synthesis of organic compounds. This research reported a novel route for the synthesis of an extensive range of synthesis of pyrazolyl derivatives and oxidation of sulfides with high yields and purity. The wondrous features of this protocol are novelty, no use of harmful solvents, simple synthesis procedure, short reaction time, facile filtration, and reusability. In addition, the as-synthesized magnetic nanocatalyst could be separated easily using a external magnet and reused several times without significant loss of its catalytic activity.

### Supplementary Information

Supplementary Figures.

## Data Availability

All data generated or analyzed during this study are included in this published article [and its Supplementary Information Files].

## References

[1] Nguyen NTT, Nguyen TTT, Nguyen DTC, Tran TV (2023). Green synthesis of ZnFe_2_O_4_ nanoparticles using plant extracts and their applications: A review. Sci. Total Environ..

[2] Ragu S, Kim B, Chen S-M, Ishfaque A, Kang K-M (2023). N-substituted CQDs impregnated by Fe_3_O_4_ heterostructure: Bifunctional catalyst for electro-catalytic and photo-catalytic detection of an environmental hazardous organic pollutant. Chemosphere.

[3] Han Q (2023). Polyethylene glycol functionalized Fe_3_O_4_@MIL-101(Cr) for the efficient removal of heavy metals from Ligusticum chuanxiong Hort. Arab. J. Chem..

[4] Chen M-N, Mo L-P, Cui Z-S, Zhang Z-H (2019). Magnetic nanocatalysts: Synthesis and application in multicomponent reactions. Curr. Opin. Green Sustain. Chem..

[5] Zhang M, Liu Y-H, Shang Z-R, Hu H-C, Zhang Z-H (2017). Supported molybdenum on graphene oxide/Fe_3_O_4_: An efficient, magnetically separable catalyst for one-pot construction of spiro-oxindole dihydropyridines in deep eutectic solvent under microwave irradiation. Catal. Commun..

[6] Ghasemzadeh MA, Ghaffarian F (2020). Preparation of core/shell/shell CoFe_2_O_4_/OCMC/Cu (BDC) nanostructure as a magnetically heterogeneous catalyst for the synthesis of substituted xanthenes, quinazolines and acridines under ultrasonic irradiation. Appl. Organomet. Chem..

[7] Poonia K (2023). Recent advances in metal organic framework (MOF)-based hierarchical composites for water treatment by adsorptional photocatalysis: A review. Environ. Res..

[8] Zhang H-Y (2017). A magnetic metal–organic framework as a highly active heterogeneous catalyst for one-pot synthesis of 2-substituted alkyl and aryl(indolyl)kojic acid derivatives. N. J. Chem..

[9] Gao G, Di J-Q, Zhang H-Y, Mo L-P, Zhang Z-H (2020). A magnetic metal organic framework material as a highly efficient and recyclable catalyst for synthesis of cyclohexenone derivatives. J. Catal..

[10] Huang X (2023). Space-confined growth of nanoscale metal-organic frameworks/Pd in hollow mesoporous silica for highly efficient catalytic reduction of 4-nitrophenol. J. Colloid Interface Sci..

[11] Cai W, Zhang W, Chen Z (2023). Magnetic Fe_3_O_4_@ZIF-8 nanoparticles as a drug release vehicle: pH-sensitive release of norfloxacin and its antibacterial activity. Colloids Surf. B Biointerfaces.

[12] Öztürk D, Mıhçıokur H (2023). Removal of lansoprazole one of the most prescribed drugs in Turkey from an aqueous solution by innovative magnetic nanomaterial Tween 85®PEI@Fe_3_O_4_. J. Water Process Eng..

[13] Al-husseiny RA, Kareem SL, Naje AS, Ebrahim SE (2023). Effect of green synthesis of Fe_3_O_4_ nanomaterial on the removal of cefixime from aqueous solution. Biomass Convers. Biorefinery.

[14] Jinxi W (2023). Tailoring of ZnFe_2_O_4_-ZrO_2_-based nanoarchitectures catalyst for supercapacitor electrode material and methanol oxidation reaction. Fuel.

[15] Doiphode V (2021). Solution-processed electrochemical synthesis of ZnFe_2_O_4_ photoanode for photoelectrochemical water splitting. J. Solid State Electrochem..

[16] Agyemang FO, Kim H (2016). Electrospun ZnFe_2_O_4_-based nanofiber composites with enhanced supercapacitive properties. Mater. Sci. Eng. B Solid-State Mater. Adv. Technol..

[17] Zhang X (2023). ZnFe_2_O_4_ nanospheres decorated residual carbon from coal gasification fine slag as an ultra-thin microwave absorber. Fuel.

[18] Sanko V, Şenocak A, Tümay SO, Demirbas E (2023). A novel comparative study for electrochemical urea biosensor design: Effect of different ferrite nanoparticles (MFe_2_O_4_, M: Cu Co, Ni, Zn) in urease immobilized composite system. Bioelectrochemistry.

[19] Gandomi F (2023). ROS, pH, and magnetically responsive ZnFe_2_O_4_@l-Cysteine@NGQDs nanocarriers as charge-reversal drug delivery system for controlled and targeted cancer chemo-sonodynamic therapy. Inorg. Chem. Commun..

[20] Marandi A, Kolvari E, Gilandoust M, Zolfigol MA (2022). Immobilization of –OSO_3_H on activated carbon powder and its use as a heterogeneous catalyst in the synthesis of phthalazine and quinoline derivatives. Diamond Relat. Mater..

[21] Malysheva S (2023). Phosphine chalcogenides and their derivatives from red phosphorus and functionalized pyridines, imidazoles, pyrazoles and their antimicrobial and cytostatic activity. Bioorgan. Chem..

[22] Ayman R, Abusaif MS, Radwan AM, Elmetwally AM, Ragab A (2023). Development of novel pyrazole, imidazo[1,2-b]pyrazole, and pyrazolo[1,5-a]pyrimidine derivatives as a new class of COX-2 inhibitors with immunomodulatory potential. Eur. J. Med. Chem..

[23] Roney M (2023). Identification of pyrazole derivatives of usnic acid as novel inhibitor of SARS-CoV-2 main protease through virtual screening approaches. Mol. Biotechnol..

[24] Wang J-L (2023). Four unprecedented V14 clusters as highly efficient heterogeneous catalyst for CO_2_ fixation with epoxides and oxidation of sulfides. Sci. China Chem..

[25] Zhang J (2023). Preparation of core/shell-structured ZnFe_2_O_4_@ZnIn_2_S_4_ catalysts and its ultrafast microwave catalytic reduction performance for aqueous Cr(VI). Chem. Eng. J..

[26] Sadeghi Z, Hajiarab R (2023). New nanoparticles of NaY, Ni-NaY, and Mn-NaY zeolites: Highly efficient catalysts for the oxidation of sulfides to sulfoxides. Phosphorus Sulfur Silicon Relat. Elem..

[27] Wan WL (2023). Samarium oxide as efficient and non-endangered metal for synthesis of sulfones from sulfides: An elemental sustainability concept. J. Taibah Univ. Sci..

[28] Khanmohammadi-Sarabi F, Ghorbani-Choghamarani A, Aghavandi H, Zolfigol MA (2023). l-Methionine-Zr complex supported on magnetic ZnFe_2_O_4_ as a novel, green, and efficient heterogeneous magnetic nanocatalyst for the synthesis of 1H-tetrazole and polyhydroquinoline derivatives. N. J. Chem..

[29] Khanmohammadi-Sarabi F, Ghorbani-Choghamarani A, Aghavandi H, Zolfigol MA (2022). ZnFe_2_O_4_@SiO_2_-ascorbic acid: Green, magnetic, and versatile catalyst for the synthesis of chromeno[2,3-d] pyrimidine-8-amine and quinazoline derivatives. Appl. Organomet. Chem..

[30] Aghavandi H, Ghorbani-Choghamarani A (2022). ZnFe_2_O_4_@l-Arginine-Ni: A novel, green, recyclable, and highly versatile catalyst for the synthesis of 1H-tetrazoles and oxidation of sulfides to the sulfoxides. J. Phys. Chem. Solids.

[31] Ghorbani-Choghamarani A, Aghavandi H, Talebi SM (2022). A new copper-supported zinc ferrite as a heterogeneous magnetic nanocatalyst for the synthesis of bis(pyrazolyl)methanes and oxidation of sulfides. Sci. Rep..

[32] Aghavandi H, Ghorbani-Choghamarani A (2022). Preparation and application of ZnFe_2_O_4_@SiO_2_–SO_3_H, as a novel heterogeneous acidic magnetic nanocatalyst for the synthesis of tetrahydrobenzo[b]pyran and 2,3-dihydroquinazolin-4(1H)-one derivative. Res. Chem. Intermed..

[33] Andhare DD (2020). Structural and chemical properties of ZnFe_2_O_4_ nanoparticles synthesised by chemical co-precipitation technique. J. Phys. Conf. Ser..

[34] Mohammadi M, Ghorbani-choghamarani A (2022). Hercynite silica sulfuric acid : a novel inorganic sulfurous solid acid catalyst for one-pot cascade organic transformations. RSC Adv..

[35] Aghavandi H, Ghorbani-Choghamarani A, Mohammadi M (2022). Mesoporous SBA-15@Tromethamine-Pr: Synthesis, characterization and its catalytic application in the synthesis of Bis(Pyrazolyl)Methanes. Polycycl. Aromat. Compd..

[36] Filian H, Kohzadian A, Mohammadi M, Ghorbani-Choghamarani A, Karami A (2020). Pd(0)-guanidine@MCM-41: A very effective catalyst for rapid production of bis (pyrazolyl)methanes. Appl. Organomet. Chem..

[37] Anizadeh MR, Torabi M, Zolfigol MA, Yarie M (2023). Catalytic application Fe_3_O_4_@SiO_2_@(CH_2_)_3_-urea-dithiocarbamic acid for the synthesis of triazole-linked pyridone derivatives. J. Mol. Struct..

[38] Kordnezhadian R (2020). Polyethylene glycol-bonded triethylammonium l-prolinate: A new biodegradable amino-acid-based ionic liquid for the one-pot synthesis of bis(pyrazolyl)methanes as DNA binding agents. N. J. Chem..

[39] Molaei S, Ghadermazi M (2021). A green methodology for thioether formation reaction and synthesis of symmetrical disulfides over new heterogeneous Cu attached to bifunctionalized mesoporous MCM-41. Microporous Mesoporous Mater..

[40] Mirfakhraei S, Hekmati M, Eshbala FH, Veisi H (2018). Fe_3_O_4_/PEG-SO_3_H as a heterogeneous and magnetically-recyclable nanocatalyst for the oxidation of sulfides to sulfones or sulfoxides. N. J. Chem..

[41] Tayebi S, Baghernejad M, Saberi D, Niknam K (2011). Sulfuric Acid ([3-(3-Silicapropyl)sulfanyl]propyl)ester as a Recyclable Catalyst for the Synthesis of 4,4′-(Arylmethylene)bis(1H-pyrazol-5-ols). Chin. J. Catal..

[42] Khazaei A, Abbasi F, Moosavi-Zare AR (2014). Tandem cyclocondensation-Knoevenagel-Michael reaction of phenyl hydrazine, acetoacetate derivatives and arylaldehydes. N. J. Chem..

