# Supplementary material for: ZnFe2O4@SiO2@L-lysine@SO3H: preparation, characterization, and its catalytic applications in the oxidation of sulfides and synthesis of Bis(pyrazolyl)methanes
Source: Sci Rep. 2024 Mar 28;14:7449. doi: 10.1038/s41598-024-57317-2 (PMC10978937; doi:10.1038/s41598-024-57317-2)
Supplement: Supplementary file 1 — Supplementary Figures. [file 41598_2024_57317_MOESM1_ESM.pdf]

$ZnFe_2O_4$@SiO_2@L-lysine@SO_3H: preparation, characterization, and its catalytic applications in the oxidation of sulfides and Synthesis of Bis(pyrazolyl)methanes

Amir Ghanbarpour, Arash Ghorbani-Choghamarani*, Hamid Aghavandi, Ahmad Jafari

Department of Organic Chemistry, Faculty of Chemistry and Petroleum Sciences, Bu-Ali Sina University, Hamedan, 6517838683, Iran.

*E-mail: a.ghorbani@basu.ac.ir

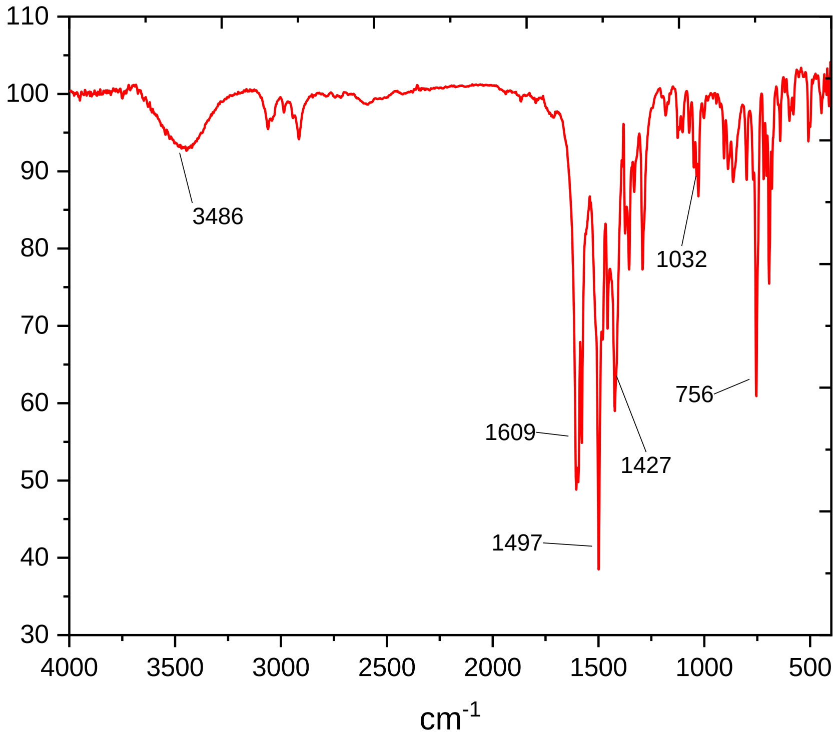

Supplementary figure 1: 4,4'-(pyridin-3-ylmethylene)bis(3-methyl-1-phenyl-1H-pyrazol-5-ol)

4,4'-(pyridin-3-ylmethylene)bis(3-methyl-1-phenyl-1H-pyrazol-5-ol): FT-IR (KBr) cm^{-1}: 756, 1032, 1427, 1497, 1609, 3486.

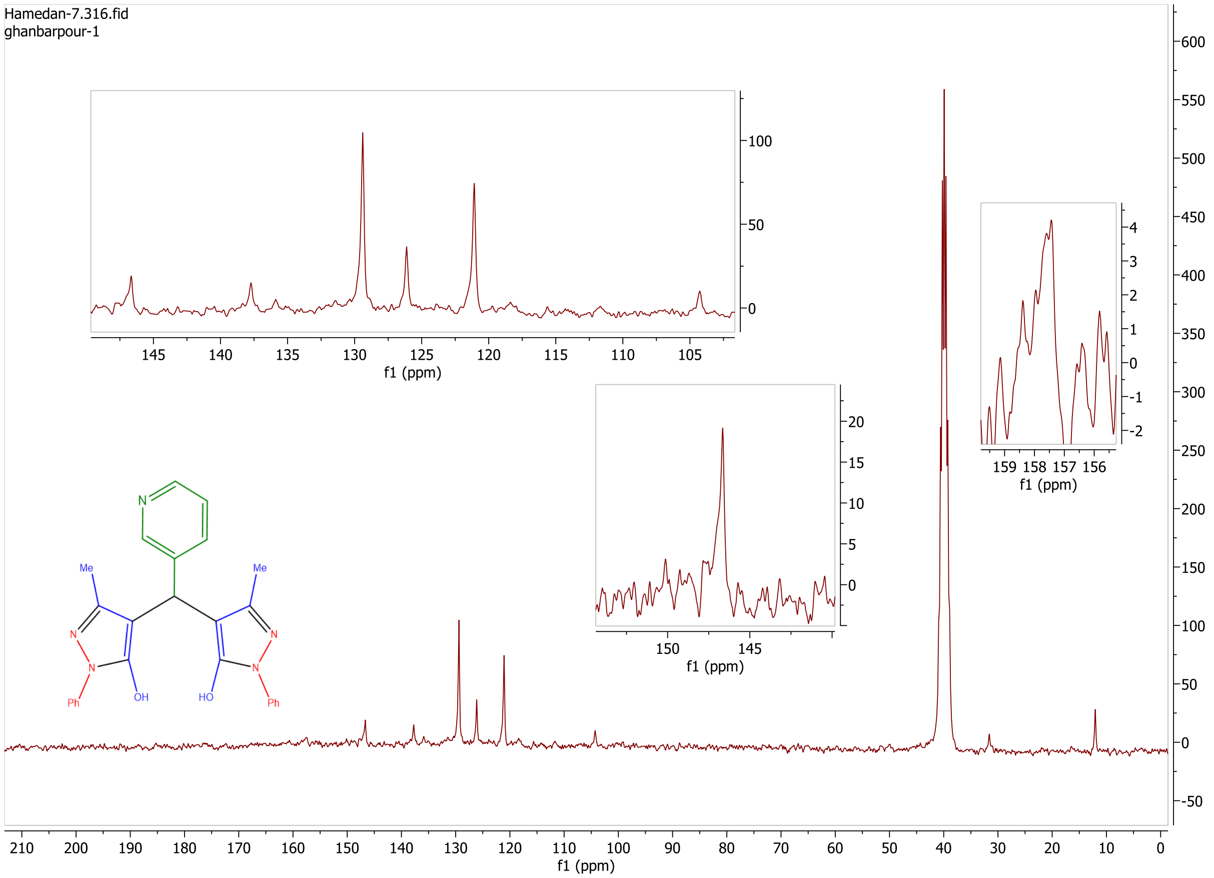

Supplementary figure 2: 4,4'-(pyridin-3-ylmethylene)bis(3-methyl-1-phenyl-1H-pyrazol-5-ol)

^{13}C NMR (62.5 MHz, DMSO): 11.9, 33.6, 104.5, 118.6, 120,3, 125.5, 129.5, 135.2, 137.5, 139.1, 142.5, 147.5, 155.8, 156.1, 158.2 ppm.

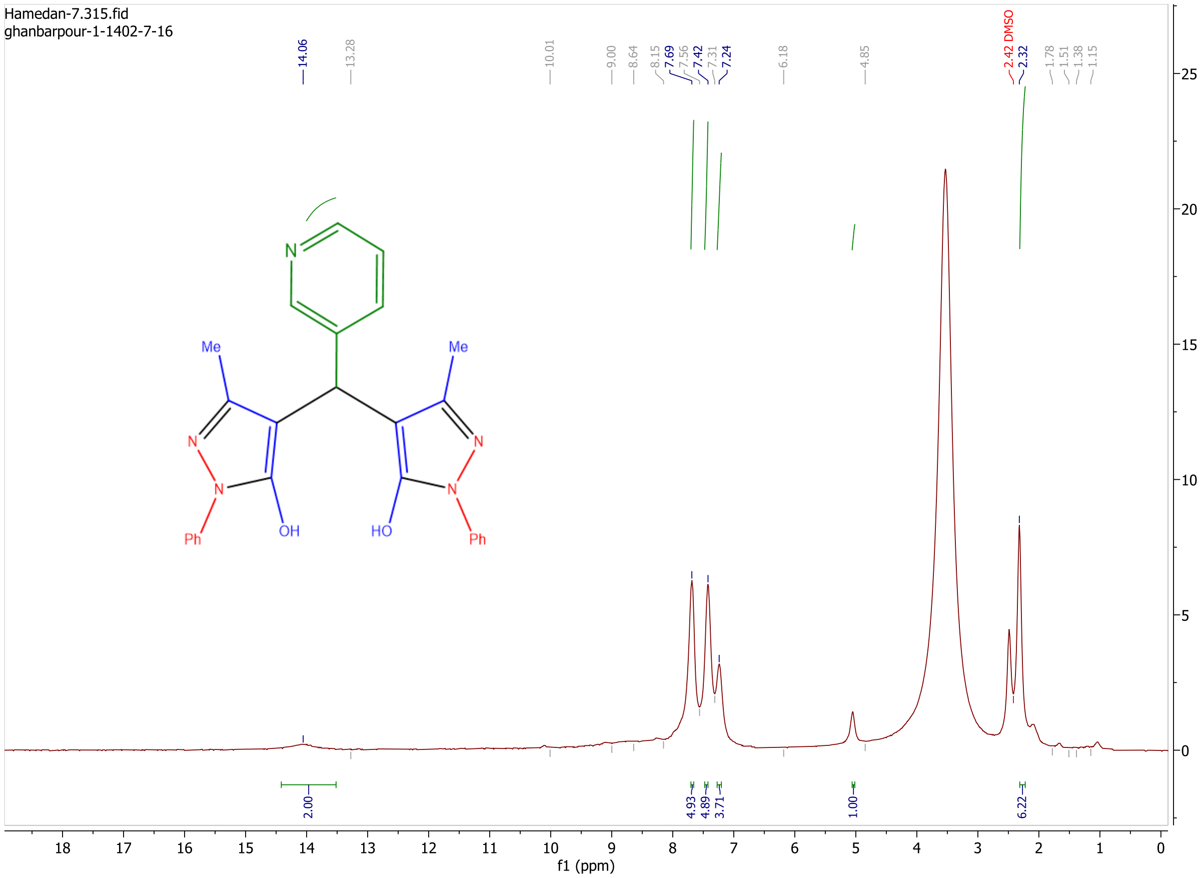

Supplementary figure 3: 4,4'-(pyridin-3-ylmethylene)bis(3-methyl-1-phenyl-1H-pyrazol-5-ol)

4,4'-(pyridin-3-ylmethylene)bis(3-methyl-1-phenyl-1H-pyrazol-5-ol): ^{1}H NMR (250 MHz, DMSO): 2.32 (m, 6H), 4.85 (s, 1H), 6.24-7. 69 (m, 14H), 14.06 (s, br, 2H) ppm.

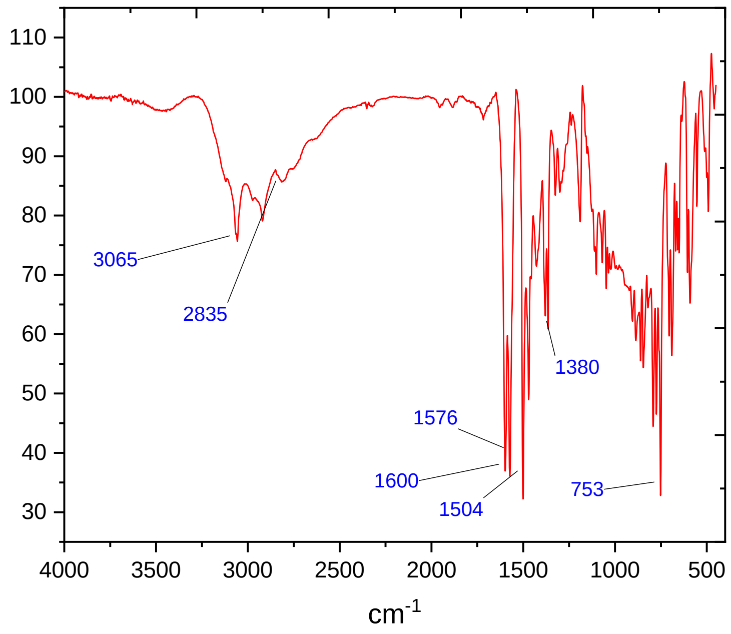

Supplementary figure 4: 4,4'-(thiophen-2-ylmethylene)bis(3-methyl-1-phenyl-1H-pyrazol-5-ol)

4,4'-(thiophen-2-ylmethylene)bis(3-methyl-1-phenyl-1H-pyrazol-5-ol): FT-IR (KBr) cm^{-1}: 753, 1380, 1504, 1575, 1600, 2835, 3065.

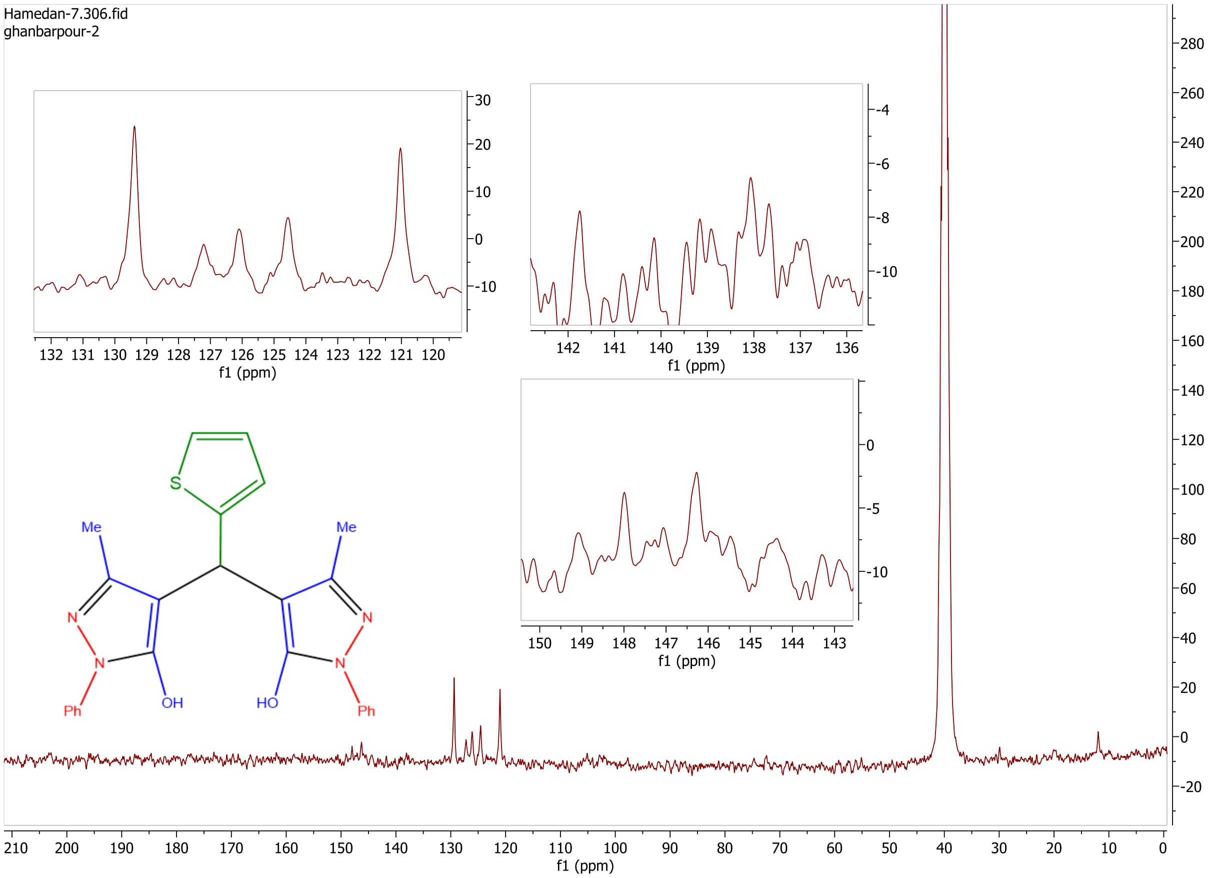

Supplementary figure 5: 4,4'-(thiophen-2-ylmethylene)bis(3-methyl-1-phenyl-1H-pyrazol-5-ol)

^{13}C NMR (62.5 MHz, DMSO): 10.8, 34.6, 121.0, 124.1, 124.9, 126.6, 127.1, 128.1, 128.3, 129.2, 138.0, 142.5, 146.1, 149.0 ppm.

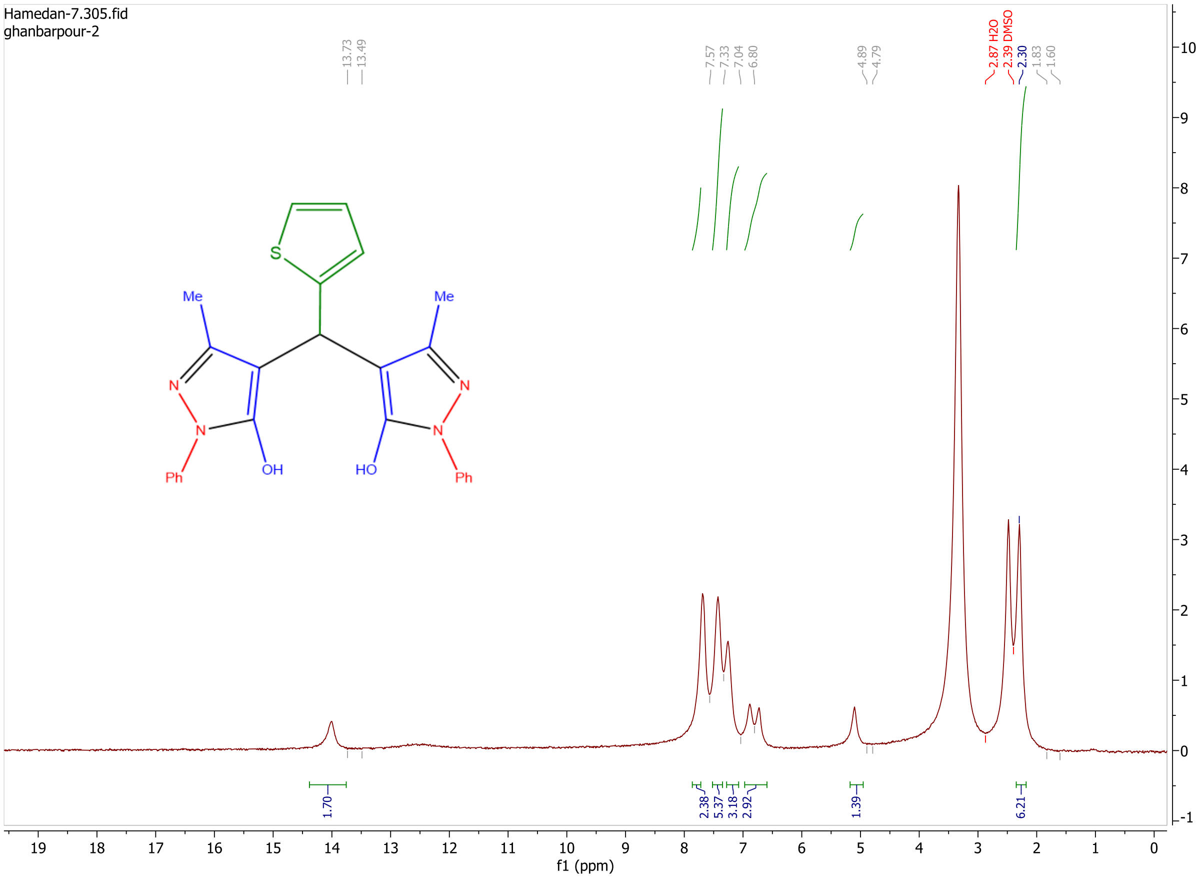

Supplementary figure 6: 4,4'-(thiophen-2-ylmethylene)bis(3-methyl-1-phenyl-1H-pyrazol-5-ol)

4,4'-(thiophen-2-ylmethylene)bis(3-methyl-1-phenyl-1H-pyrazol-5-ol): ^{1}H NMR (250 MHz, DMSO): 2.30 (m, 6H), 4.89 (s, 1H), 6.80-7. 75 (m, 13H), 14.06 (s, br, 2H) ppm.

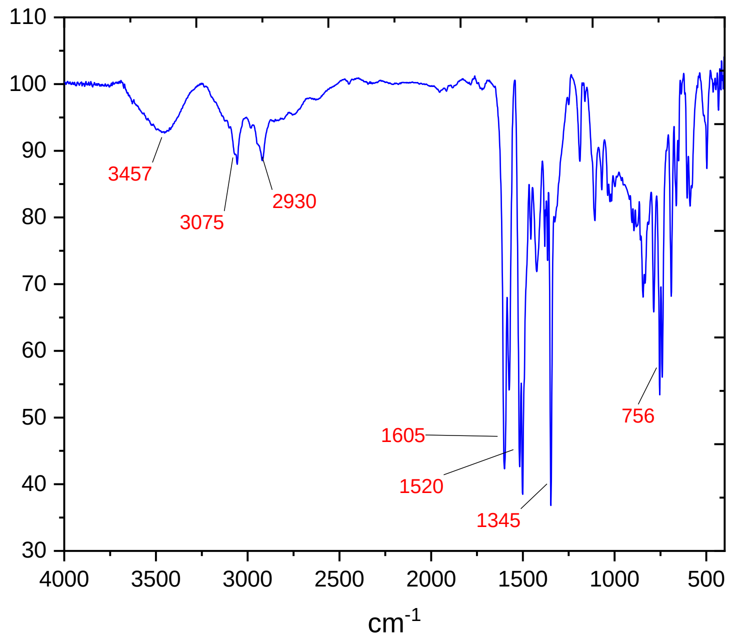

Supplementary figure 7: 4,4'-((4-nitrophenyl)methylene)bis(3-methyl-1-phenyl-1H-pyrazol-5-ol)

4,4'-((4-nitrophenyl)methylene)bis(3-methyl-1-phenyl-1H-pyrazol-5-ol): FT-IR (KBr) cm^{-1}: 756, 1345, 1520, 1605, 2930,3075, 3475.

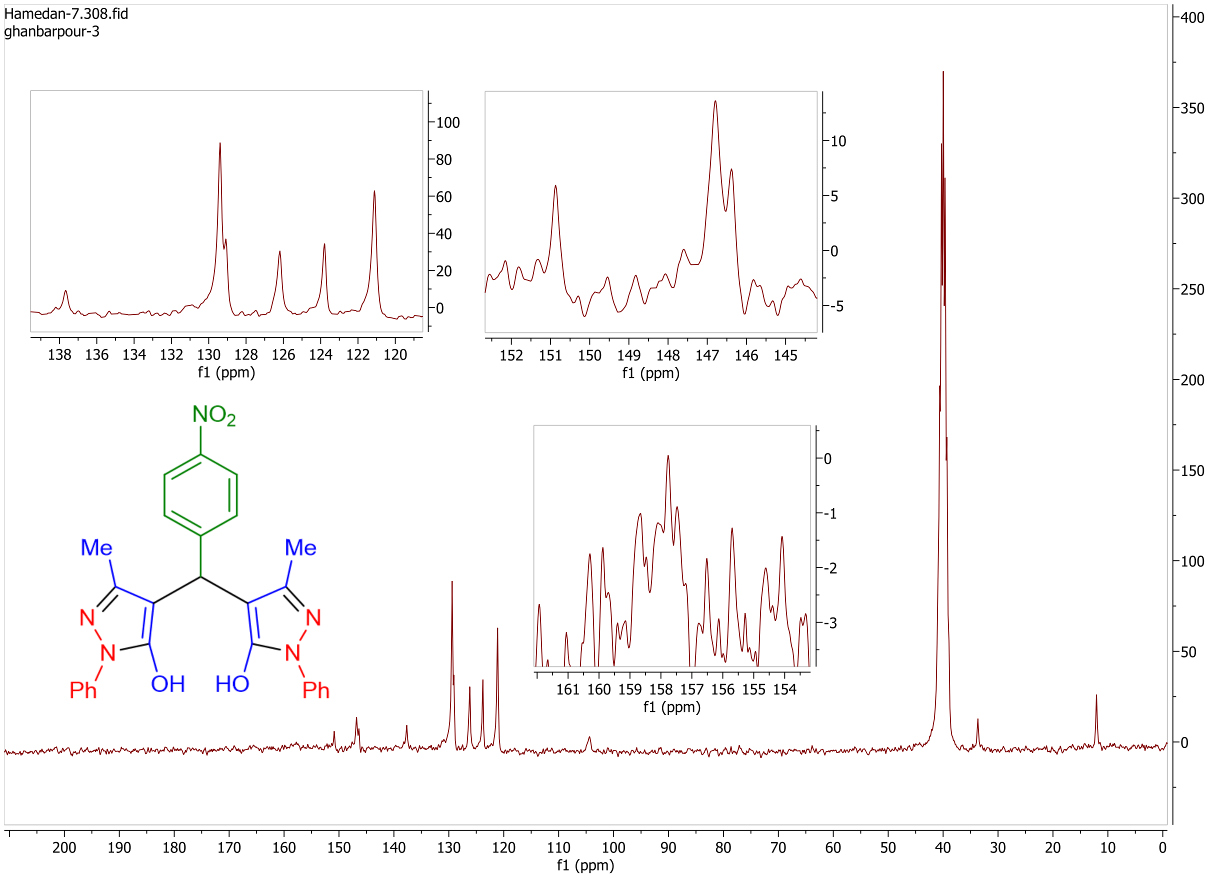

Supplementary figure 8: 4,4'-((4-nitrophenyl)methylene)bis(3-methyl-1-phenyl-1H-pyrazol-5-ol)

^{13}C NMR (62.5 MHz, DMSO): 11.0, 35.4, 120.4, 123.3, 126.2, 128.9, 130.0, 138.2, 146.2, 147.0, 150.7 154.8, 155.4, 158.3, 159.9 ppm.

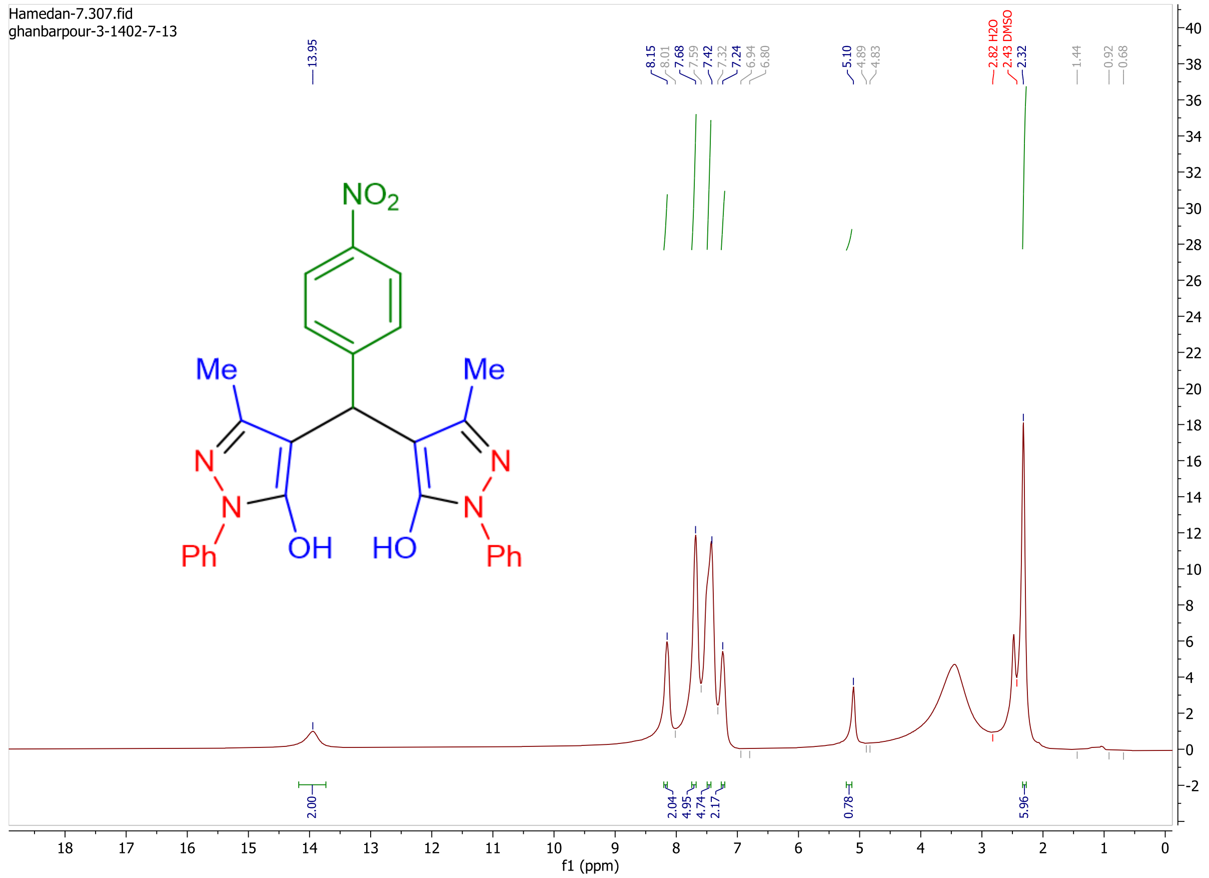

Supplementary figure 9: 4,4'-((4-nitrophenyl)methylene)bis(3-methyl-1-phenyl-1H-pyrazol-5-ol)

4,4'-((4-nitrophenyl)methylene)bis(3-methyl-1-phenyl-1H-pyrazol-5-ol): ^{1}H NMR (250 MHz, DMSO): 2.32 (m, 6H), 5.10 (s, 1H), 6.80-8.15 (m, 14H), 13.95 (s, br, 2H) ppm.

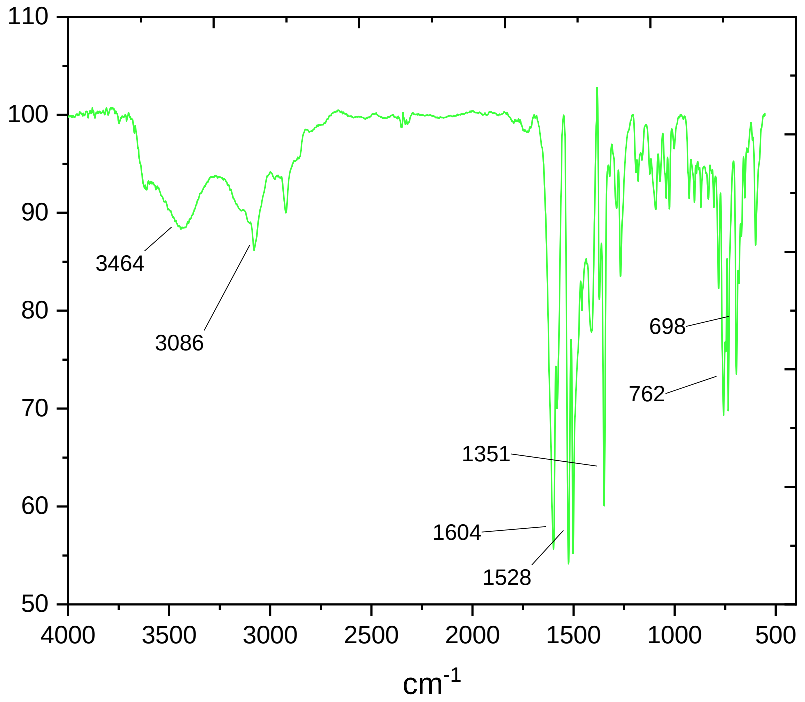

Supplementary figure 10: 4,4'-((3-nitrophenyl)methylene)bis(3-methyl-1-phenyl-1H-pyrazol-5-ol

4,4'-((3-nitrophenyl)methylene)bis(3-methyl-1-phenyl-1H-pyrazol-5-ol: FT-IR (KBr) cm^{-1}: 698, 762, 1351, 1528, 1604, 3086, 3464.

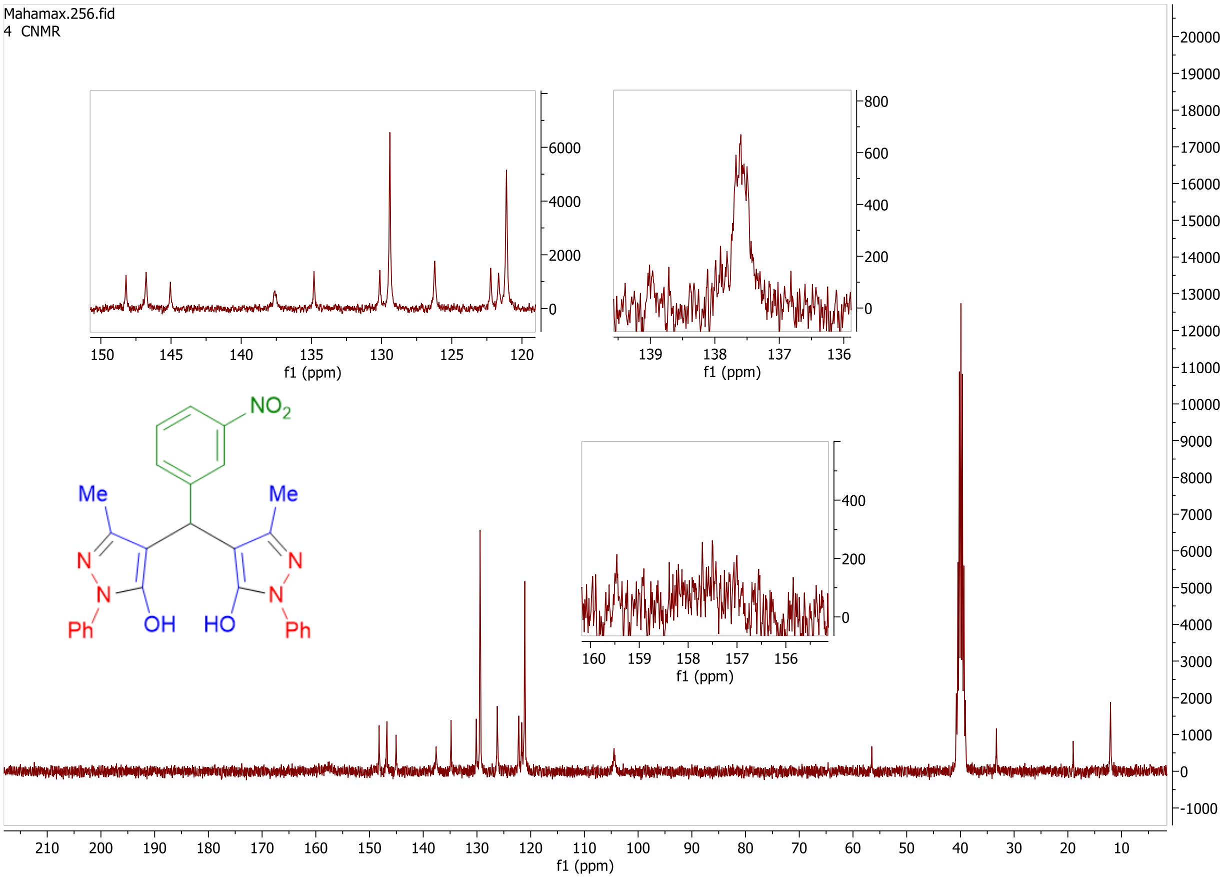

Supplementary figure 11: 4,4'-((3-nitrophenyl)methylene)bis(3-methyl-1-phenyl-1H-pyrazol-5-ol

^{13}C NMR (62.5 MHz, DMSO): 10.5, 34.1, 120.5, 120.9, 122.4, 126.7, 129.7,137.3, 137.6 137.9, 139, 145.6, 147.8, 148.3, 157.4 ppm.

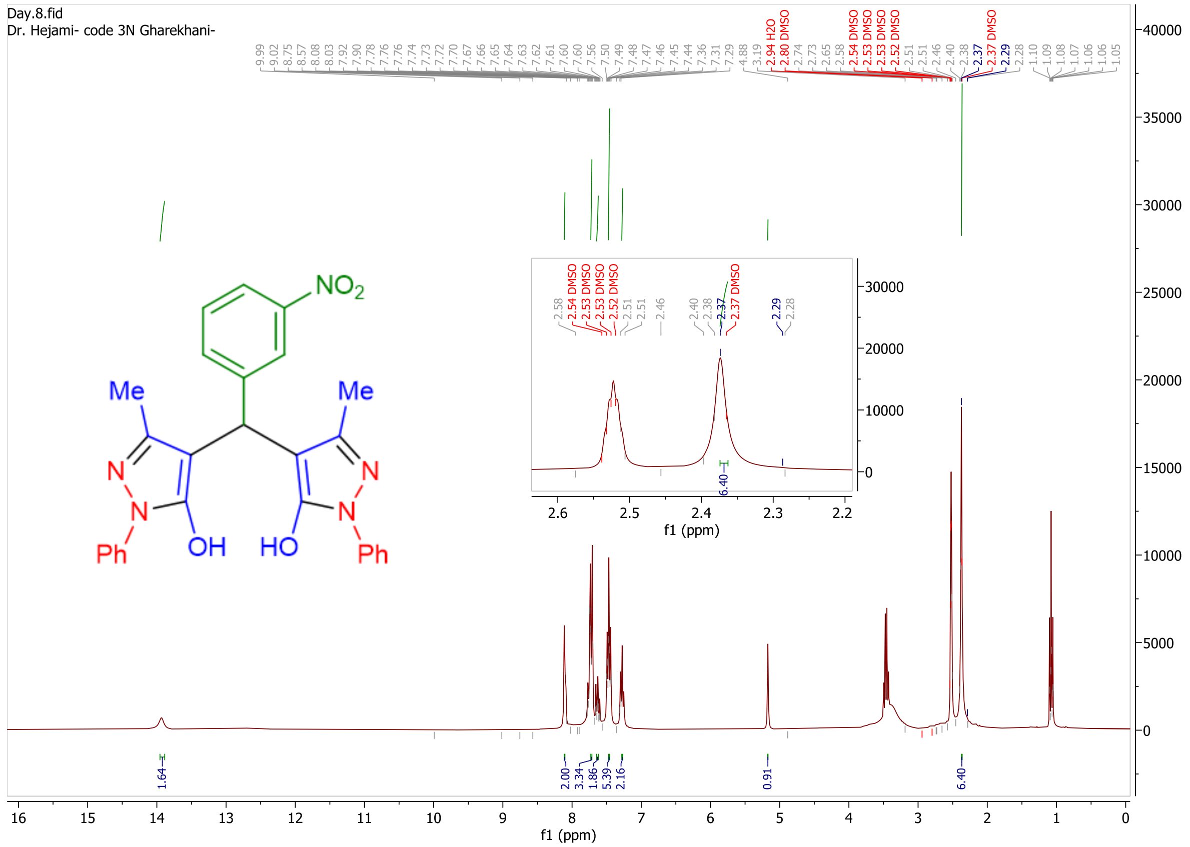

Supplementary figure 12: 4,4'-((3-nitrophenyl)methylene)bis(3-methyl-1-phenyl-1H-pyrazol-5-ol

4,4'-((3-nitrophenyl)methylene)bis(3-methyl-1-phenyl-1H-pyrazol-5-ol: ^{1}H NMR (250 MHz, DMSO): 2.29 (m, 6H), 5.24 (s, 1H), 7.29 (t, J= 7.5 Hz, 2H), 7.44 (m, 5H), 7.56 (m, 2H), 7.61 (t, J= 7.5 Hz, 3H), 8.03 (s, 2H), 13.99 (s, br, 2H) ppm.

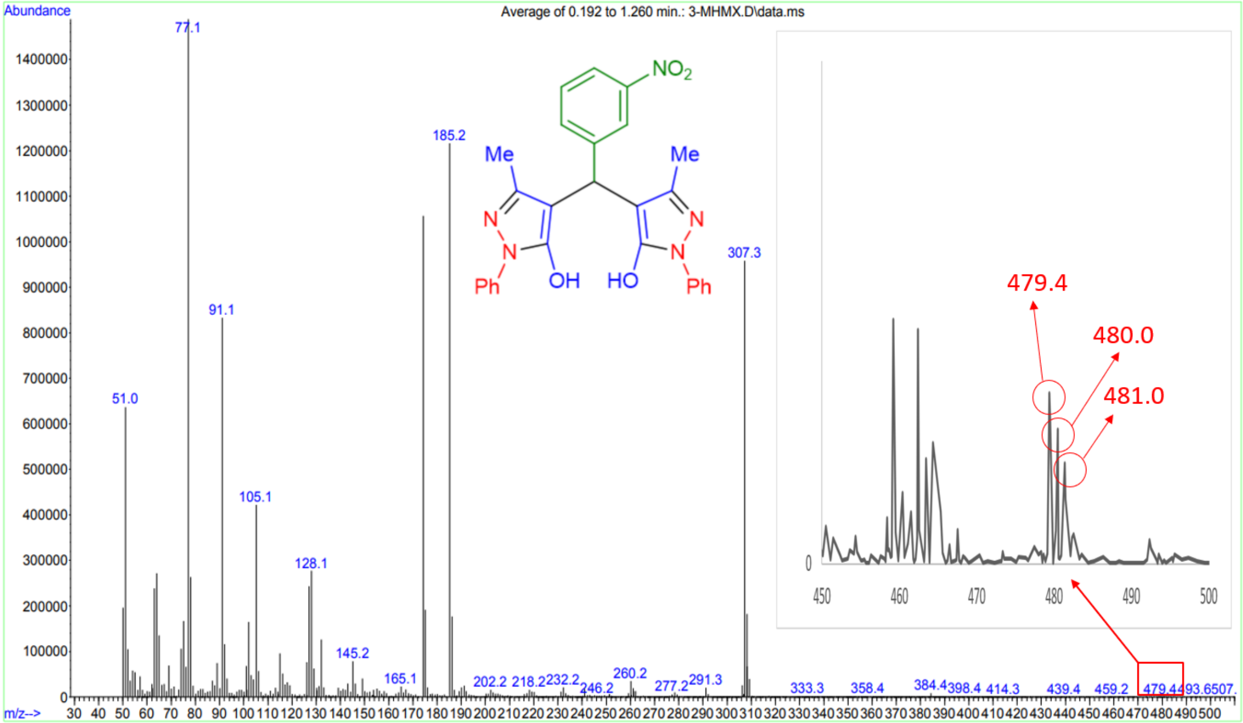

Supplementary figure 13: Mass analysis of 4,4'-((3-nitrophenyl)methylene)bis(3-methyl-1-phenyl-1H-pyrazol-5-ol

Mass analysis: Calculated: M/Z= 481.18, Obtained: M/Z= 481.0

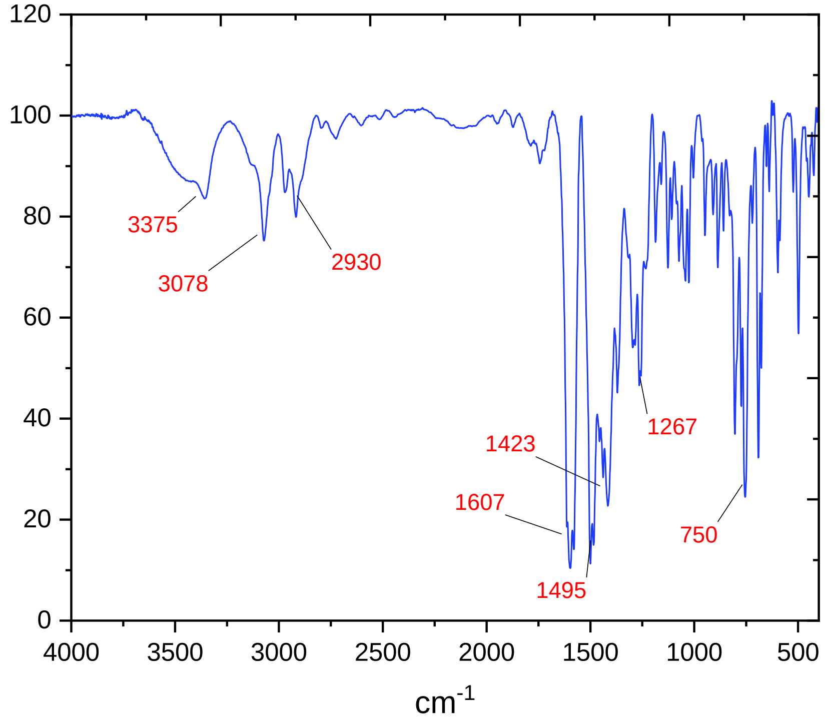

Supplementary figure 14: 4,4'-((3-fluorophenyl)methylene)bis(3-methyl-1-phenyl-1H-pyrazol-5-ol)

4,4'-((3-fluorophenyl)methylene)bis(3-methyl-1-phenyl-1H-pyrazol-5-ol): FT-IR (KBr) cm^{-1}:750, 1267, 1423,1495, 1607, 2930, 3078, 3375.

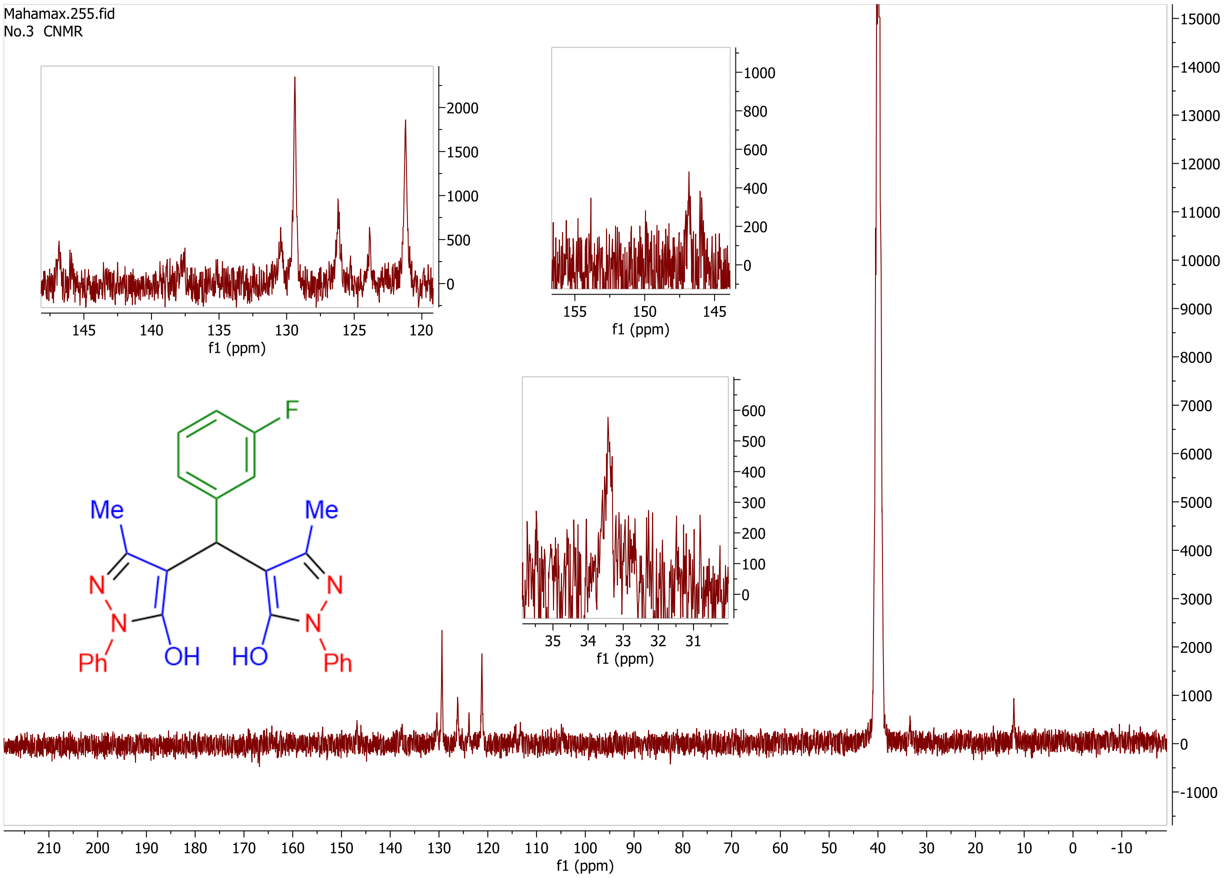

Supplementary figure 15: 4,4'-((3-fluorophenyl)methylene)bis(3-methyl-1-phenyl-1H-pyrazol-5-ol)

^{13}C NMR (62.5 MHz, DMSO): 11.4, 33.7, 120.0, 123.2, 1235.1, 125.3, 129.4,129.8, 135.1, 139.1, 145.2, 147.6, 148.8, 150.4, 155.0 ppm.

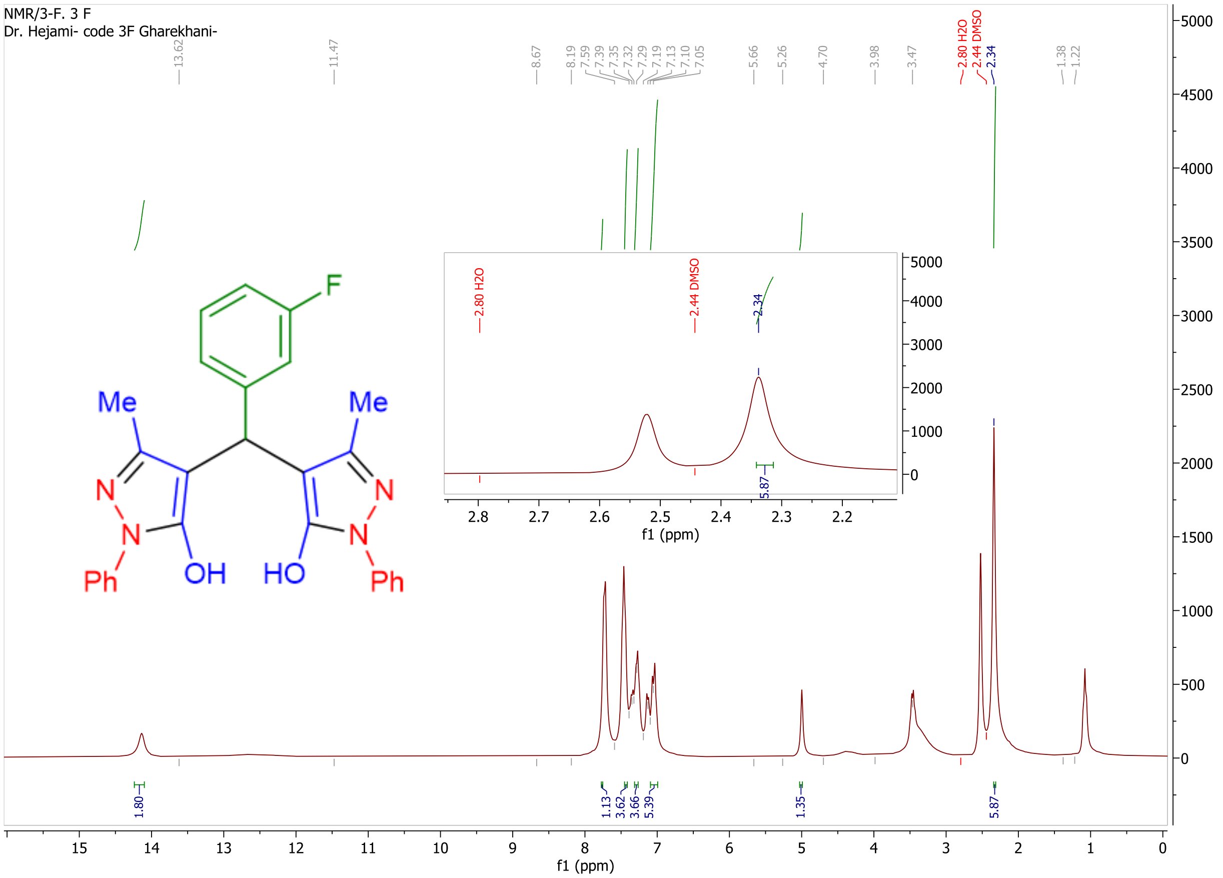

Supplementary figure 16: 4,4'-((3-fluorophenyl)methylene)bis(3-methyl-1-phenyl-1H-pyrazol-5-ol)

4,4'-((3-fluorophenyl)methylene)bis(3-methyl-1-phenyl-1H-pyrazol-5-ol): ^{1}H NMR (250 MHz, DMSO): 2.34 (m, 6H), 5.26 (s, 1H), 7.05-7.59 (m, 14H), 14.20 (s, br, 2H) ppm.

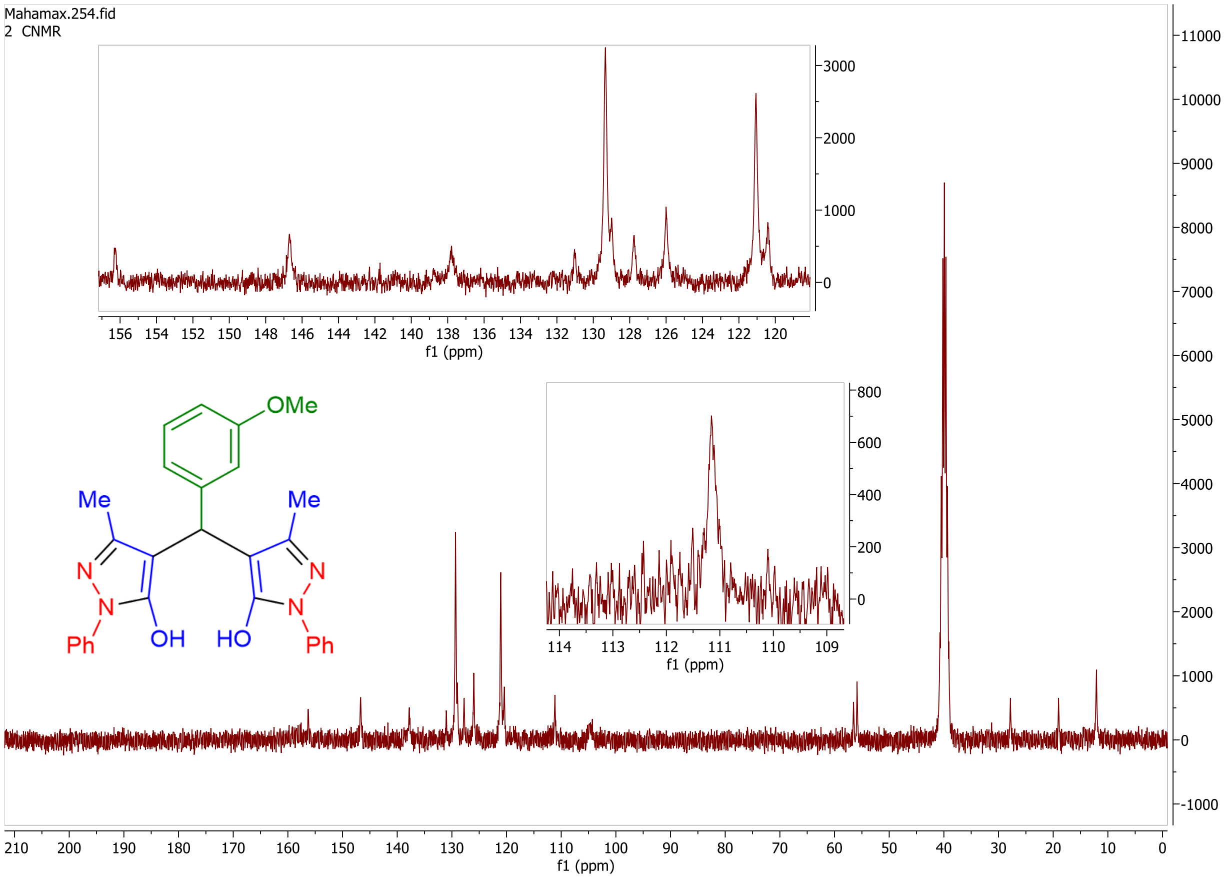

Supplementary figure 17: 4,4'-((3-methoxyphenyl)methylene)bis(3-methyl-1-phenyl-1H-pyrazol-5-ol)

^{13}C NMR (62.5 MHz, DMSO): 13.7, 35.1, 58.2, 111.4, 112.5, 120.2, 120.7, 126.0, 126.9, 128.9, 130.4, 137.9, 142.0, 142.7, 149.1, 156.2 ppm.

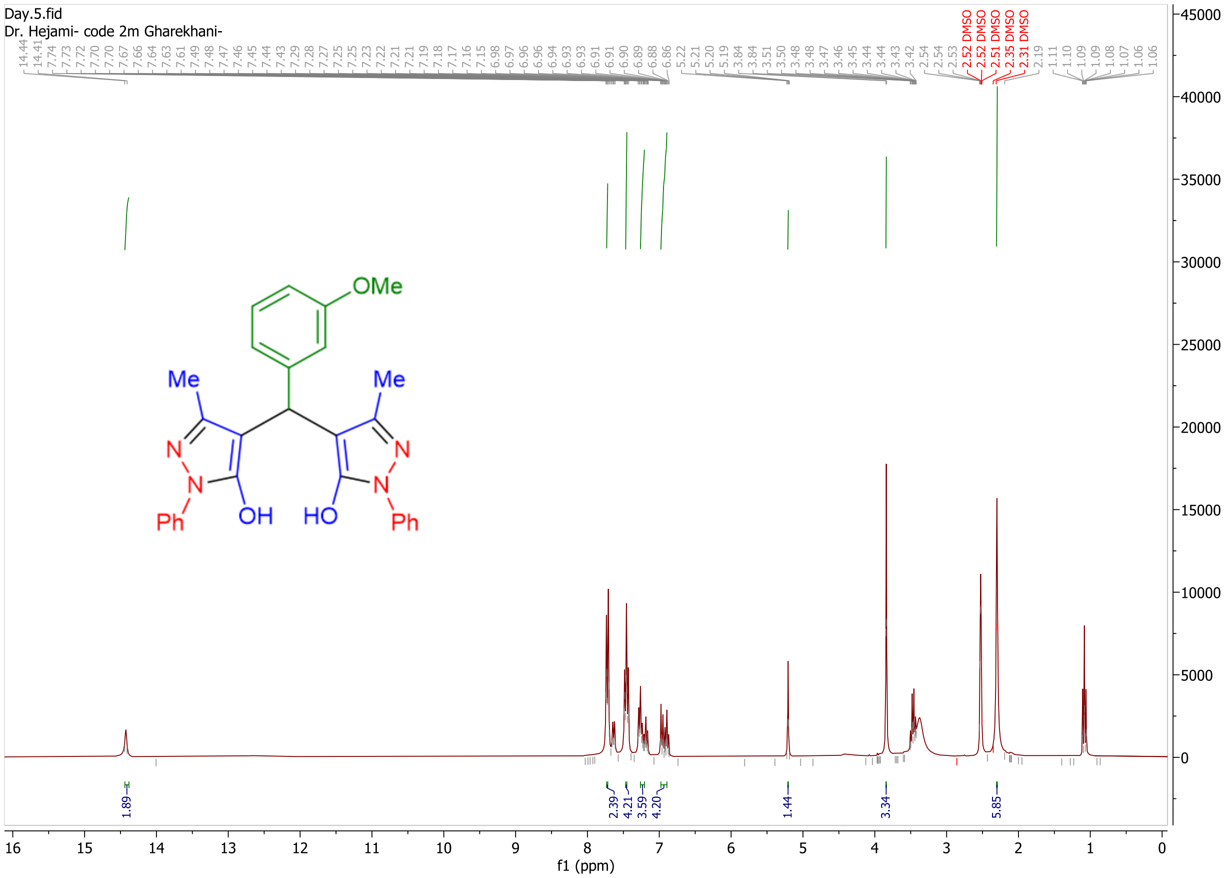

Supplementary figure 18: 4,4'-((3-methoxyphenyl)methylene)bis(3-methyl-1-phenyl-1H-pyrazol-5-ol)

4,4'-((3-methoxyphenyl)methylene)bis(3-methyl-1-phenyl-1H-pyrazol-5-ol): ^{1}H NMR (250 MHz, DMSO): 2.19 (m, 6H), 3.84 (s, 3H), 5.22 (s, 1H), 7.15-7. 27 (m, 8H), 7. 43 (t, J= 7.5, 4H), 7.72 (t, J= 7.5, 2H), 14.44 (s, br, 2H) ppm.

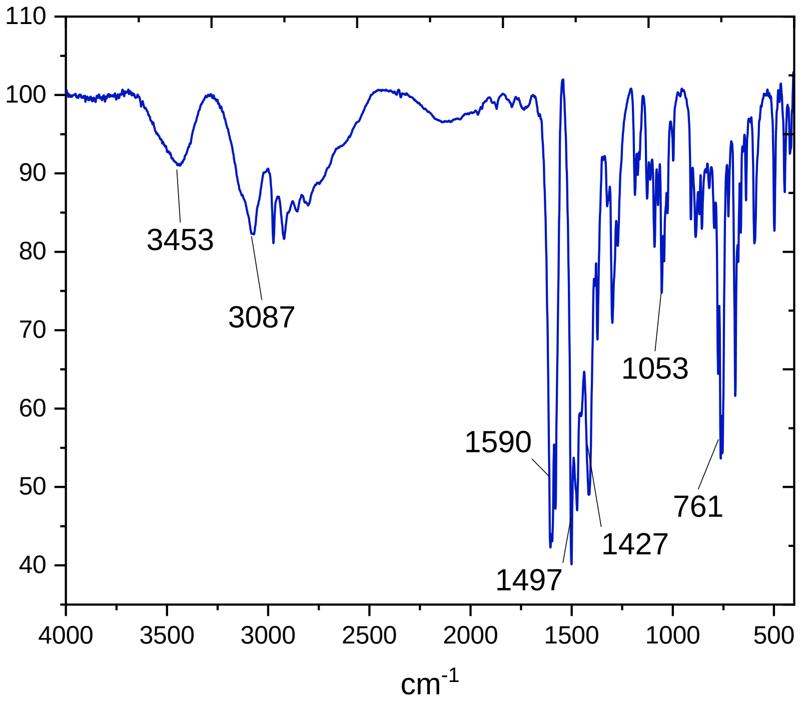

Supplementary figure 19: 4,4'-((3-bromophenyl)methylene)bis(3-methyl-1-phenyl-1H-pyrazol-5-ol)

4,4'-((3-bromophenyl)methylene)bis(3-methyl-1-phenyl-1H-pyrazol-5-ol): FT-IR (KBr) cm^{-1}:761, 1053, 1427, 1497, 1590, 3087, 3453.

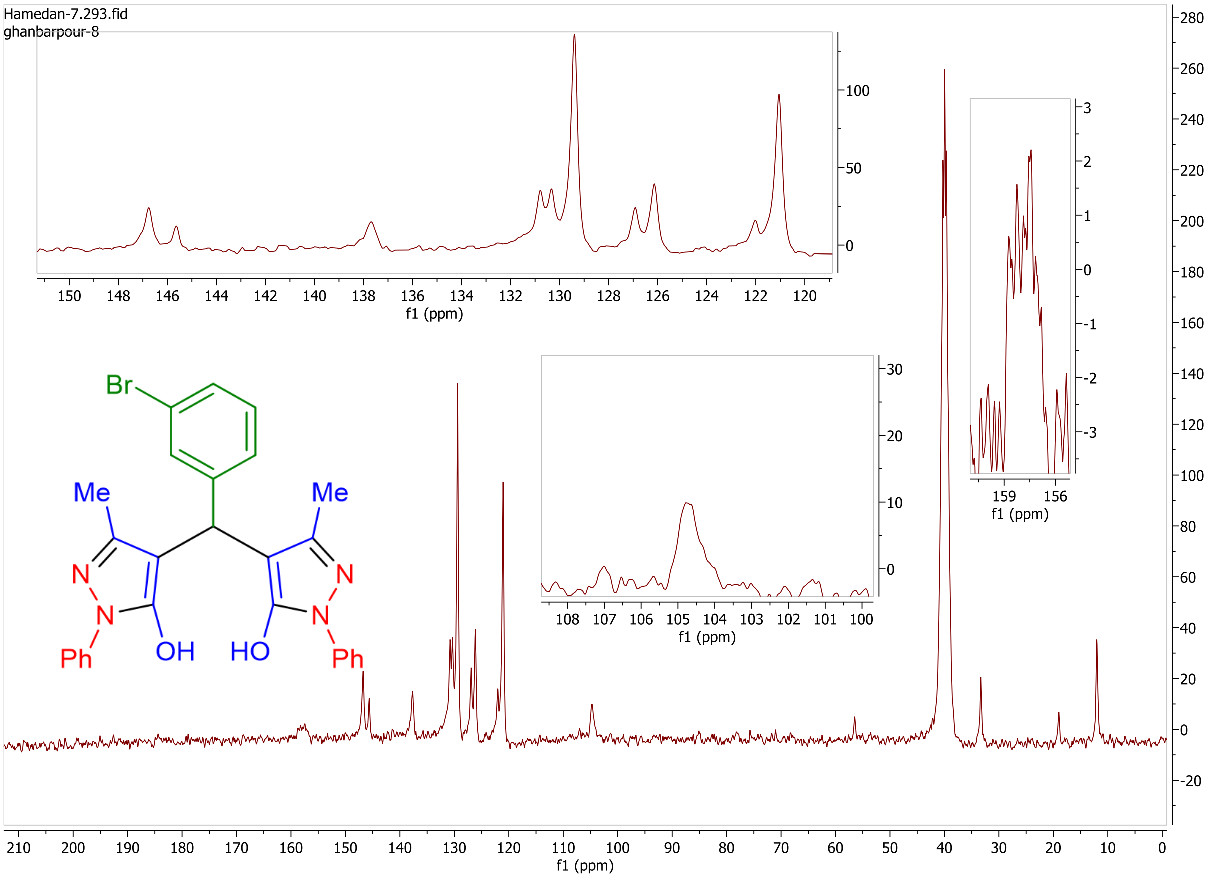

Supplementary figure 20: 4,4'-((3-bromophenyl)methylene)bis(3-methyl-1-phenyl-1H-pyrazol-5-ol)

^{13}C NMR (62.5 MHz, DMSO): 11.7, 35.1, 120.3, 120.4, 125.3, 126.5, 128.1, 130.2, 130.4, 137.9, 146.1, 149.3, 156.2, 156.5, 157.0 ppm.

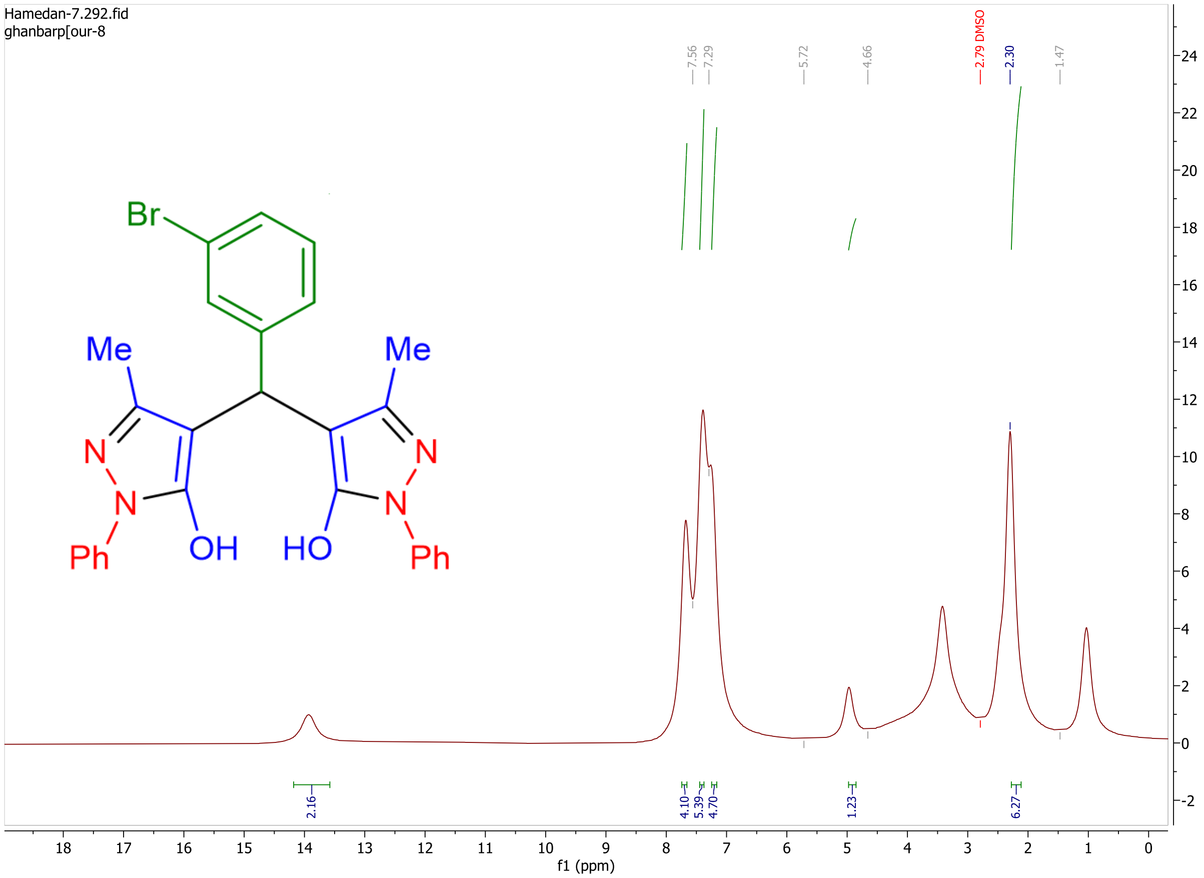

Supplementary figure 21: 4,4'-((3-bromophenyl)methylene)bis(3-methyl-1-phenyl-1H-pyrazol-5-ol)

4,4'-((3-bromophenyl)methylene)bis(3-methyl-1-phenyl-1H-pyrazol-5-ol): ^{1}H NMR (250 MHz, DMSO): 2.30 (m, 6H), 4.66 (s, 1H), 7.29-7. 56 (m, 14H), 14.04 (s, br, 2H) ppm.

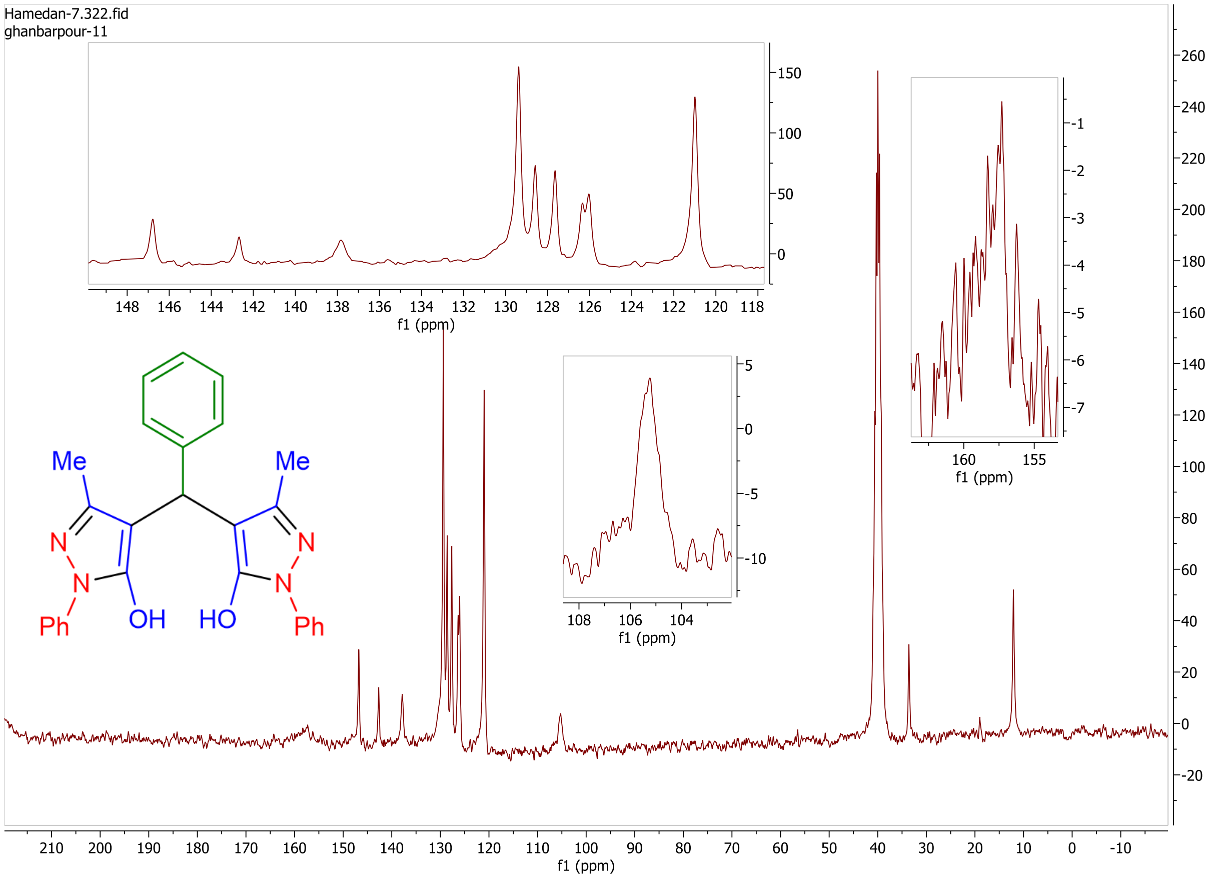

Supplementary figure 22: 4,4'-(phenylmethylene)bis(3-methyl-1-phenyl-1H-pyrazol-5-ol)

^{13}C NMR (62.5 MHz, DMSO): 10.2, 35.9, 105.5, 120.0, 125.9, 127.4, 128.5, 129.7, 134.8, 137.4, 142.6, 147.0, 155.1, 157.0, 158.3 ppm.

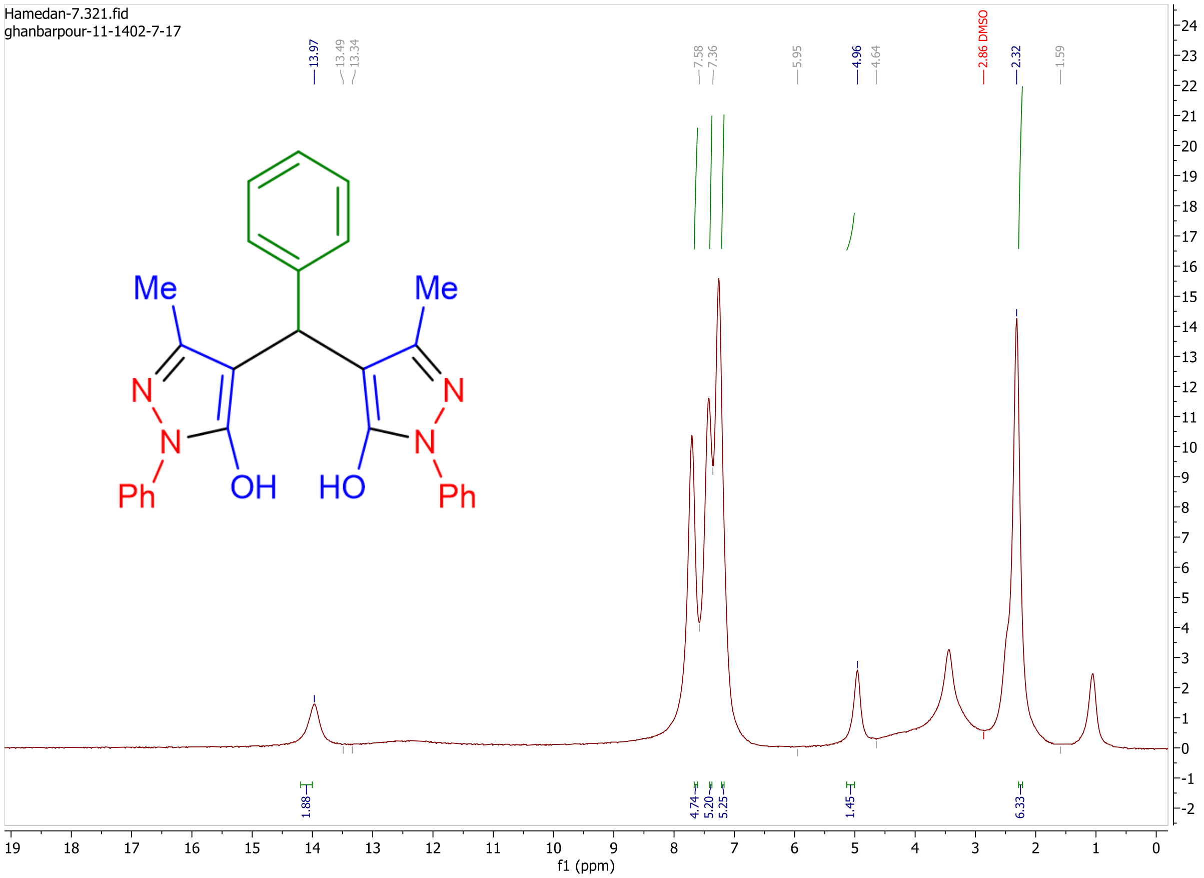

Supplementary figure 23: 4,4'-(phenylmethylene)bis(3-methyl-1-phenyl-1H-pyrazol-5-ol)

4,4'-(phenylmethylene)bis(3-methyl-1-phenyl-1H-pyrazol-5-ol): ^{1}H NMR (250 MHz, DMSO): 2.32 (m, 6H), 4.64 (s, 1H), 7.36-7. 58 (m, 15H), 14.00 (s, br, 2H) ppm.

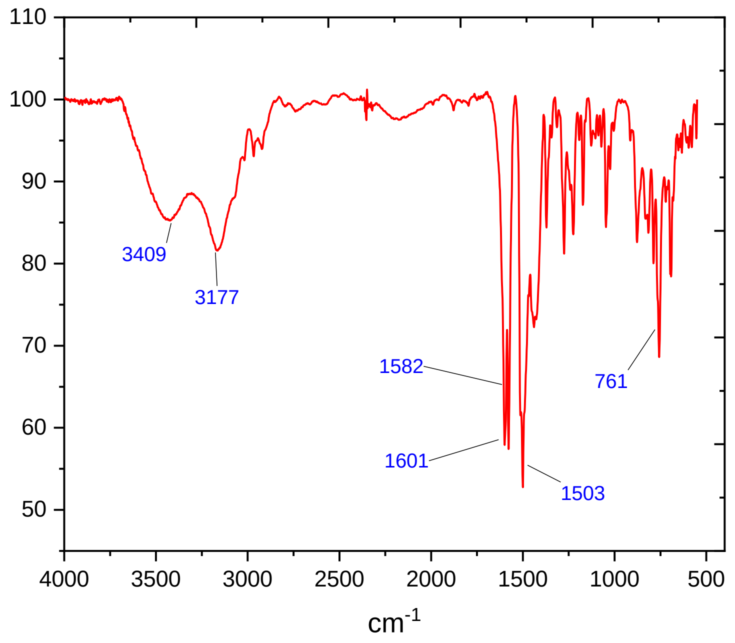

Supplementary figure 24: 4,4'-((4-hydroxyphenyl)methylene)bis(3-methyl-1-phenyl-1H-pyrazol-5-ol)

4,4'-((4-hydroxyphenyl)methylene)bis(3-methyl-1-phenyl-1H-pyrazol-5-ol): FT-IR (KBr) cm^{-1}: 761, 1503, 1582, 1601, 3177, 3409.

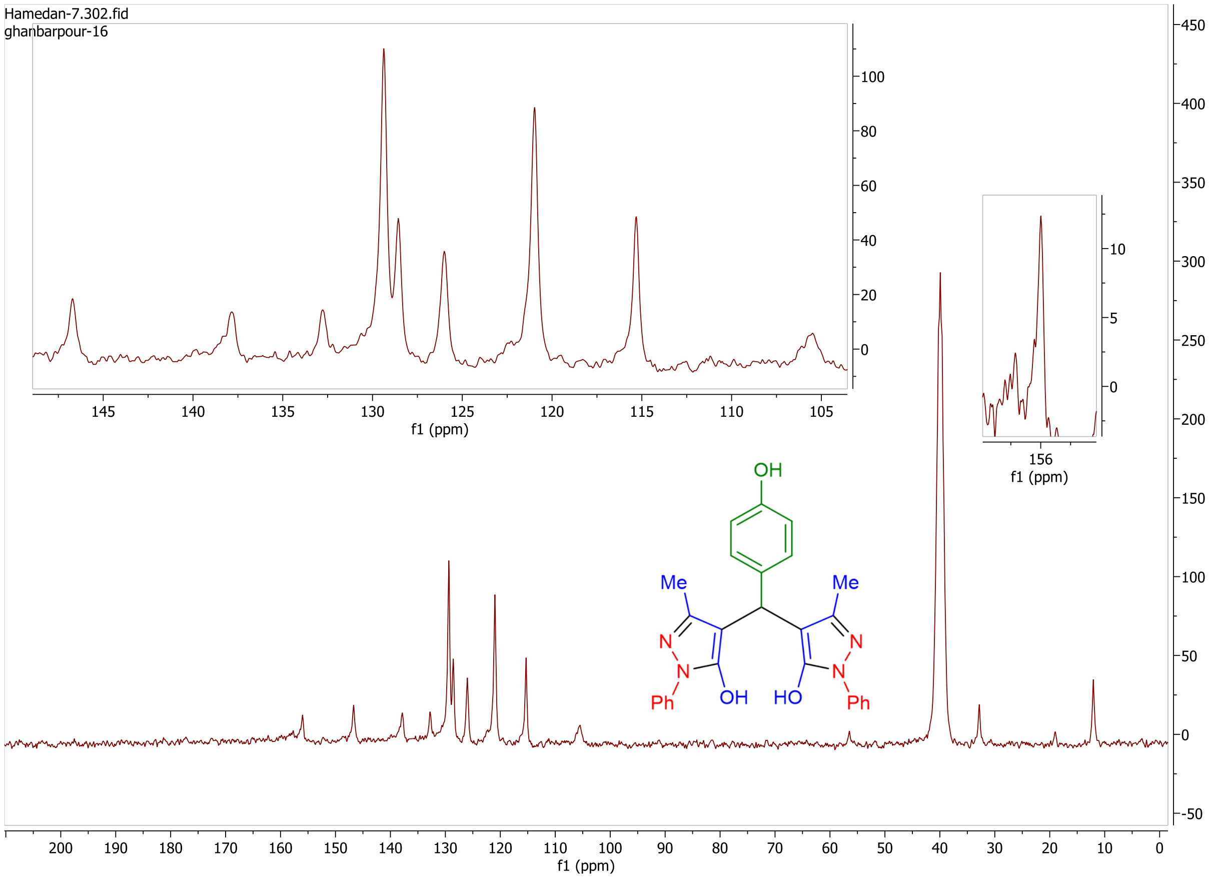

Supplementary figure 25: 4,4'-((4-hydroxyphenyl)methylene)bis(3-methyl-1-phenyl-1H-pyrazol-5-ol)

^{13}C NMR (62.5 MHz, DMSO): 9.0, 34.6, 110.5, 115.4, 119.9, 120.3, 125.2, 127.1, 129.1, 132.5, 137.9, 146.2, 156.4, 157.1 ppm.

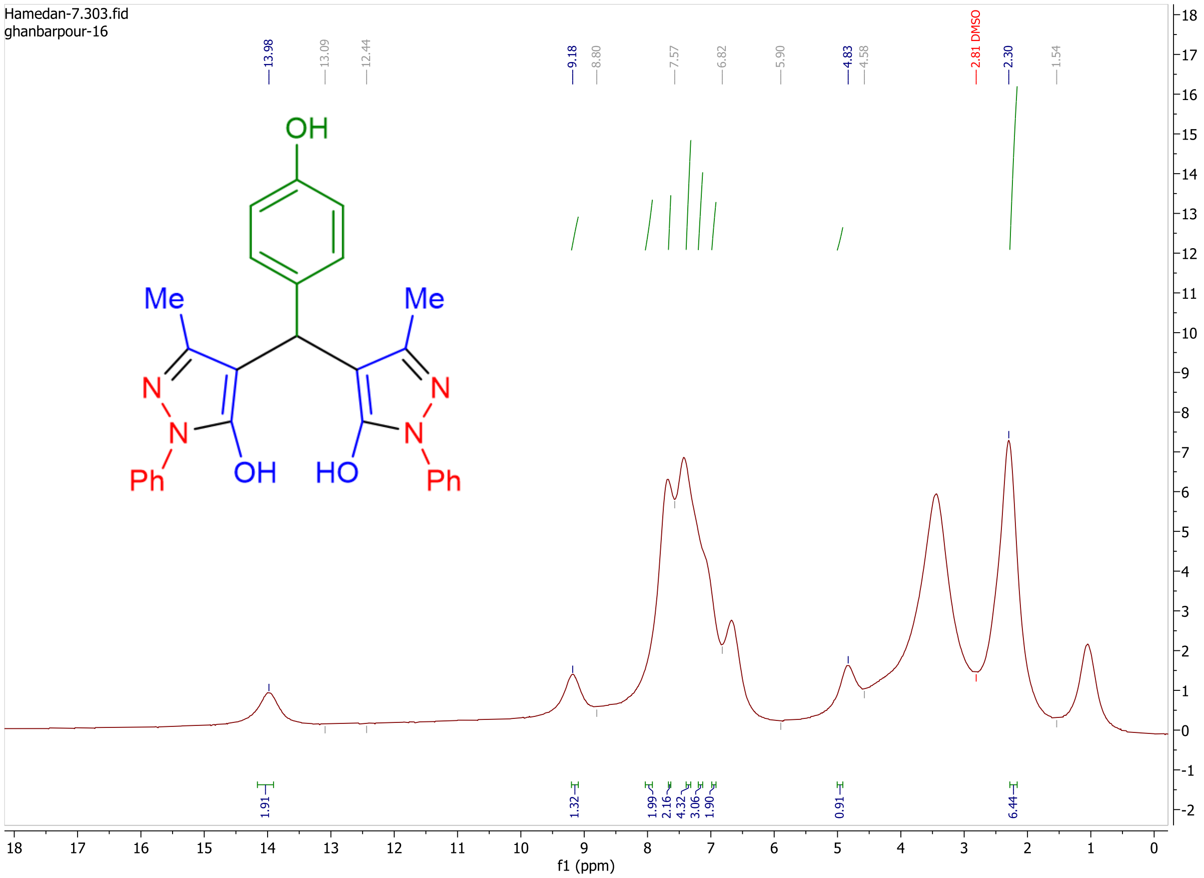

Supplementary figure 26: 4,4'-((4-hydroxyphenyl)methylene)bis(3-methyl-1-phenyl-1H-pyrazol-5-ol)

4,4'-((3-bromophenyl)methylene)bis(3-methyl-1-phenyl-1H-pyrazol-5-ol): ^{1}H NMR (250 MHz, DMSO): 2.30 (m, 6H), 4.58 (s, 1H), 6.82-7. 57 (m, 13H), 8.80 (s, 1H) 14.00 (s, br, 2H) ppm.

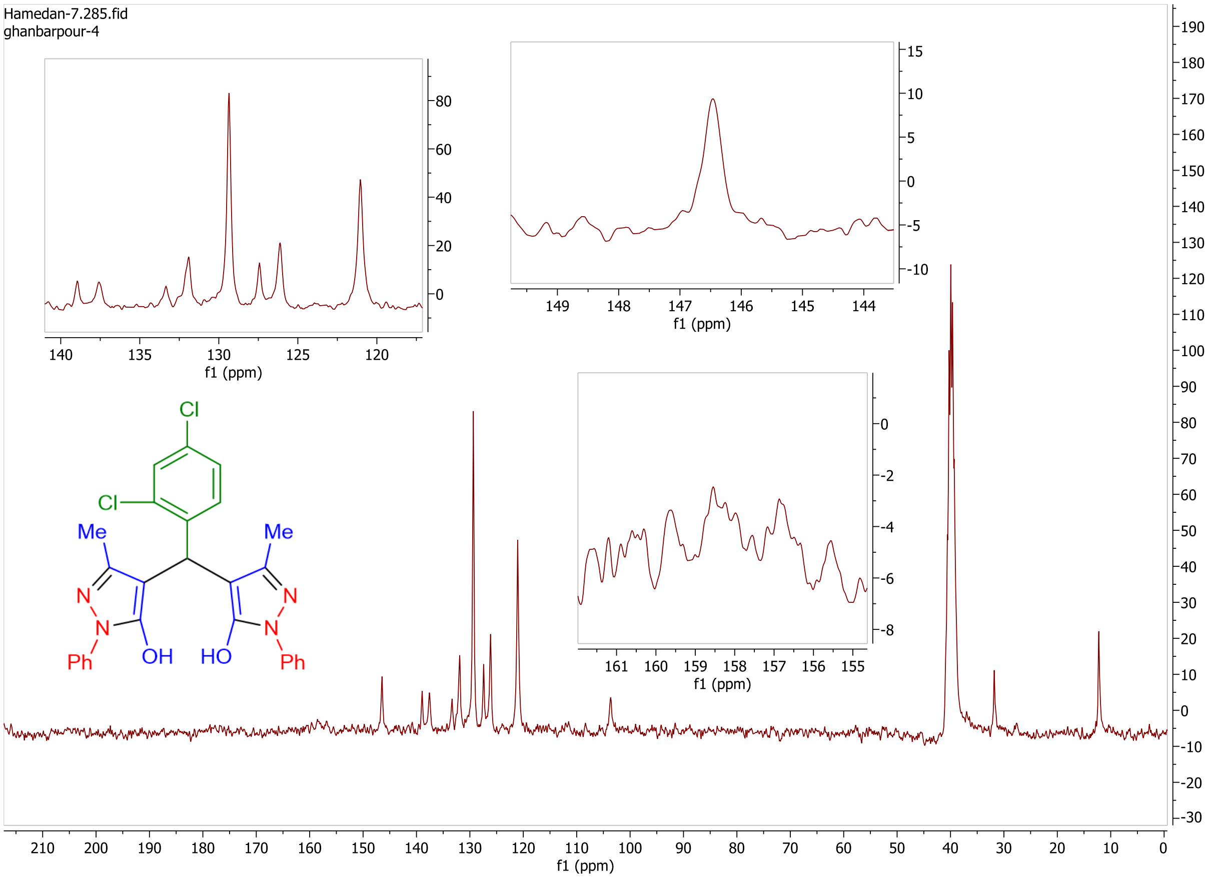

Supplementary figure 27: 4,4'-((2,4-dichlorophenyl)methylene)bis(3-methyl-1-phenyl-1H-pyrazol-5-ol)

^{13}C NMR (62.5 MHz, DMSO): 10.6, 33.9, 120.6, 125.1, 127.0, 128.2, 129.5, 131.9, 132.2, 137.1, 139.3, 147.1, 155.5, 157.2, 158.5, 159.8 ppm.

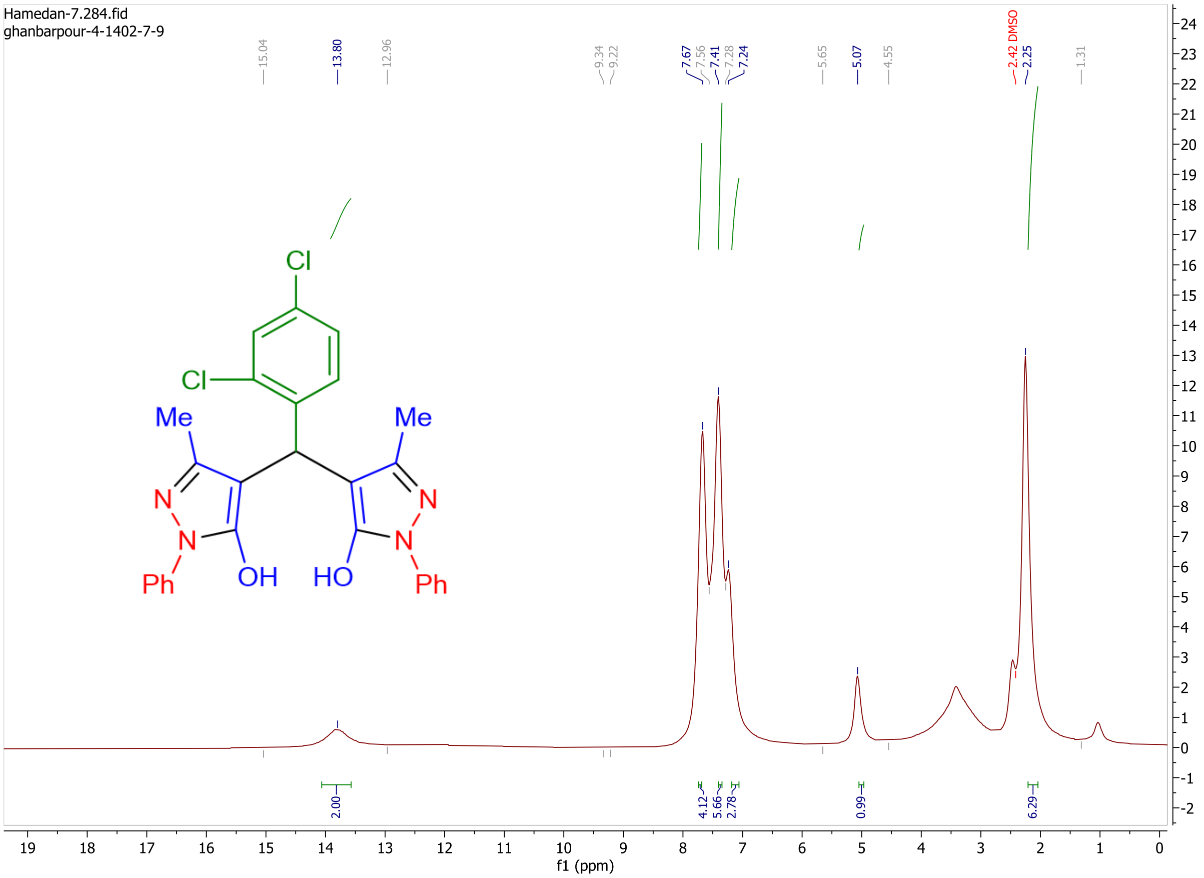

Supplementary figure 28: 4,4'-((2,4-dichlorophenyl)methylene)bis(3-methyl-1-phenyl-1H-pyrazol-5-ol)

4,4'-((2,4-dichlorophenyl)methylene)bis(3-methyl-1-phenyl-1H-pyrazol-5-ol): ^{1}H NMR (250 MHz, DMSO): 2.25 (m, 6H), 5.07 (s, 1H), 7.24-7.67 (m, 13H), 13.80 (s, br, 2H) ppm.

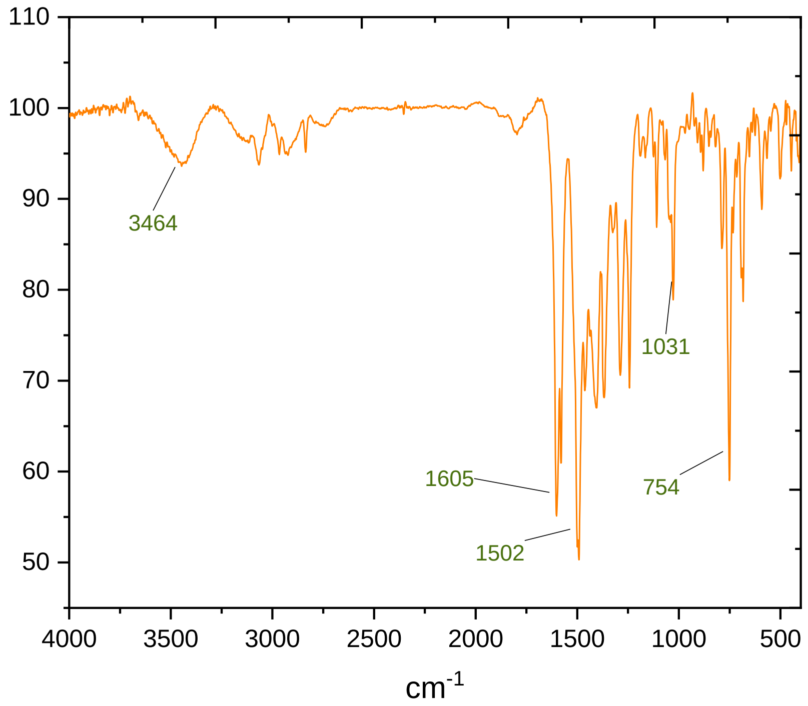

Supplementary figure 29: 4,4'-((2-methoxyphenyl)methylene)bis(3-methyl-1-phenyl-1H-pyrazol-5-ol)

4,4'-((2-methoxyphenyl)methylene)bis(3-methyl-1-phenyl-1H-pyrazol-5-ol): FT-IR (KBr) cm^{-1}: 754, 1031, 1502, 1605, 3464.

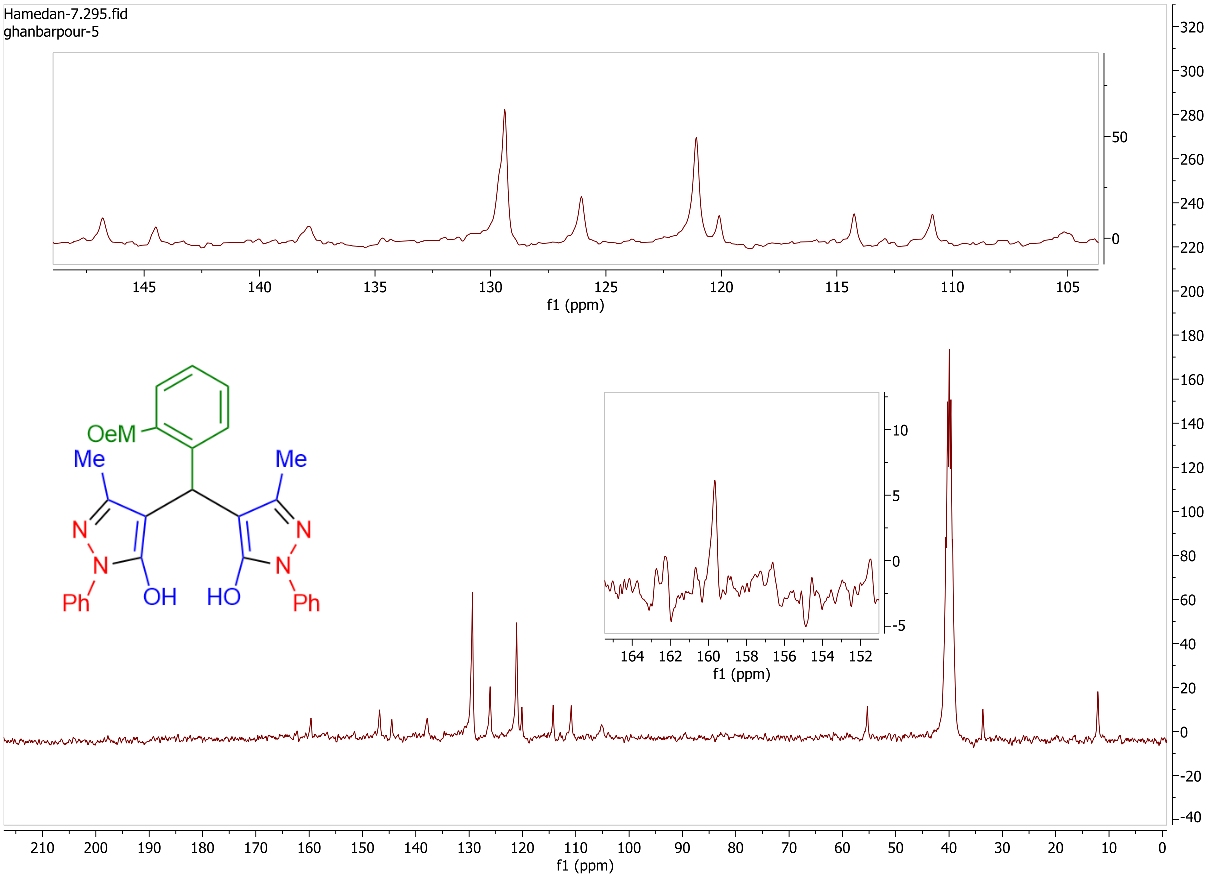

Supplementary figure 30: 4,4'-((2-methoxyphenyl)methylene)bis(3-methyl-1-phenyl-1H-pyrazol-5-ol)

^{13}C NMR (62.5 MHz, DMSO): 11.8, 35.3, 110.8, 114.2, 118.7, 1201.4, 126.4, 129.2, 134.4, 137.5, 144,6, 146.7, 151.1, 156.3, 16.3 ppm.

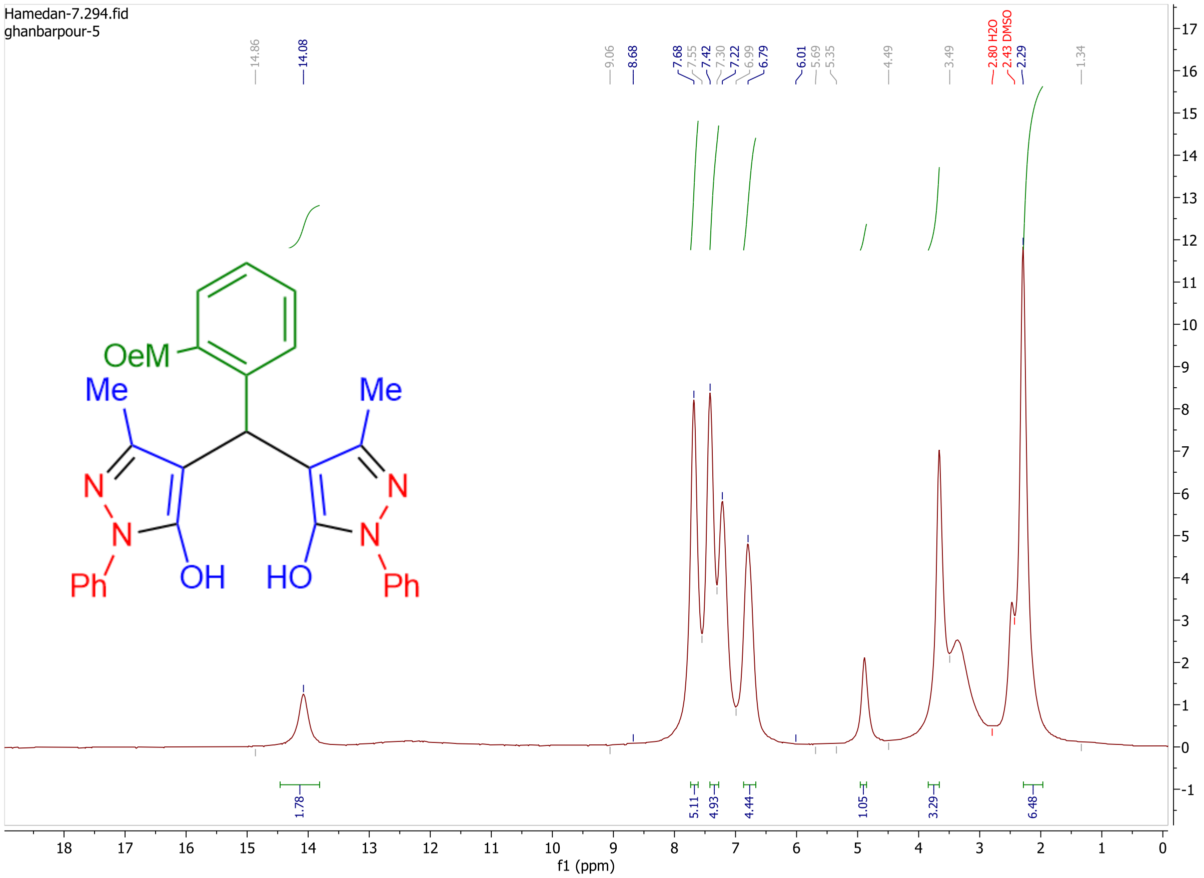

Supplementary figure 31: 4,4'-((2-methoxyphenyl)methylene)bis(3-methyl-1-phenyl-1H-pyrazol-5-ol)

4,4'-((2-methoxyphenyl)methylene)bis(3-methyl-1-phenyl-1H-pyrazol-5-ol): ^{1}H NMR (250 MHz, DMSO): 2.29 (m, 6H), 4.49 (s, 1H), 6.79-7.68 (m, 14H), 14.08 (s, br, 2H) ppm.

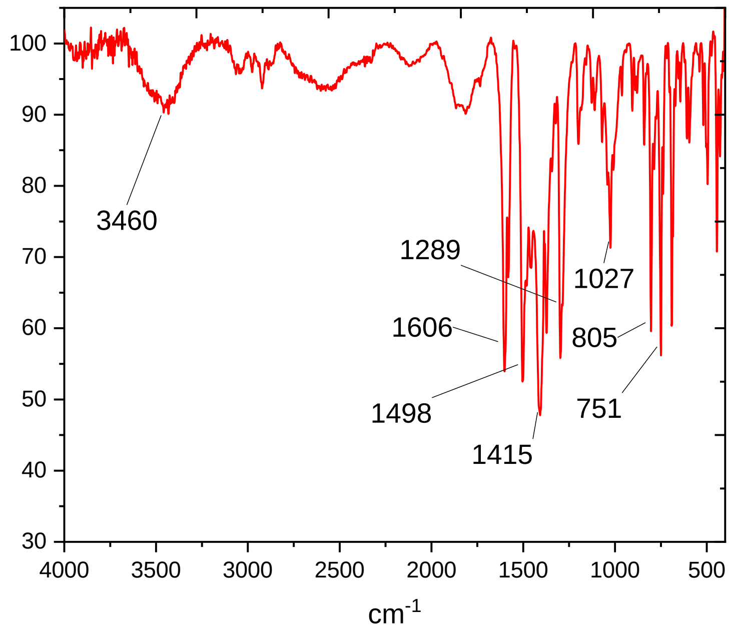

Supplementary figure 32: 4,4'-(p-tolylmethylene)bis(3-methyl-1-phenyl-1H-pyrazol-5-ol)

4,4'-(p-tolylmethylene)bis(3-methyl-1-phenyl-1H-pyrazol-5-ol): FT-IR (KBr) cm^{-1}:751, 805, 1027, 1289, 1498, 1606, 3460.

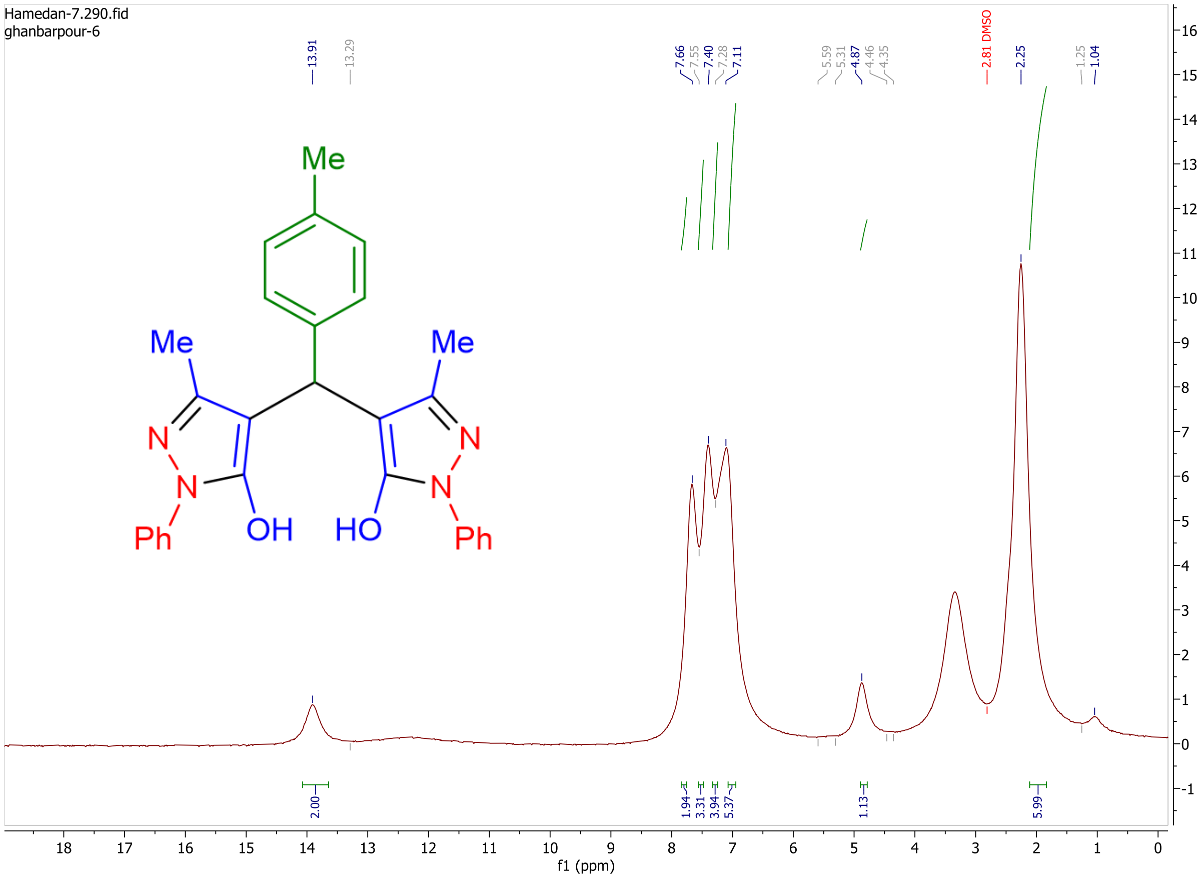

Supplementary figure 33: 4,4'-(p-tolylmethylene)bis(3-methyl-1-phenyl-1H-pyrazol-5-ol)

4,4'-(p-tolylmethylene)bis(3-methyl-1-phenyl-1H-pyrazol-5-ol): ^{1}H NMR (250 MHz, DMSO): 2.25 (m, 6H), 4.87 (s, 1H), 7.11-7.66 (m, 14H), 13.91 (s, br, 2H) ppm.

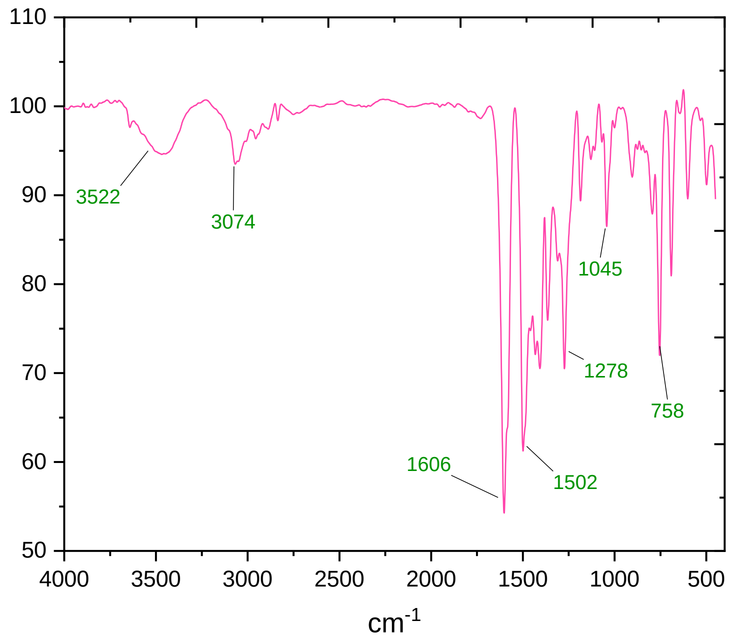

Supplementary figure 34: 4,4'-((4-methoxyphenyl)methylene)bis(3-methyl-1-phenyl-1H-pyrazol-5-ol)

4,4'-((4-methoxyphenyl)methylene)bis(3-methyl-1-phenyl-1H-pyrazol-5-ol): FT-IR (KBr) cm^{-1}:758, 1045, 1278, 1502, 1606, 3074, 3522.

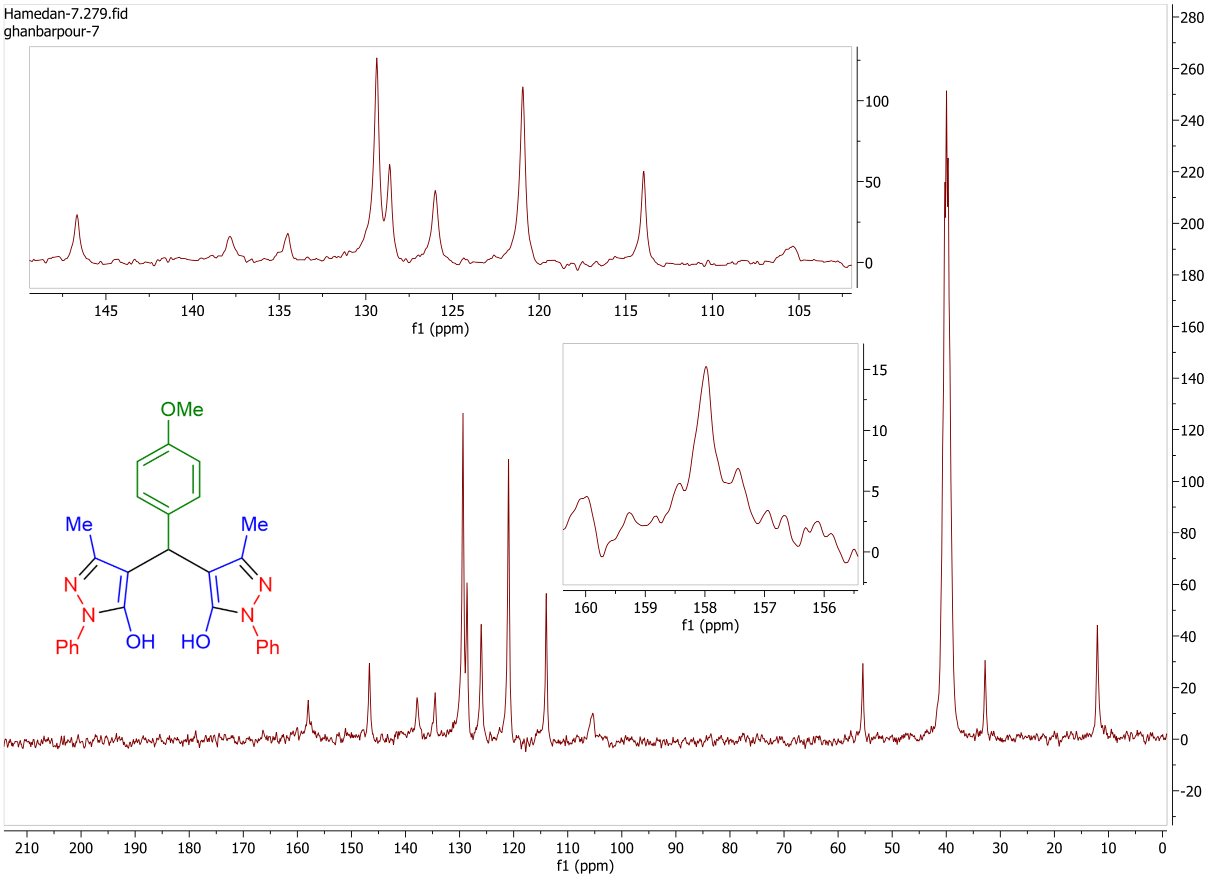

Supplementary figure 35: 4,4'-((4-methoxyphenyl)methylene)bis(3-methyl-1-phenyl-1H-pyrazol-5-ol)

^{13}C NMR (62.5 MHz, DMSO): 13.0, 34.6, 57.0, 112.8, 122.2, 122.7, 125.5, 127.3, 129.9, 134.5, 137.3, 141.9, 156.2, 158.2, 160.1 ppm.

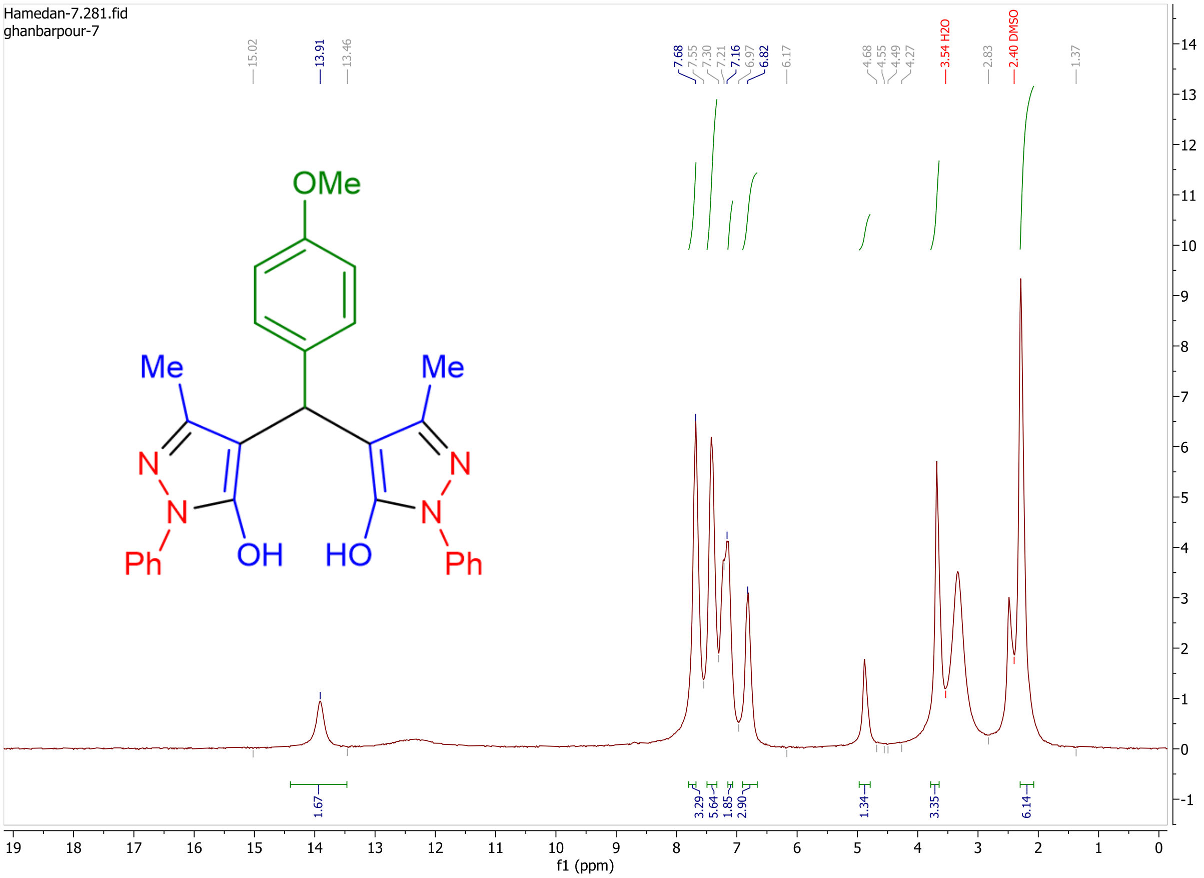

Supplementary figure 36: 4,4'-((4-methoxyphenyl)methylene)bis(3-methyl-1-phenyl-1H-pyrazol-5-ol)

4,4'-((4-methoxyphenyl)methylene)bis(3-methyl-1-phenyl-1H-pyrazol-5-ol): ^{1}H NMR (250 MHz, DMSO): 2.29 (m, 6H), 3.69 (s, 3H), 4.68 (s, 1H), 6.82-7.68 (m, 14H), 13.91 (s, br, 2H) ppm.

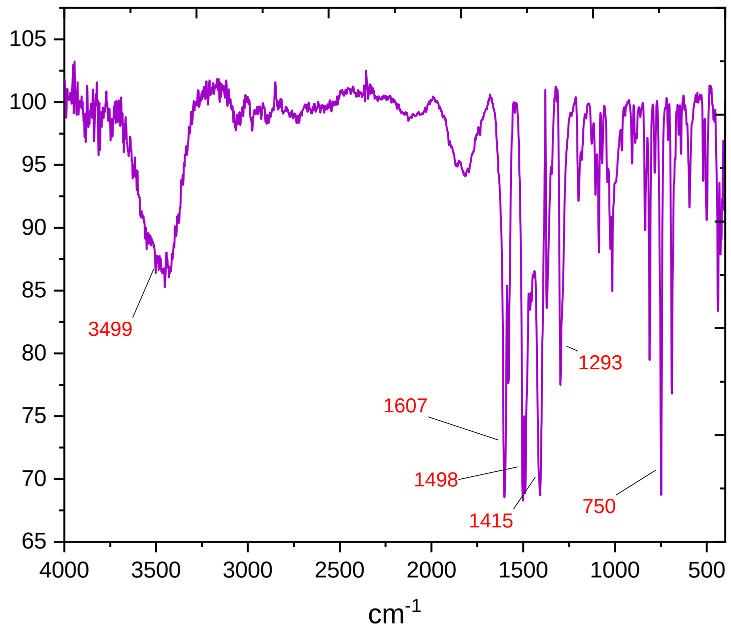

Supplementary figure 37: 4,4'-((4-chlorophenyl)methylene)bis(3-methyl-1-phenyl-1H-pyrazol-5-ol)

4,4'-((4-chlorophenyl)methylene)bis(3-methyl-1-phenyl-1H-pyrazol-5-ol): FT-IR (KBr) cm^{-1}: 750, 1293, 1415, 1498, 1607, 3499.

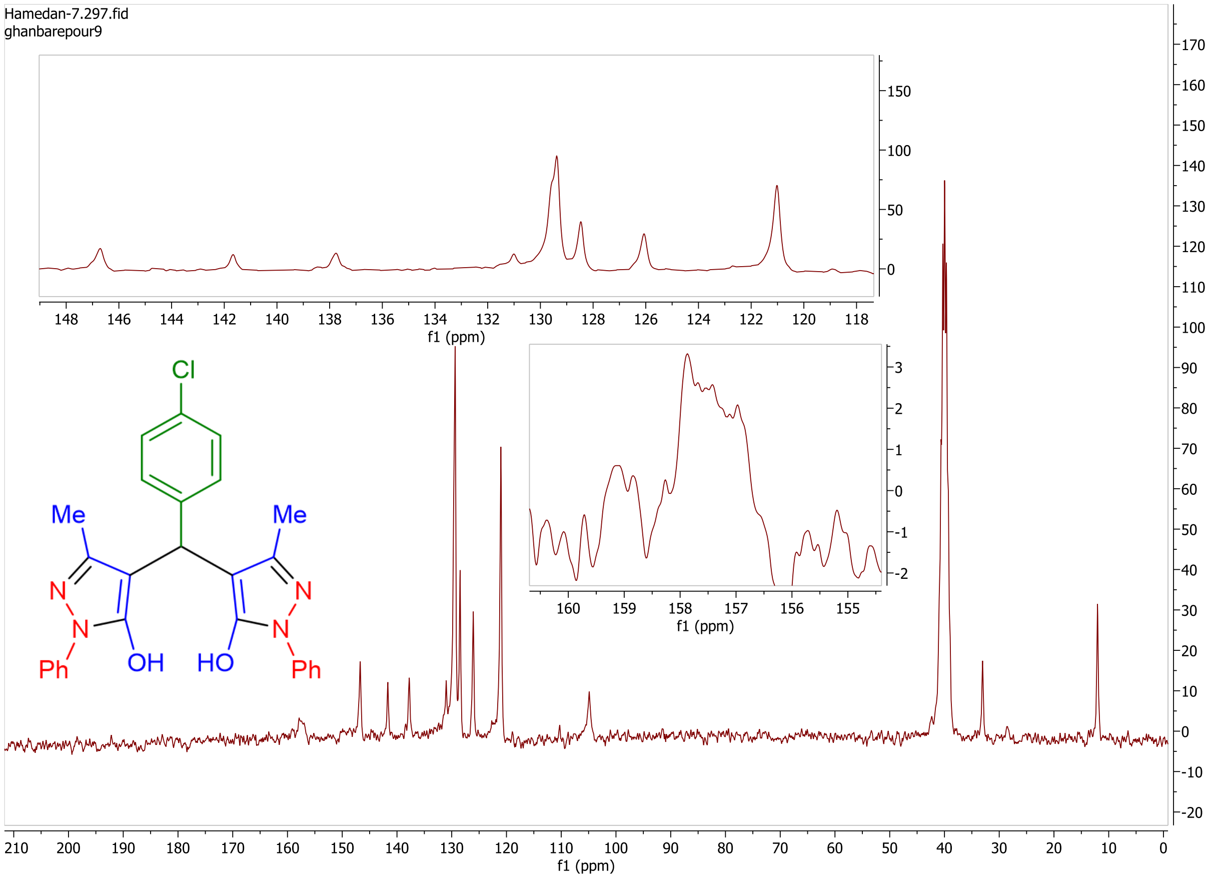

Supplementary figure 38: 4,4'-((4-chlorophenyl)methylene)bis(3-methyl-1-phenyl-1H-pyrazol-5-ol)

^{13}C NMR (62.5 MHz, DMSO): 11.8, 34.1, 118.5, 121.0, 126.1, 128.3, 129.1, 137.5, 138.5, 147.0, 1455.9, 157.3, 158.1, 159.0 ppm.

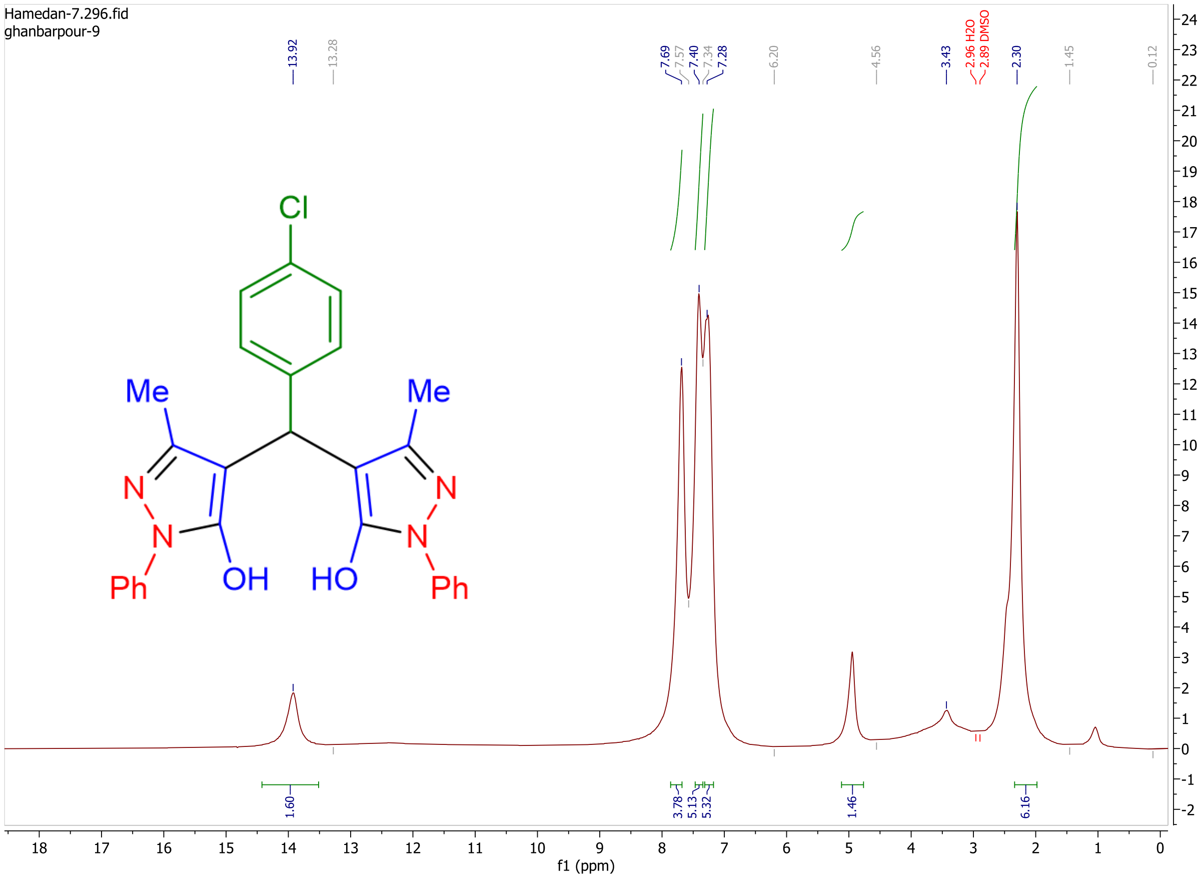

Supplementary figure 39: 4,4'-((4-chlorophenyl)methylene)bis(3-methyl-1-phenyl-1H-pyrazol-5-ol)

4,4'-((4-chlorophenyl)methylene)bis(3-methyl-1-phenyl-1H-pyrazol-5-ol): ^{1}H NMR (250 MHz, DMSO): 2.30 (m, 6H), 4.56 (s, 1H), 7.28-7.69 (m, 14H), 13.92 (s, br, 2H) ppm.

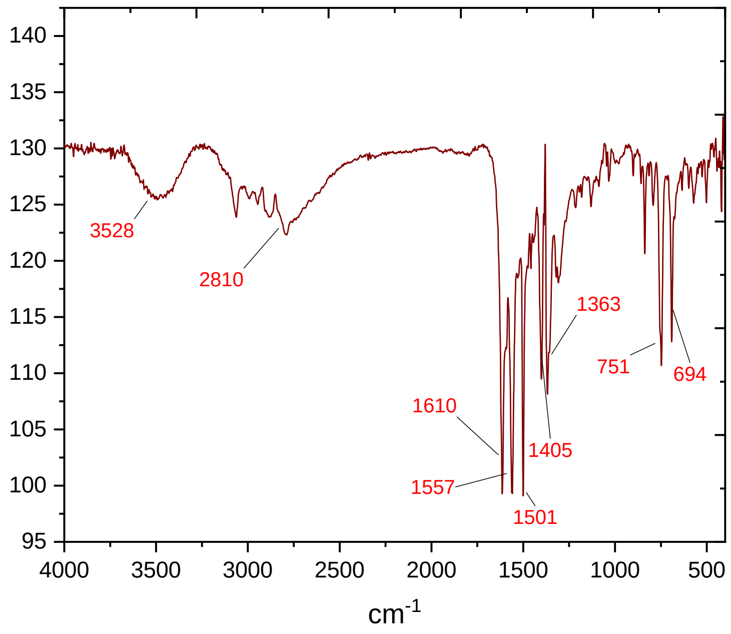

Supplementary figure 40: 4,4'-((3-chlorophenyl)methylene)bis(3-methyl-1-phenyl-1H-pyrazol-5-ol)

4,4'-((3-chlorophenyl)methylene)bis(3-methyl-1-phenyl-1H-pyrazol-5-ol): FT-IR (KBr) cm^{-1}:694, 751, 1363, 1405, 1501, 1557, 1610, 2810, 3528.

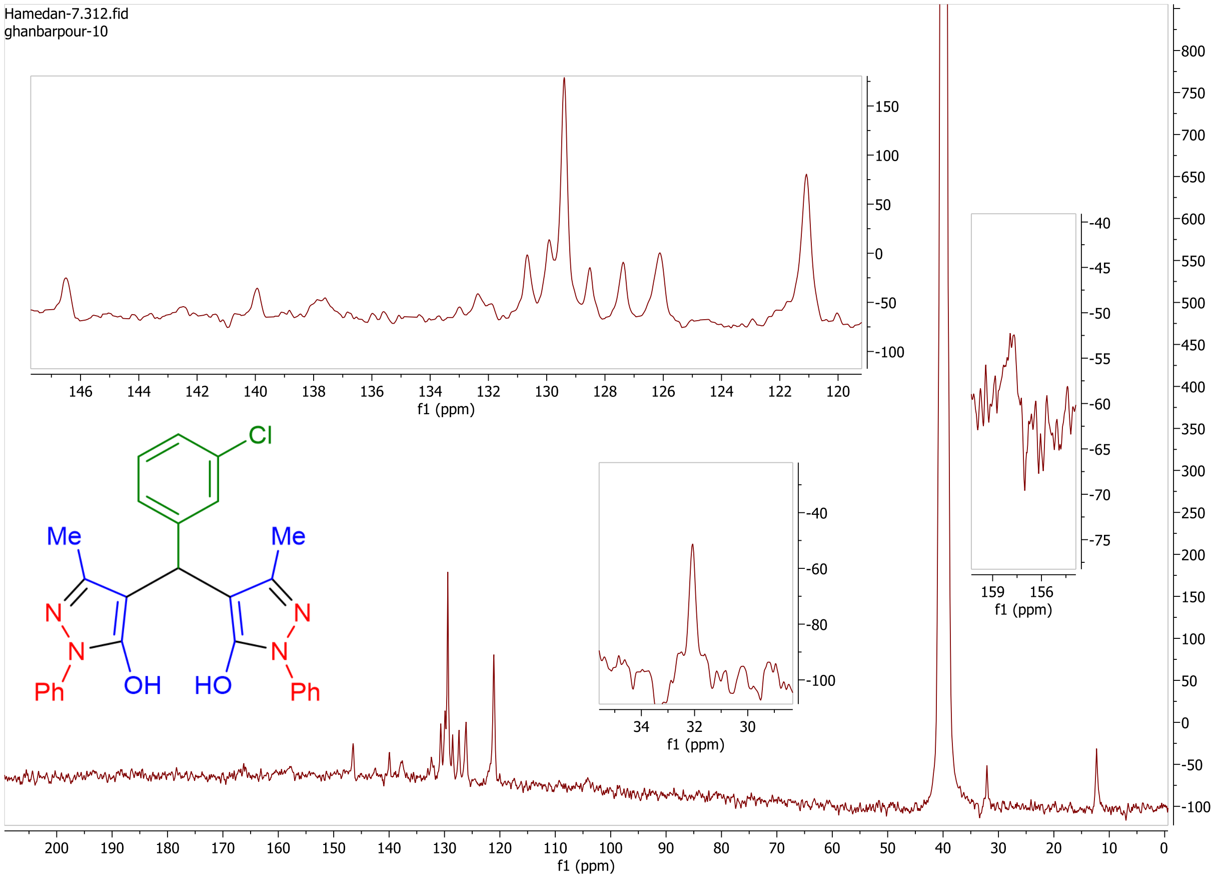

Supplementary figure 41: 4,4'-((3-chlorophenyl)methylene)bis(3-methyl-1-phenyl-1H-pyrazol-5-ol)

^{13}C NMR (62.5 MHz, DMSO): 10.8, 32.5, 120.3, 121.1, 126.8, 127.2, 128.8, 129.2, 130.3, 131.5, 132.2, 137.6, 139.5, 146.4, 157.4 ppm.

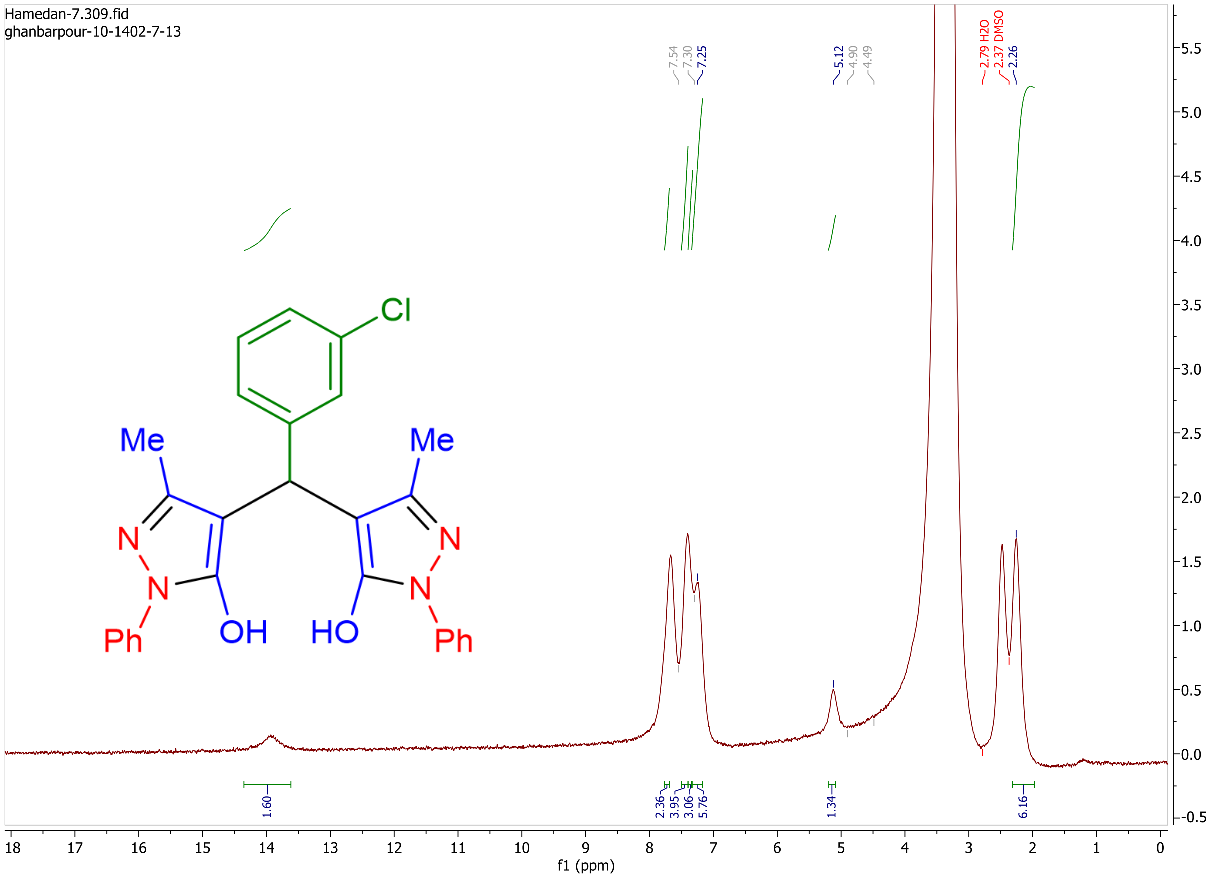

Supplementary figure 42: 4,4'-((3-chlorophenyl)methylene)bis(3-methyl-1-phenyl-1H-pyrazol-5-ol)

4,4'-((3-chlorophenyl)methylene)bis(3-methyl-1-phenyl-1H-pyrazol-5-ol): ^{1}H NMR (250 MHz, DMSO): 2.26 (m, 6H), 5.12 (s, 1H), 7.25-7.54 (m, 14H), 13.93 (s, br, 2H) ppm.

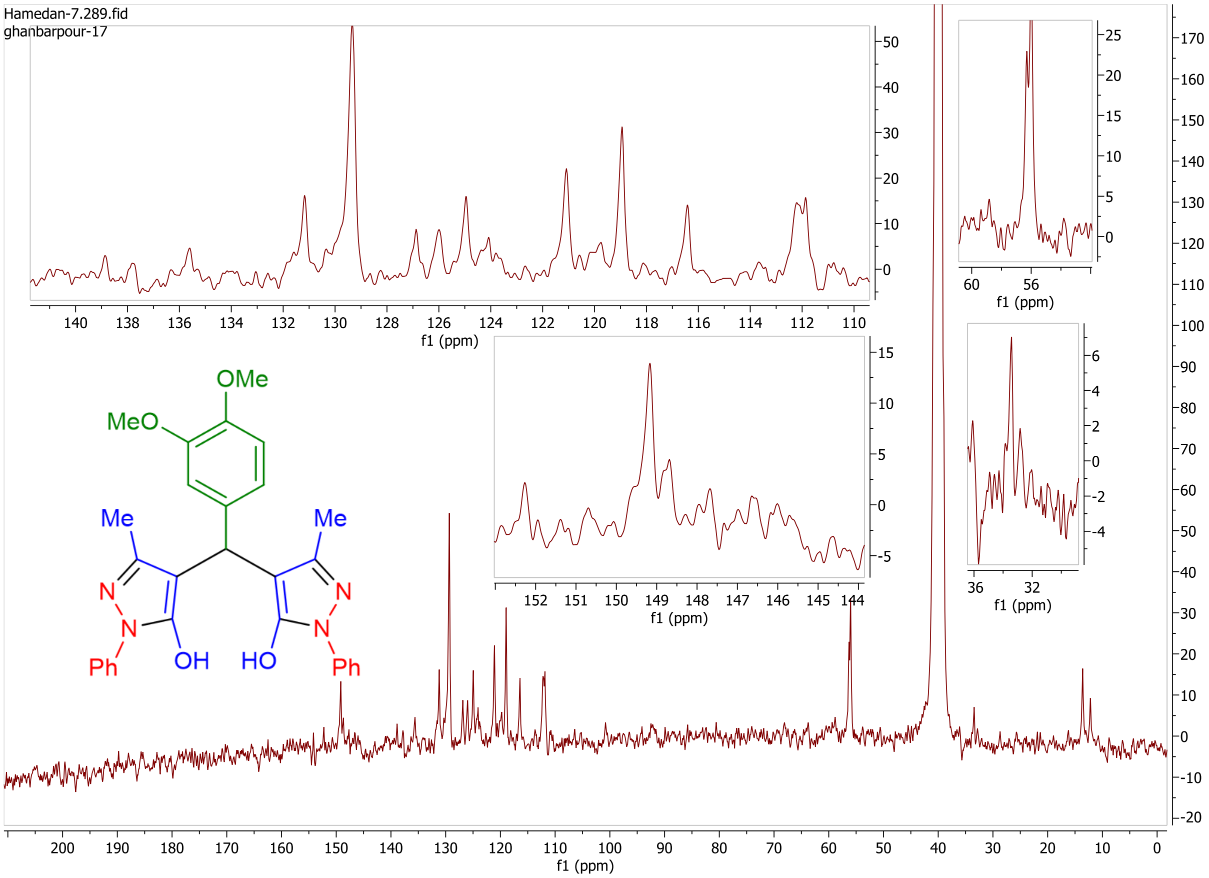

Supplementary figure 43: 4,4'-((3,4-dimethoxyphenyl)methylene)bis(3-methyl-1-phenyl-1H-pyrazol-5-ol)

^{13}C NMR (62.5 MHz, DMSO): 11.1, 35.6, 57.8, 112.0, 116.3, 121.3, 124.6, 125.6, 127.4, 130.6, 131.4, 135.8, 148.6, 139.8, 147.3, 149.6, 152.4 ppm.

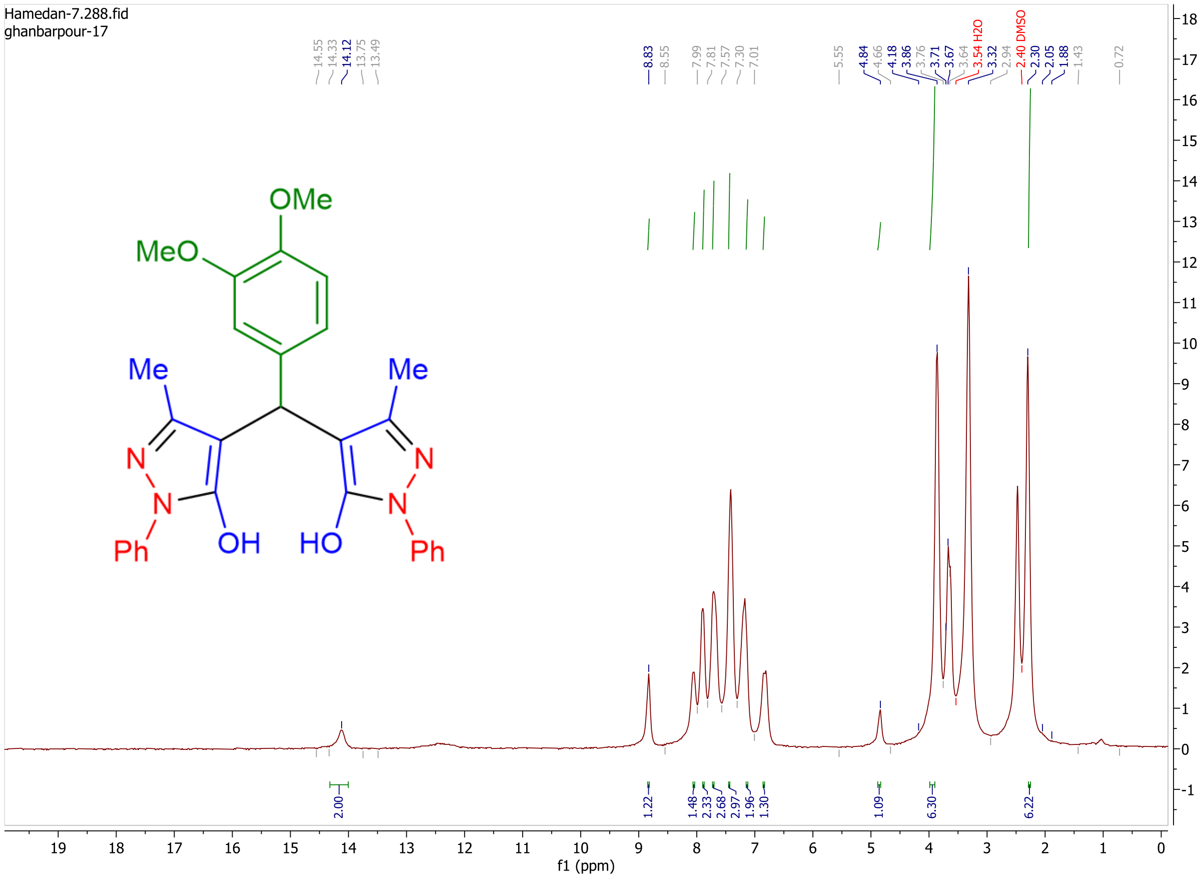

Supplementary figure 44: 4,4'-((3,4-dimethoxyphenyl)methylene)bis(3-methyl-1-phenyl-1H-pyrazol-5-ol)

4,4'-((3,4-dimethoxyphenyl)methylene)bis(3-methyl-1-phenyl-1H-pyrazol-5-ol): ^{1}H NMR (250 MHz, DMSO): 2.26 (m, 6H), 4.84 (s, 3H), 4.84 (s, 1H), 7.01-7.99 (m, 12H), 8.83 (s, 1H), 14.12 (s, br, 2H) ppm.

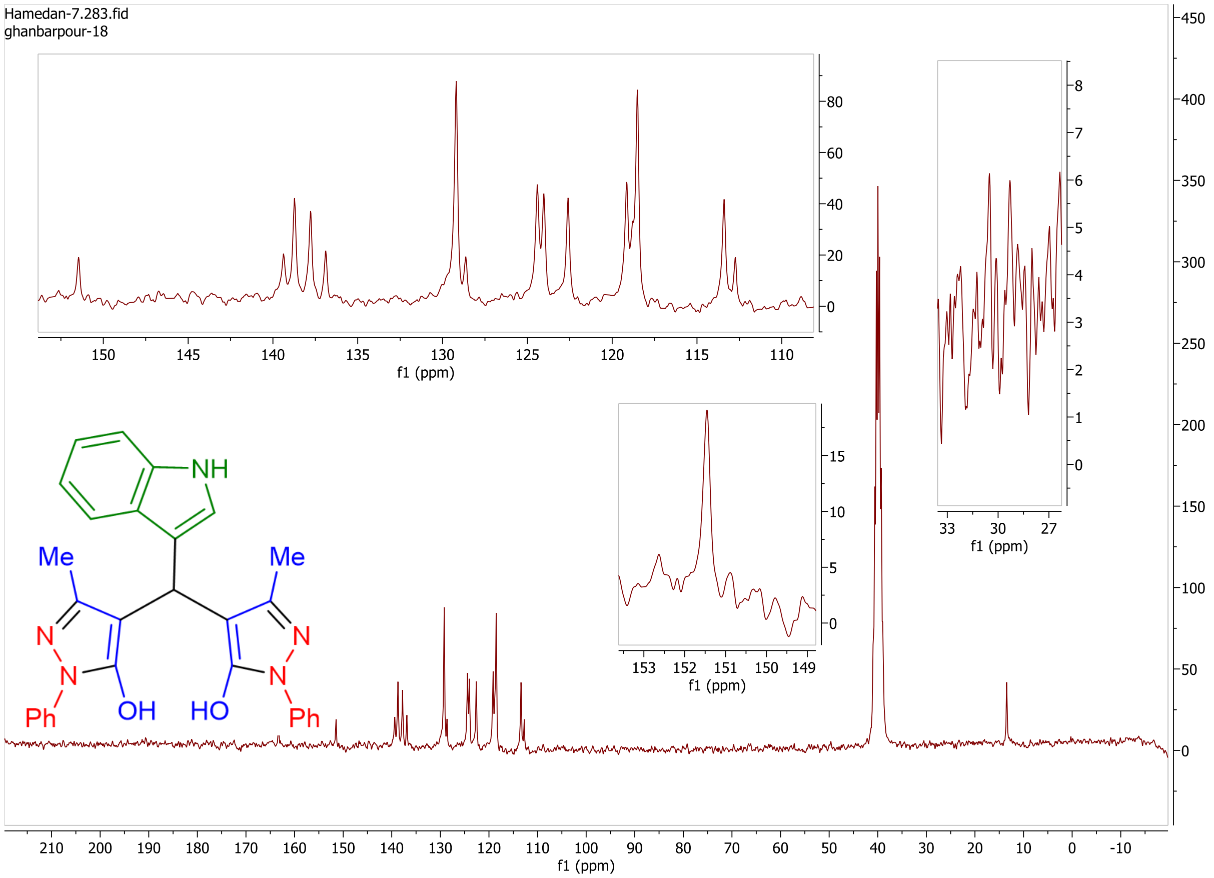

Supplementary figure 45: 4,4'-((1H-indol-3-yl)methylene)bis(3-methyl-1-phenyl-1H-pyrazol-5-ol)

^{13}C NMR (62.5 MHz, DMSO): 13.7, 31.1, 112.9, 114.4, 119.0, 122.4, 123.9, 124.3, 127.4, 128.8, 129.1, 130.0, 133.8, 137.2, 138.6, 140.6, 152.0, 153.4 ppm.

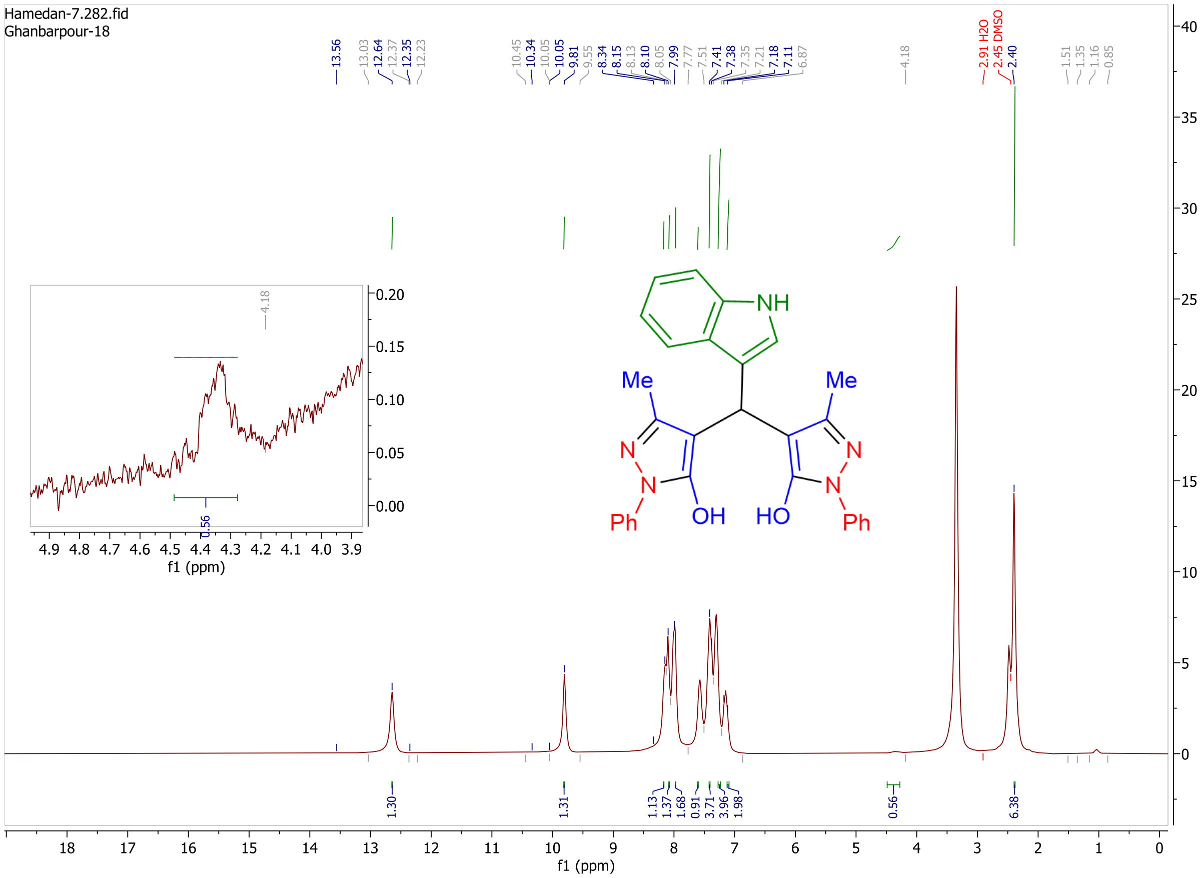

Supplementary figure 46: 4,4'-((1H-indol-3-yl)methylene)bis(3-methyl-1-phenyl-1H-pyrazol-5-ol)

4,4'-((1H-indol-3-yl)methylene)bis(3-methyl-1-phenyl-1H-pyrazol-5-ol): ^{1}H NMR (250 MHz, DMSO): 2.40 (m, 6H), 4.18 (s, 1H), 6.87-8.34 (m, 15H), 10.05 (s, 1H), 13.56 (s, 2H) ppm.

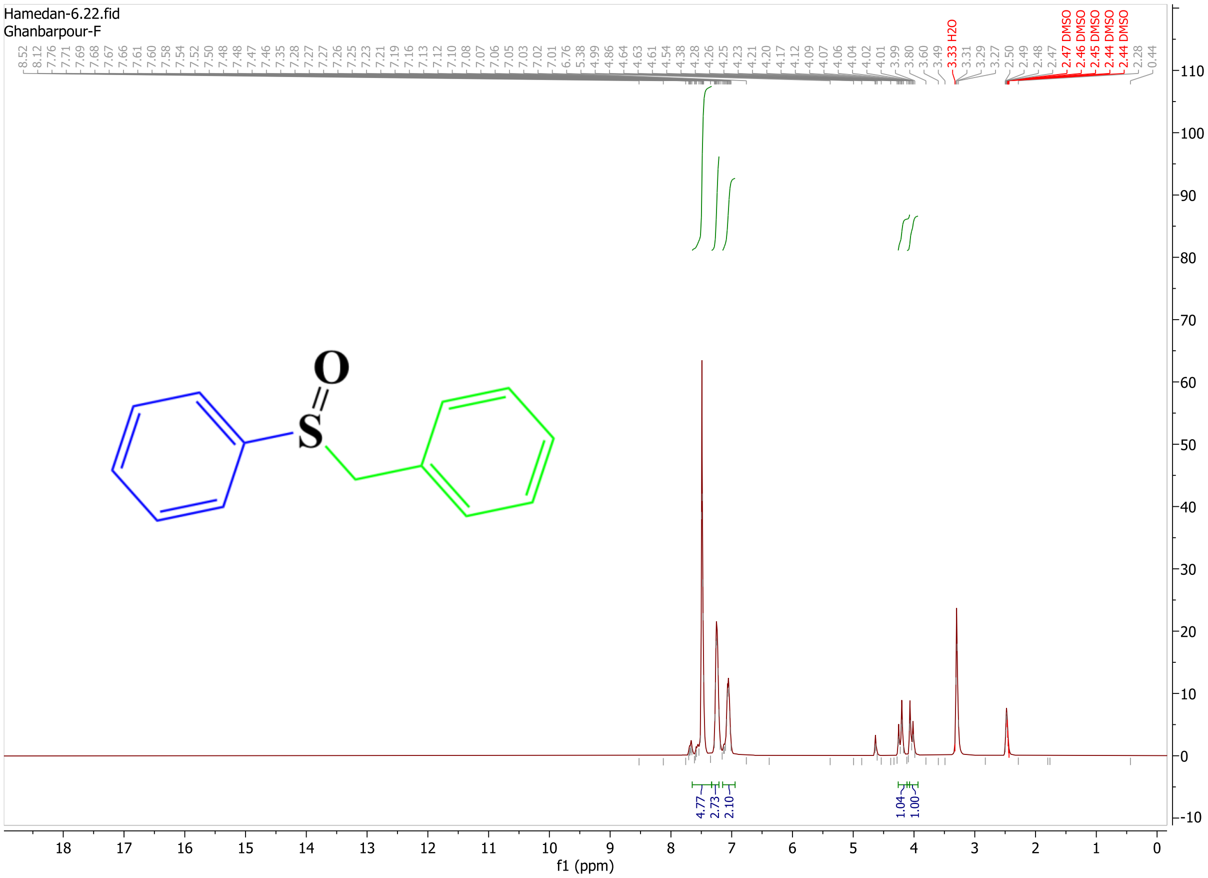

Supplementary figure 47: (benzylsulfinyl)benzene

benzyl(phenyl)sulfane: ^{1}H NMR (250 MHz, DMSO) δ= 4.02 (m, 1H), 4.21 (m, 1H), 7.05-7.66 (m, 10H), ppm.

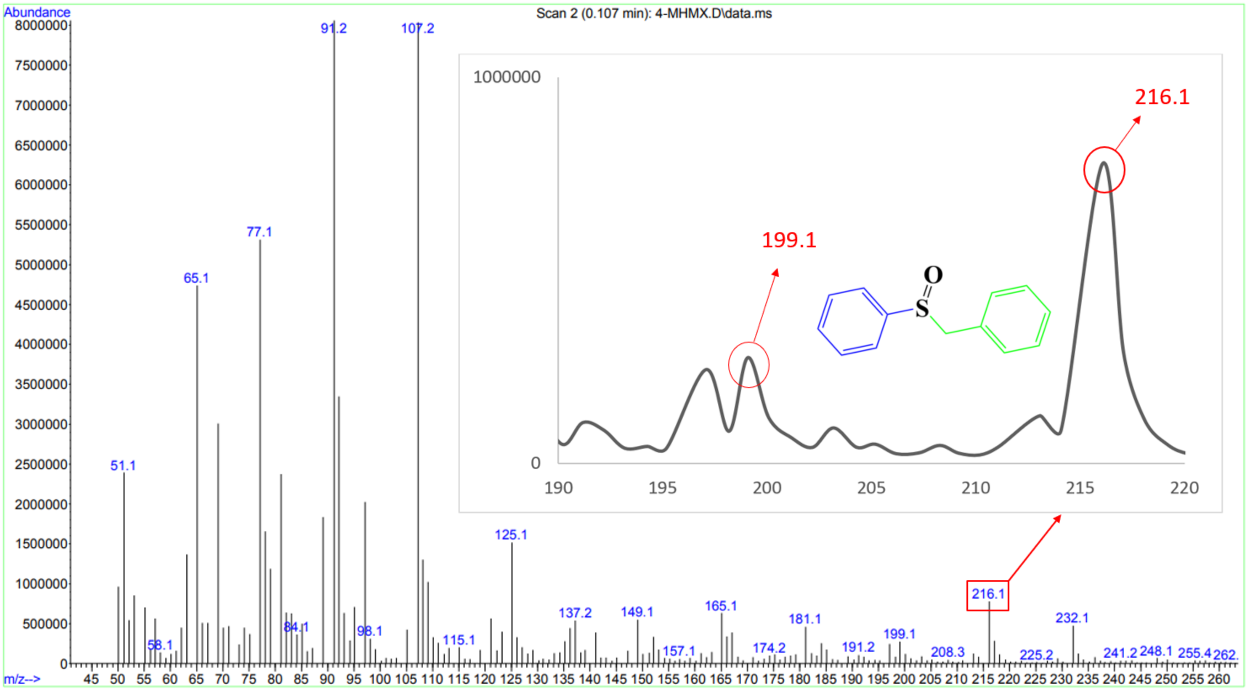

Supplementary figure 48: Mass analysis of (benzylsulfinyl)benzene

Mass analysis: Calculated: M/Z= 216.06, Obtained: M/Z= 216.1

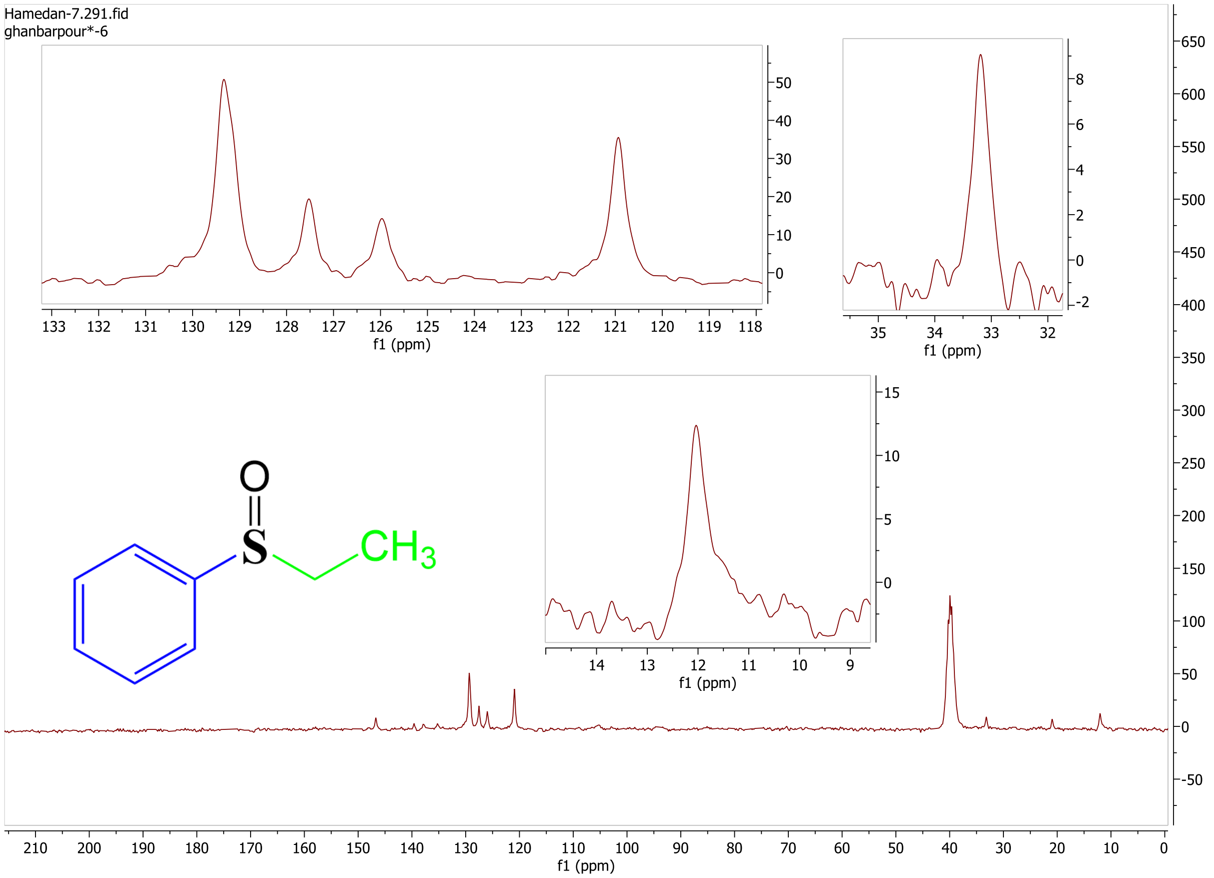

Supplementary figure 49: (ethylsulfinyl)benzene

^{13}C NMR (62.5 MHz, DMSO): 10.5, 34.5, 122.0, 125.3, 126.8, 129.0 ppm

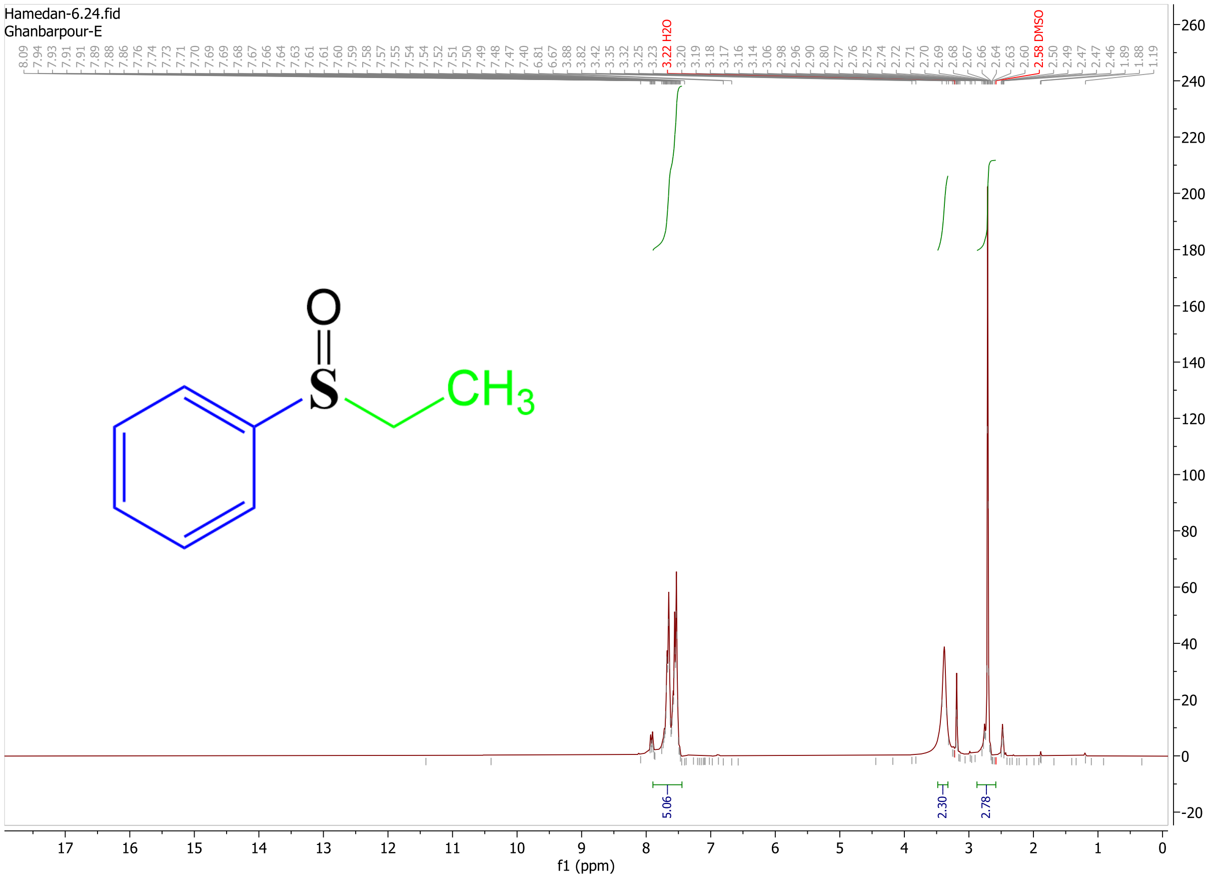

Supplementary figure 50: (ethylsulfinyl)benzene

(ethylsulfinyl)benzene: ^{1}H NMR (250 MHz, DMSO) δ= 2.64 (m, 3H), 3.18 (m, 2H), 7.53-7.93 (m, 5H), ppm.

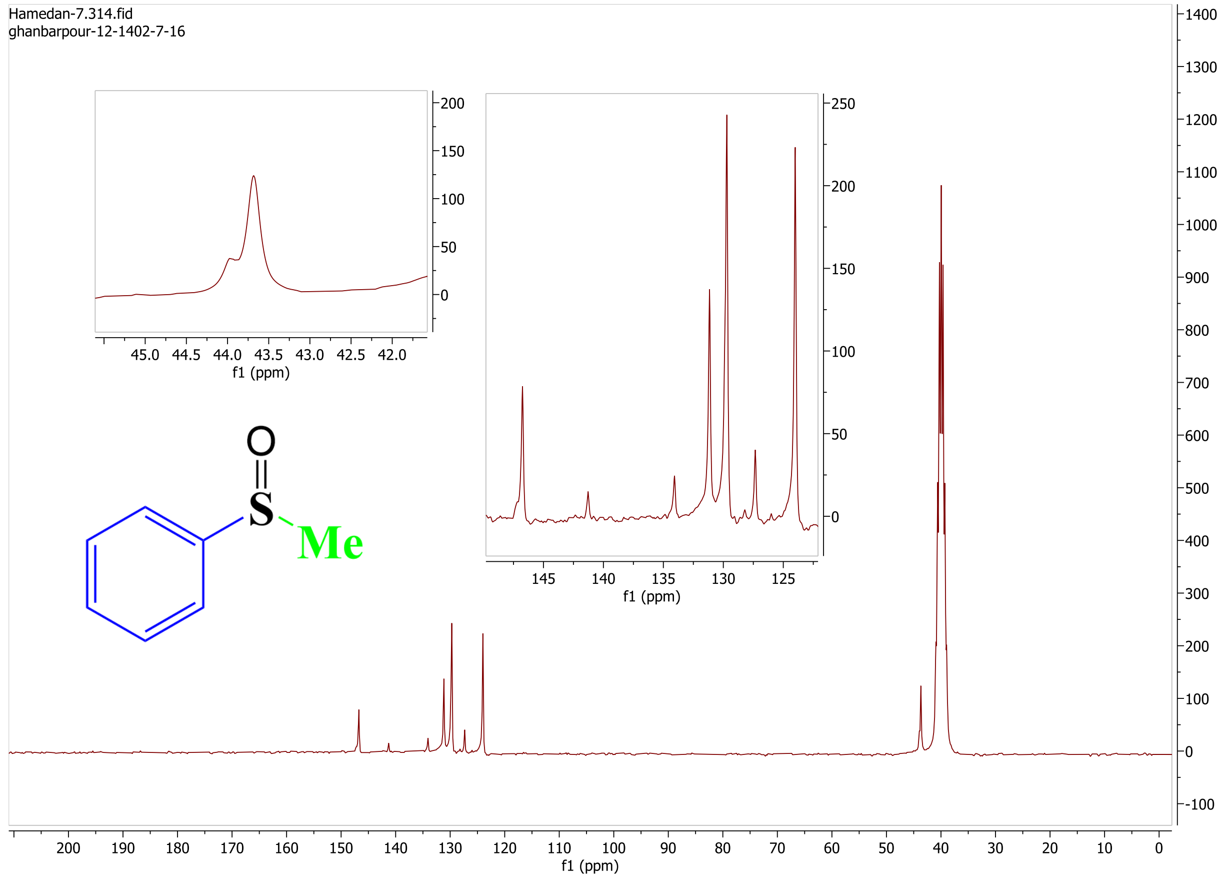

Supplementary figure 51: (methylsulfinyl)benzene

^{13}C NMR (62.5 MHz, DMSO): 43.8, 124.6, 129.2, 133.7, 146.7 ppm.

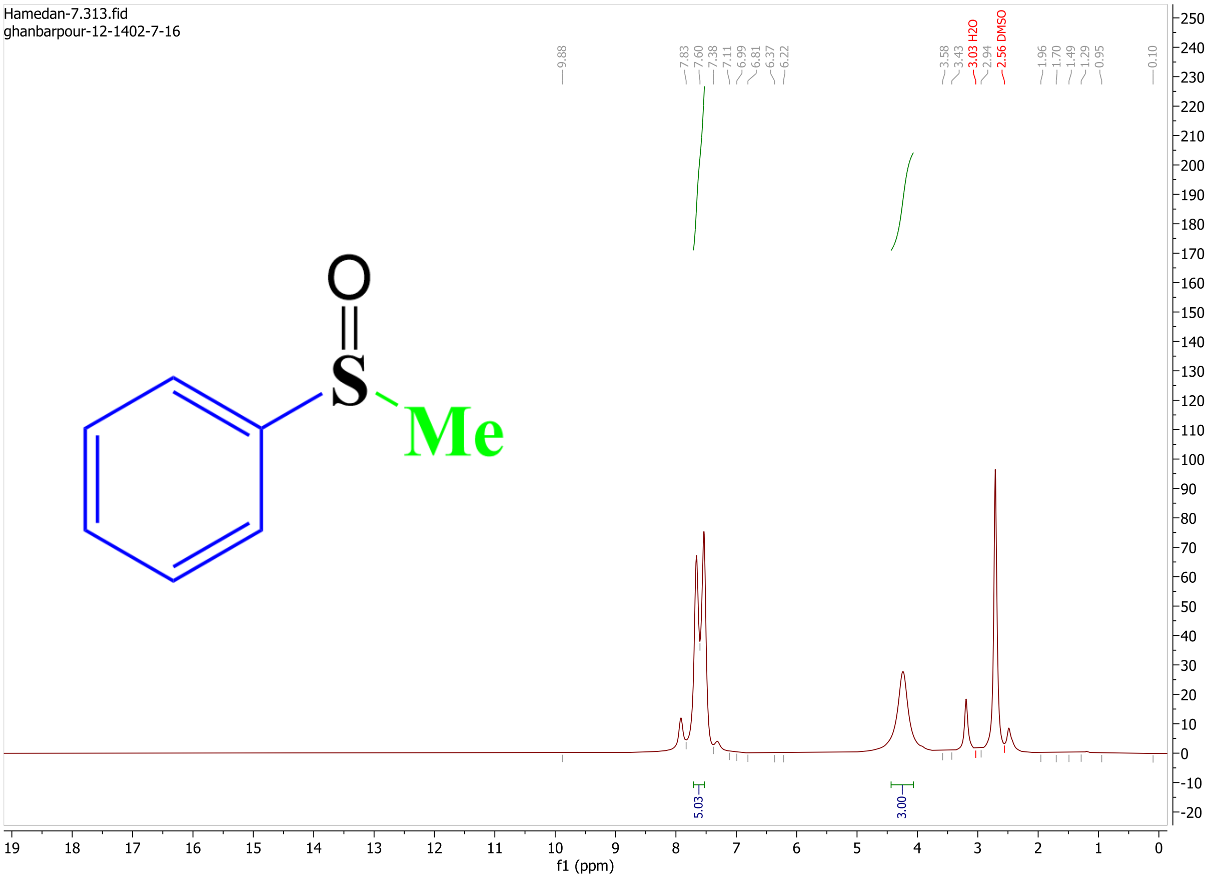

Supplementary figure 52: (methylsulfinyl)benzene

(methylsulfinyl)benzene: ^{1}H NMR (250 MHz, DMSO) δ= 4.22 (s, 3H), 7.38-7.83 (m, 5H) ppm.

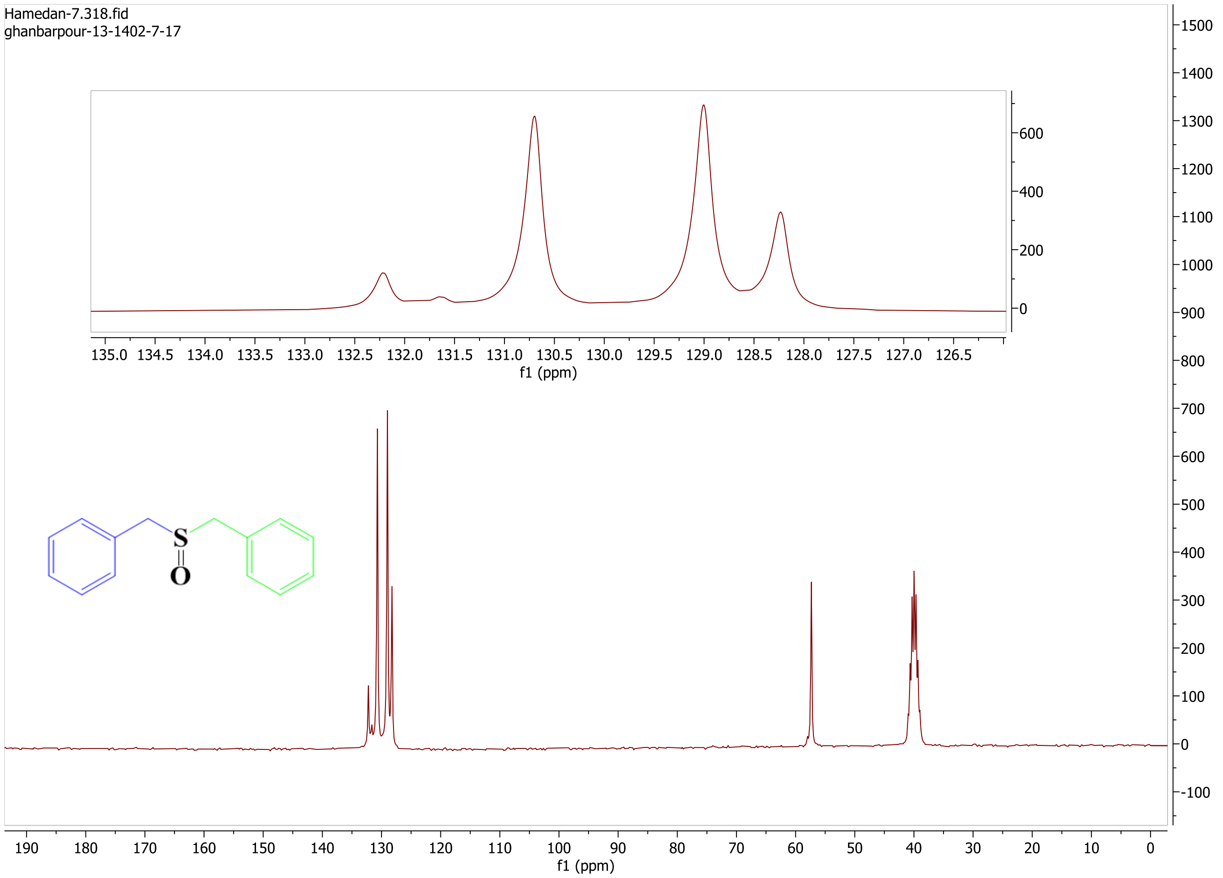

Supplementary figure 53: (sulfinylbis(methylene))dibenzene

^{13}C NMR (62.5 MHz, DMSO): 57.4, 128.1, 129.3, 130.6, 133.6, ppm.

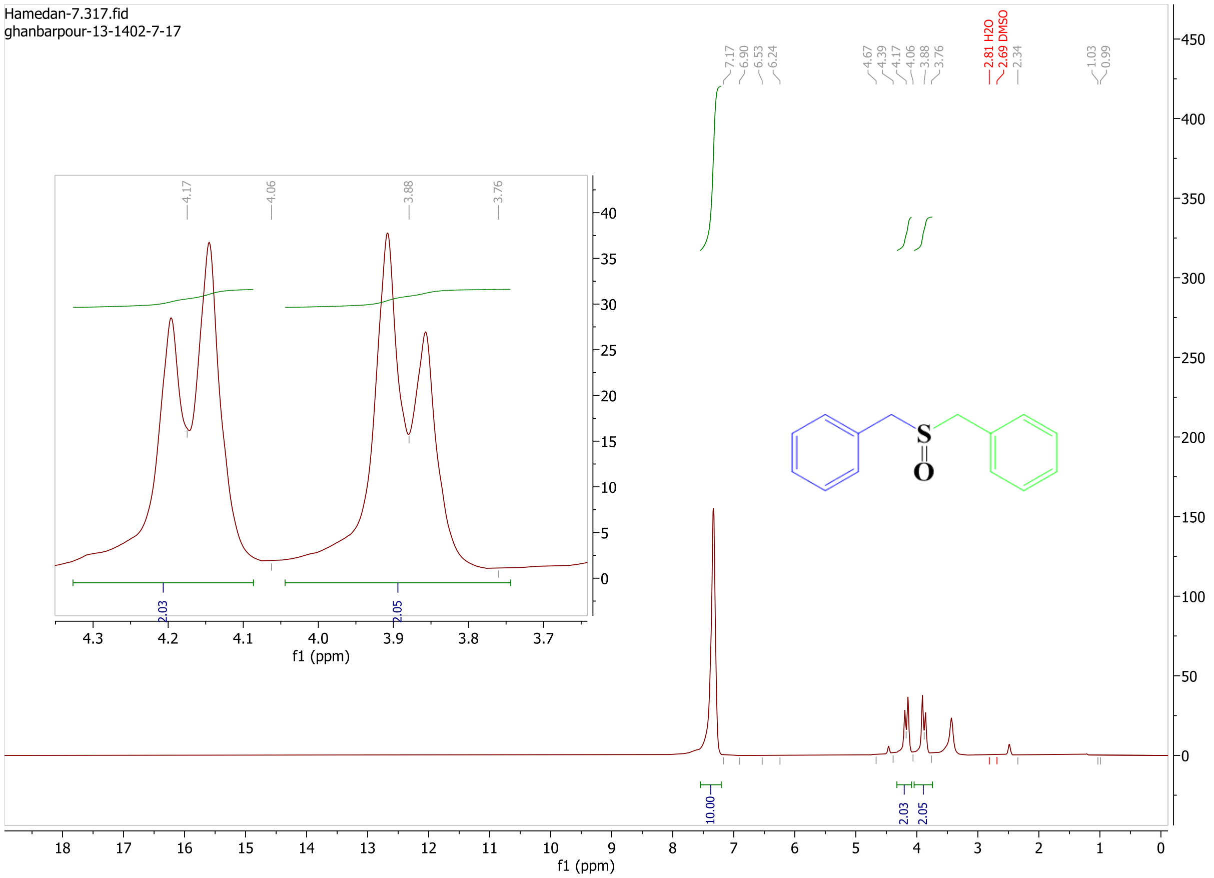

Supplementary figure 54: (sulfinylbis(methylene))dibenzene

(sulfinylbis(methylene))dibenzene: ^{1}H NMR (250 MHz, DMSO) δ= 3.76 (s, 2H), 4.17 (s, 2H), 6.90-7.17 (m, 10H) ppm.

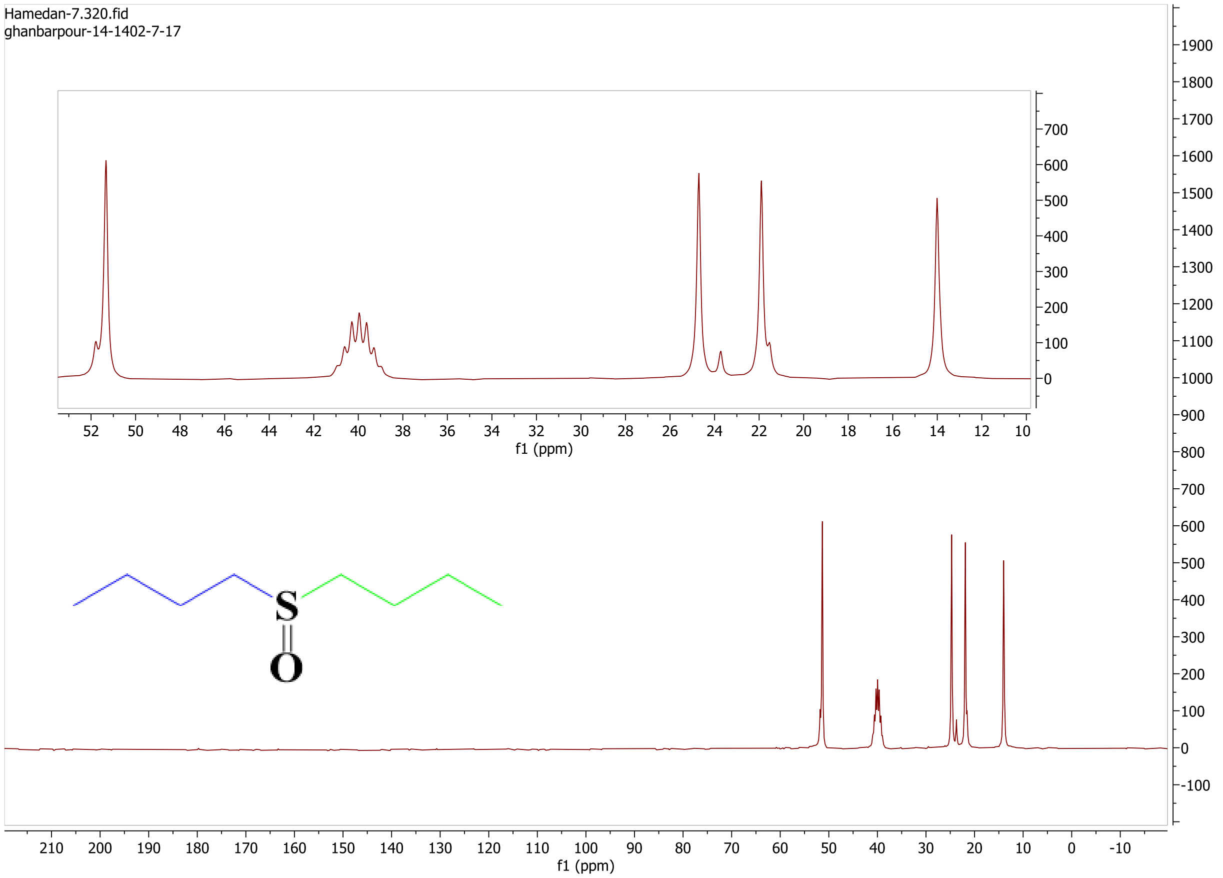

Supplementary figure 55: 1-(butylsulfinyl)butane

^{13}C NMR (62.5 MHz, DMSO): 13.9, 22.0, 25.6, 51.3 ppm.

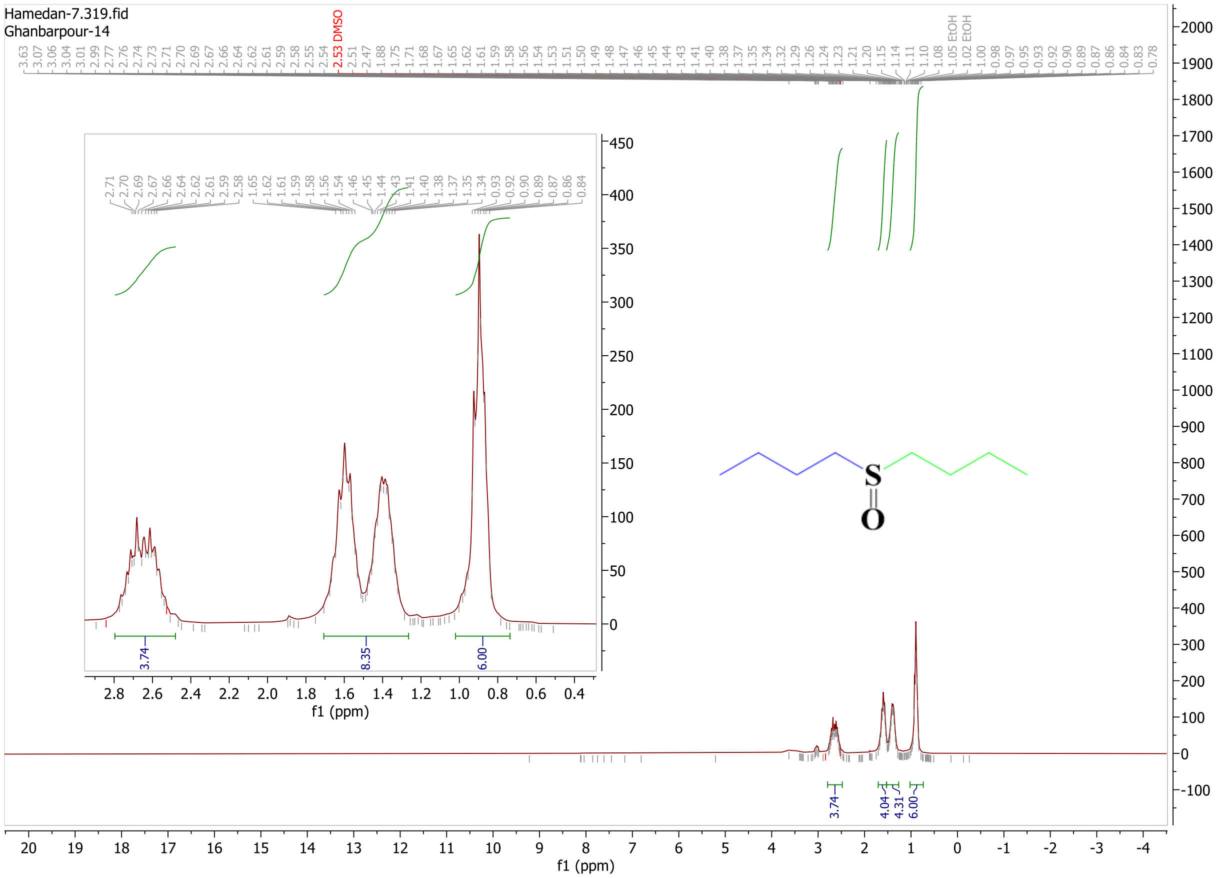

Supplementary figure 56: 1-(butylsulfinyl)butane

1-(butylsulfinyl)butane: ^{1}H NMR (250 MHz, DMSO) δ= 0.97 (m, 6H), 1.40 (s, 4H), 1.53 (s, 4H), 3.04 (m, 10H) ppm.

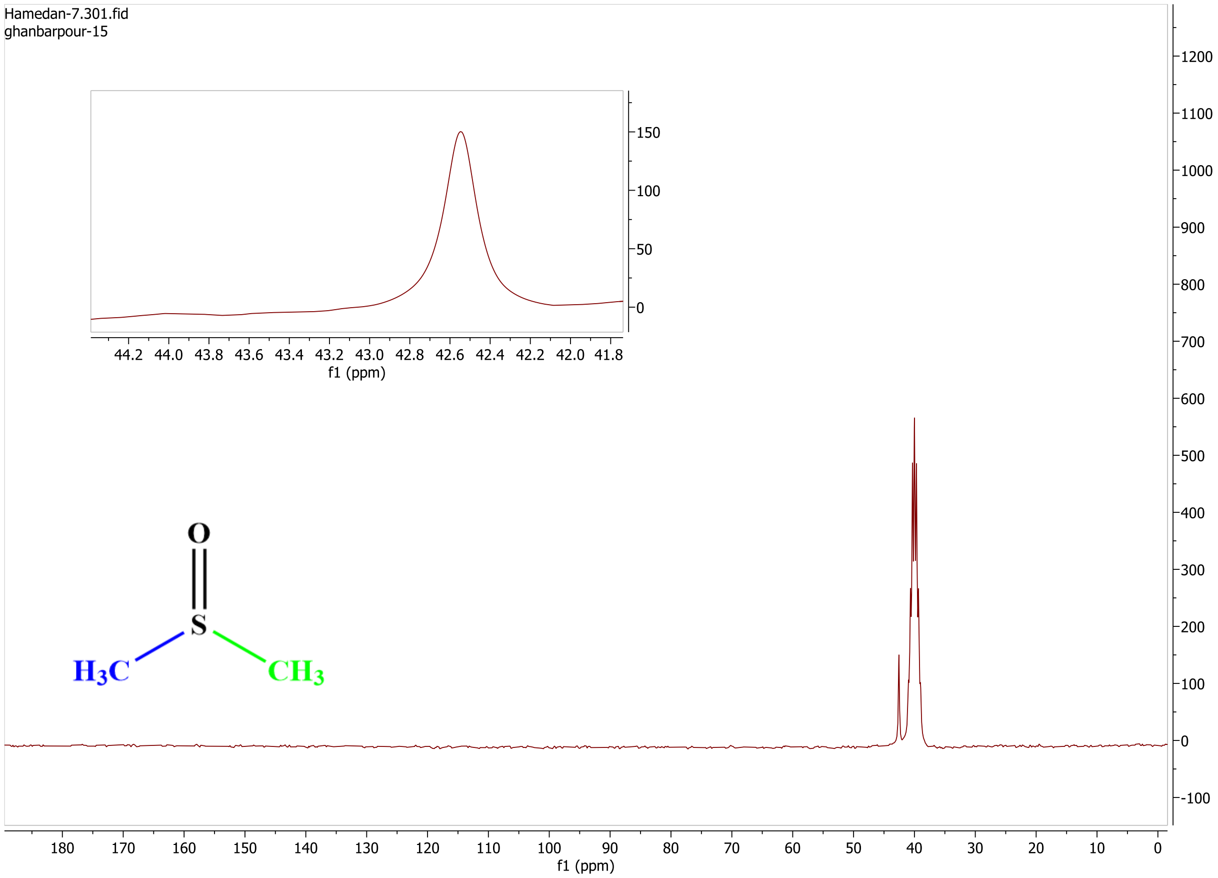

Supplementary figure 57: (methylsulfinyl)methane

^{13}C NMR (62.5 MHz, DMSO): 44.8 ppm.

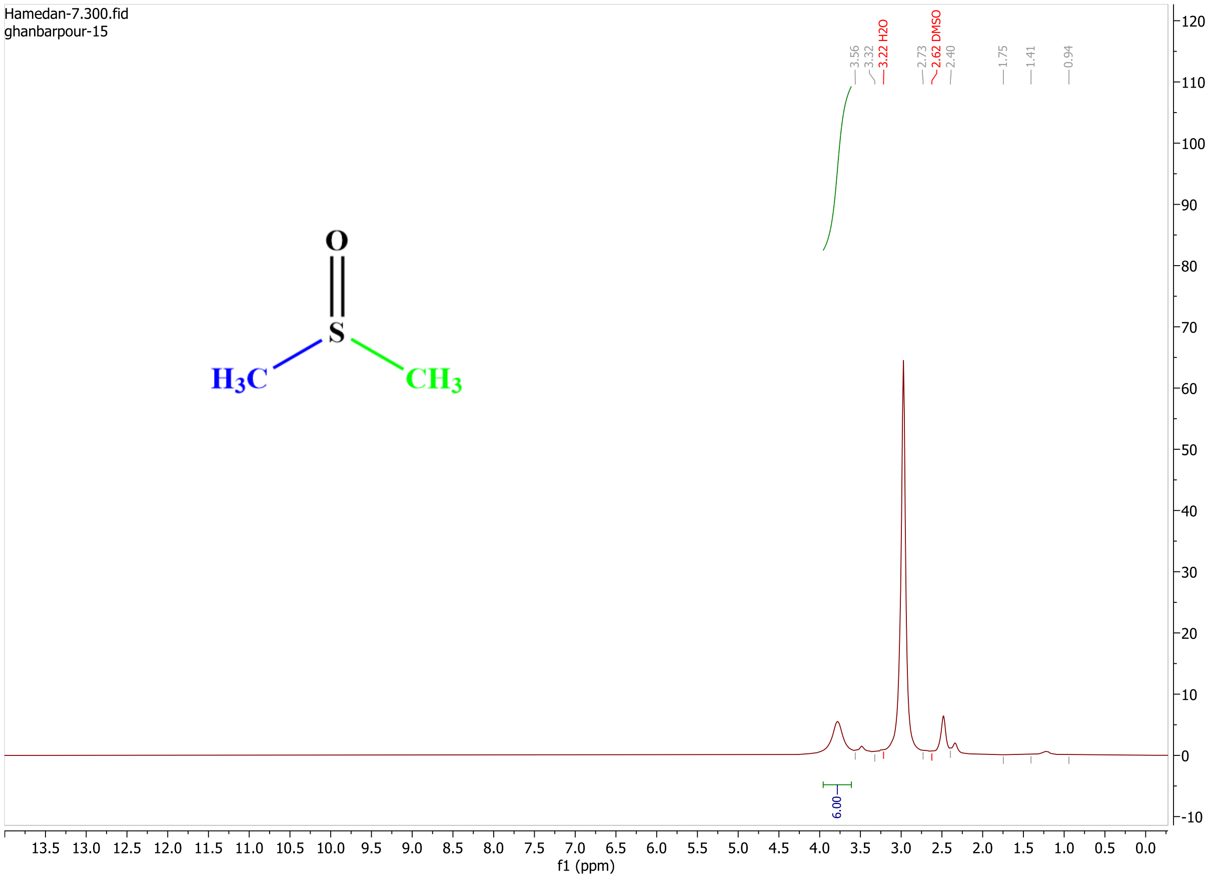

Supplementary figure 58: (methylsulfinyl)methane

(methylsulfinyl)methane: ^{1}H NMR (250 MHz, DMSO) δ= 3.56 (m, 6H) ppm.
